# Boosting Sensory Nerve‐to‐Bone Interactions Enhances Hedgehog Mediated Calvarial Bone Repair

**DOI:** 10.1002/advs.75389

**Published:** 2026-04-20

**Authors:** Zhao Li, Xin Xing, Beicheng Du, Myles Zhou, Austin Z. Chen, Mary Archer, Chunbao Rao, Manyu Zhu, Masnsen Cherief, Aaron W. James

**Affiliations:** ^1^ Department of Pathology Johns Hopkins University Baltimore Maryland USA

**Keywords:** bone regeneration, calvarial defect, hedgehog signaling, neuron–bone crosstalk, sensory neuron, skeletal stem cell, TrkA

## Abstract

Any person that has broken a bone can attest to the existence of sensory innervation of the skeleton. Beyond afferent functions, sensory neurons have been implicated in the orchestration of bone repair via the release of neuroregulatory signals. Yet, these neurosecretory effects have principally been deciphered through loss‐of‐function studies. Indeed, the potential therapeutic benefit of boosting nerve‐to‐bone interactions remains cursorily studied. Here, using a mouse calvarial bone defect model, pharmacologic activation of TrkA with a small molecule partial agonist induced bone‐associated nerve ingrowth and significantly improved calvarial bone healing. Single‐cell RNA sequencing analysis of cells from the defect site revealed shifts in cluster proportions, with enrichment of immune cell populations in TrkA agonist‐treated mice. Within the skeletal cell lineage, TrkA agonism enhanced osteoblast differentiation while suppressing fibroblastic differentiation. Pathway analysis showed increased Hedgehog signaling activity, and interactome analyses between trigeminal ganglia sensory neurons and skeletal cells implicated Hedgehog signaling. The pro‐regenerative effects of TrkA agonism were abolished in conditional knockout mice lacking Smoothened (*Smo*) in PDGFRα^+^ skeletal progenitor cells. In summary, boosting sensory nerve signaling enhances membranous bone repair after injury, at least in part via Hedgehog pathway activation in osteoprogenitor cells.

## Graphical abstract

Boosting sensory nerve activity via TrkA agonism strongly accelerates calvarial bone repair in adult mice. Furthermore, single‐cell RNA sequencing and neuron–bone interactome analyses identify these sensory neurons as a direct neural source of Hedgehog pathway ligands. Consequently, these ligands drive osteoblast differentiation of skeletal progenitors, enhancing local tissue healing through Smoothened‐dependent signaling.

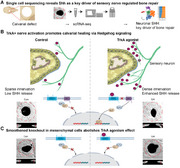

## 1 Introduction

The skeletal system is intimately connected with the peripheral nervous system, a relationship most acutely experienced during bone injury. Bone is richly innervated by sensory nerves [1], and the majority of sensory nerve fibers innervating the skeleton express tropomyosin receptor kinase A (TrkA), the high‐affinity receptor for nerve growth factor (NGF) [2]. While it has been previously established that sensory nerves mediate bone pain primarily through nerve growth factor activating TrkA‐expressing neurons and enhancing nociceptive pathways [3], the broader functions of sensory nerves in bone have only recently begun to be elucidated.

The regulatory roles of sensory nerves in bone metabolism are increasingly recognized. Much focus has been placed on the appendicular skeleton, where sensory innervation participates in long bone development [4] and fracture healing [5, 6, 7]. In comparison, relatively little is known about the dependence of calvarial bones—part of the craniofacial skeleton—on sensory innervation. Craniofacial bones develop primarily through intramembranous ossification [8], and emerging evidence indicates that sensory and sympathetic nerves play critical roles in their regeneration, interacting with bone through neuropeptides, neurotransmitters, and other signaling molecules [9].

For instance, neurotrophic mechanisms involving NGF direct sensory nerve transit in cranial bone, supporting vascularization and osteogenesis [10]. Sensory nerves also regulate mesenchymal progenitor cells in craniofacial bone, influencing remodeling and repair processes [6]. Other groups have also shown TrkA agonism augments the anabolic response to mechanical load [11], and improves long bone fracture healing in mice [12]. However, the key cells and pathways by which TrkA agonism enhances bone regeneration remain undefined.

In this study, we utilized single‐cell RNA sequencing of calvarial injury sites to elucidate the critical pathways through which activation of TrkA signaling enhances bone regeneration. Utilizing mouse calvarial defects as a model, we observed that targeting peripheral nerves to activate TrkA signaling led to robust increases in new bone formation. Through single‐cell RNA sequencing analysis, we demonstrate that activation of TrkA‐positive nerves enhances calvarial defect healing via stimulation of the Hedgehog signaling pathway.

## 2 Results

### 2.1 Small Molecule TrkA Agonism Improves Calvarial Bone Defect Repair

**FIGURE 1:**
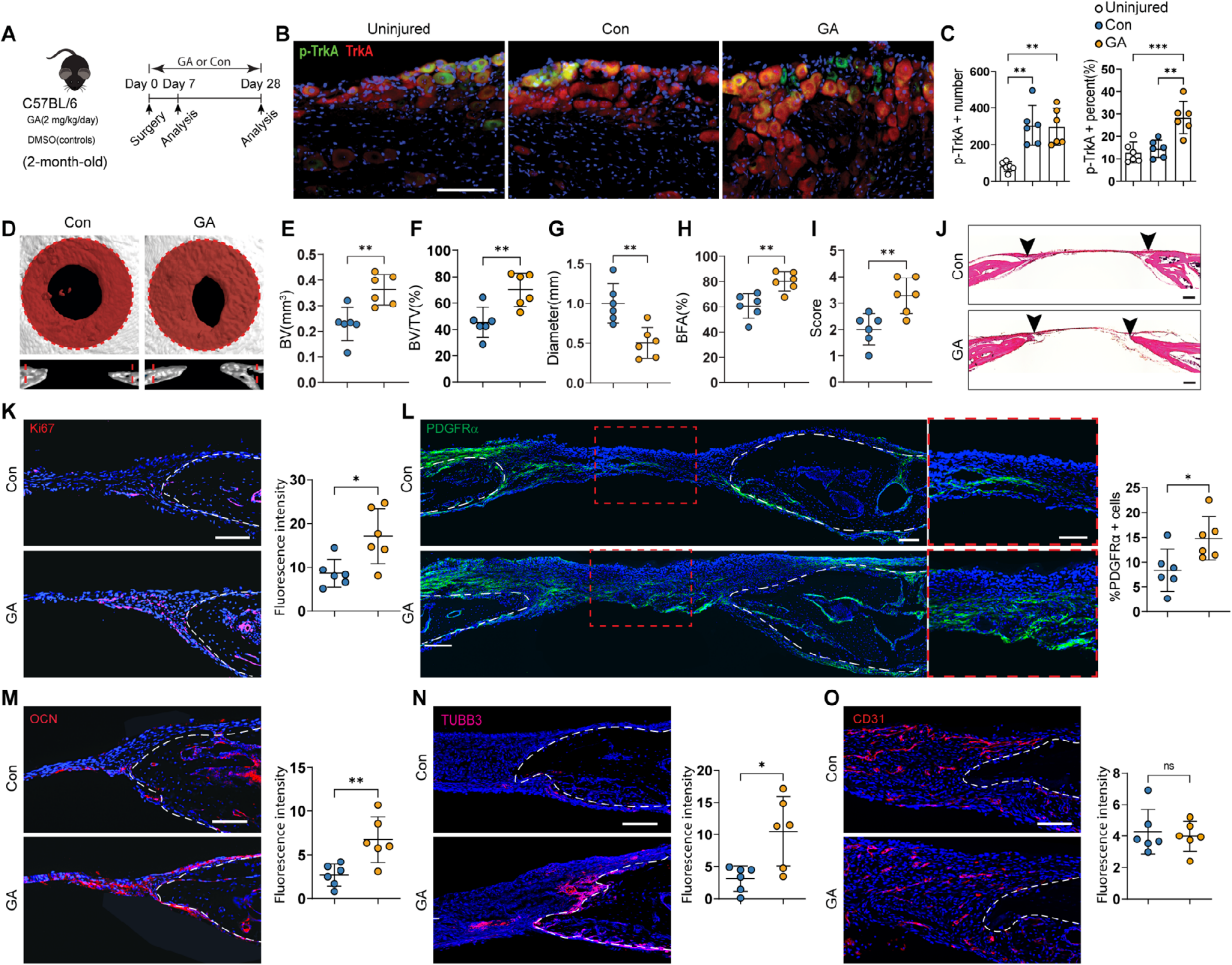
TrkA agonism using the small molecule gambogic amide improves calvarial bone defect repair. (A) Schematic of experiment, in which C57BL/6J mice (male, 2 mo old) were administered Gambogic amide (GA, 2 mg/kg/d IP) or DMSO‐vehicle, followed by frontal bone injury (circular calvarial defect, 1.8 mm diameter), with analysis performed 7 or 28 d post‐operatively. (B) Immunohistochemistry for TrkA (appearing red) and p‐TrkA (appearing green) within trigeminal ganglion sections of uninjured animals, as well as those with calvarial injury treated with either vehicle control or GA (7 d post‐injury). (C) Semi‐quantitative analysis of p‐TrkA positive cell number and p‐TrkA positive cell number percent. (D) Micro‐CT reconstructions of the defect site in a top‐down view (above) and coronal cross‐sectional images (below) among Control and GA treated animals, 28 d post‐injury. Margins of the original defect are indicated by dashed red lines. (E–I) Micro‐CT quantification of bone healing among Control‐ and GA‐treated mice (28 d post‐injury), including (E) Bone volume (BV), (F) Bone volume/tissue volume (BV/TV), (G) residual defect diameter, (H) Bone formation area (BFA), and (I) Bone Healing Score. (J) H&E staining of coronal cross‐section of the healing defect site from control‐ and GA‐treated mice at d28 post‐injury. Black arrowheads indicate healing bone edges. (K) Cellular proliferation at the bone defect edge, as assessed by Ki67 immunofluorescent staining at d7 post‐injury. Dashed white lines indicate the bone edge. (L) Mesenchymal cell migration, as assessed by Pdgfrα immunofluorescent staining. Tile scan (left) and high‐magnification images of the central defect (right) demonstrate migration of PDGFRα+ progenitor cells into the defect site at d7 post‐injury. (M–O) Immunohistochemical staining and semi‐quantitative analysis of Osteocalcin (OCN) 28 d post‐injury (M), Beta III Tubulin (TUBB3) (N), and CD31 (O) within the calvarial defect, d7 post‐injury. Dashed white lines indicate bone edges. In graphs, each dot represents a single animal. Black scale bar: 500 µm. White scale bar: 100 µm. N = 6 mice per group. Data are represented as mean ±1 SD. ns: non‐significant. ^*^ *P*<0.05, ^**^ *P*<0.01, and ^***^ *P*<0.001 as assessed using a two‐tailed Student's *t*‐test.

First, gambogic amide (GA), a small molecule TrkA agonist, was provided to mice during the process of cranial bone defect repair to promote local neuron growth and defect site re‐innervation using previously validated dosing (Figure 1A, 2mg/kg daily) [13]. Sections of the trigeminal ganglia were first examined, 7 days after injury (Figure 1B,C). Immunohistochemical staining of TrkA and phosphorylated TrkA (pTrkA) revealed that the number of pTrkA‐positive cells increased 276.2% after injury. Additionally, there was a notable 95.7% increase in the percentage of pTrkA‐positive cells within the trigeminal ganglion among GA‐treated mice in comparison to control (Figure 1B,C). Micro‐CT reconstructions and cross‐sectional images within the calvarial bone defect site demonstrated improved bone defect repair in GA treated animals in comparison to control (Figure 1D). Quantitative micro‐CT metrics of bone repair were next examined among GA treated mice (Figure 1E–I). Results showed a 59.8% increase in BV (Figure 1E), and a 55.1% increase in BV/TV (Figure 1F). Conversely, a 49.5% reduction in the residual diameter of the bone defect was observed (Figure 1G). This corresponded to a 32.2% increase in BFA (Figure 1H), and a significant increase in bone healing score. (Figure 1I). H&E staining confirmed notable bone defect healing between bony fronts in GA treated mice (Figure 1J, black arrowheads). To investigate the mechanisms underlying this bone repair process in GA treated mice, we first conducted immunohistochemical staining of Ki67, PDGFRα, OCN, TUBB3, and CD31 to determine potential differences in proliferation, skeletal progenitor cell migration, ossification, innervation, and revascularization 28 days post‐injury (Figure 1K–O). Ki67 and PDGFRα immunostaining of mesenchymal cells demonstrated a 98.2% increase and a 76.9% increase, respectively (Figure 1K,L). IHC staining of OCN showed a significant 151.1% increase in the number of mature osteoblasts among GA treated mice (Figure 1M). A significant 233.9% increase in TUBB3+ nerve fiber density was observed in GA treated mice compared to the control at 7 days post‐injury (Figure 1N). CD31 staining showed comparable vascular density between groups (Figure 1O). Taken together, Gambogic amide's selective binding to TrkA and triggering of its tyrosine phosphorylation initiated nerve changes in GA treated mice. These changes in the degree of injury site innervation appeared to be associated with a dramatic increase in proliferation, skeletal progenitor cell migration, and ossification within the bone injury site, leading to a robust improvement in calvarial bone defect repair.

### 2.2 Single‐Cell Transcriptomic Profiling of Total Cells and Mesenchymal Lineage Subpopulation

**FIGURE 2:**
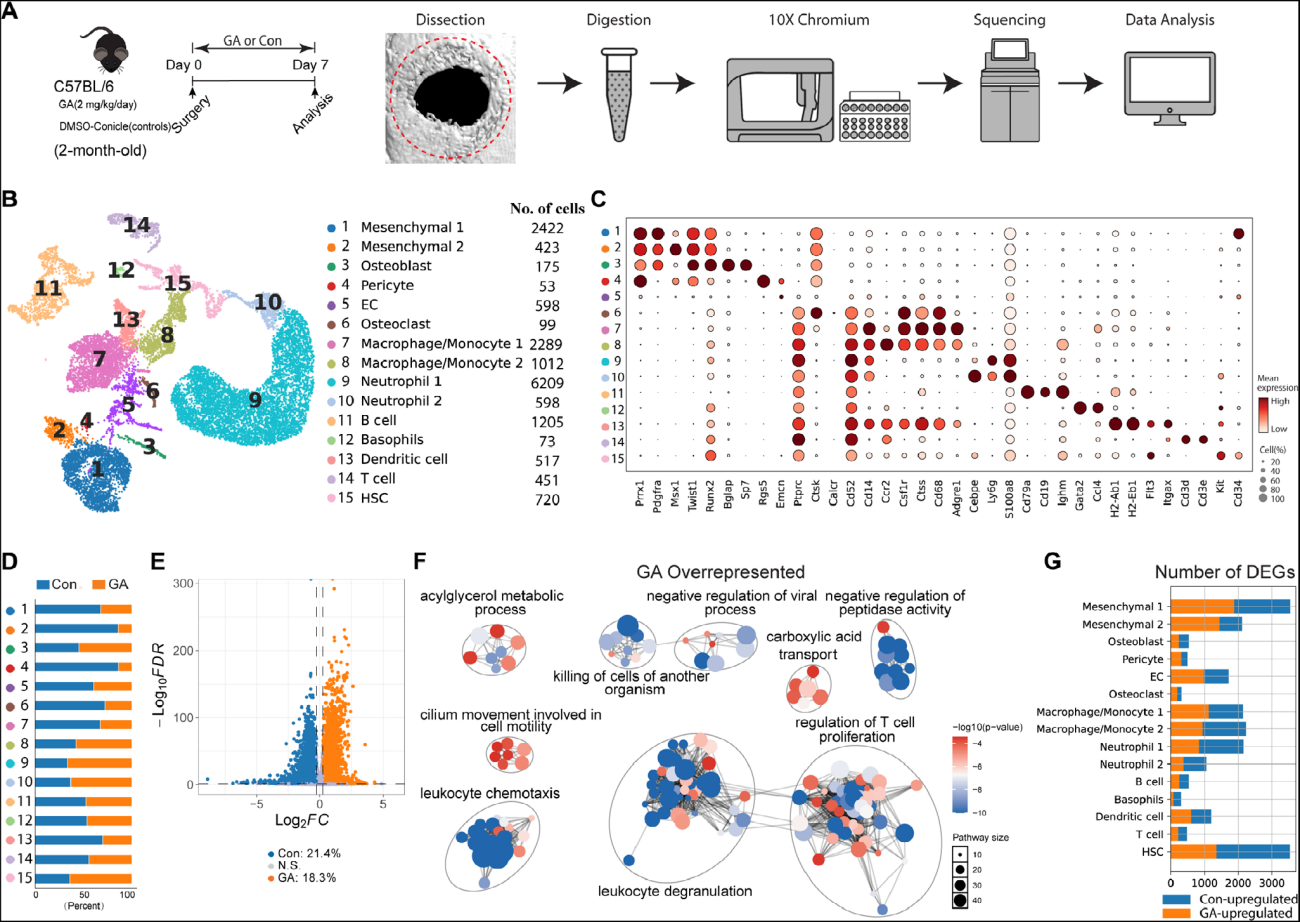
Single‐cell transcriptomic profiling of defect‐associated cells in animals treated with gambogic amide (GA). (A) Schematic of experiment, in which C57BL/6J animals (male, 2 mo old) were administered gambogic amide (GA) or vehicle control, followed by frontal bone injury (circular calvarial defect, 1.8 mm diameter), with scRNA‐Seq analysis performed 7 post‐operatively. The defect area was microdissected, dissociated, and subjected to scRNA‐seq. (B) Uniform manifold approximation and projection (UMAP) plot of total cells isolated from calvarial defect site among control‐ and GA‐treated animals, and cell frequency per cluster. (C) Dotplot of marker gene expression. mesenchymal lineage cells [expressing Paired related homeobox 1 (*Prrx1*), Platelet‐derived growth factor receptor alpha (*Pdgfra*), Msh homeobox 1 (*Msx1*), Twist family BHLH transcription factor 1 (*Twist1*), and Runt‐related transcription factor 2 (*Runx2*)], hematopoietic cells [expressing Protein tyrosine phosphatase receptor type C (*Ptprc*) encoding CD45], endothelial cells/pericytes [expressing Regulator of G‐protein signaling 5 (*Rgs5*) and Endomucin (*Emcn*)]], erythrocyte [expressing Hemoglobin subunit alpha 2 (*Hba‐a2*) and Hemoglobin subunit alpha 1 (*Hba‐a1*)]], hematopoietic stem cells (HSC) [expressing KIT Proto‐Oncogene, Receptor Tyrosine Kinase (*Kit*) and CD34 molecule (*Cd34*). (D) Stacked bar plot of the distribution of the clusters among control‐ and GA‐treated animals. (E) Volcano plot of overrepresented genes in control and GA groups. Significantly differentiated expressed genes are identified using thresholds with FDR < 0.05 and Log2FC > 0.25. (F) GO enrichment map of significantly enriched biological processes (GA Overrepresented GO terms) among all cells from control‐ and GA‐treated bone defects. Nodes represent gene‐sets and edges represent GO‐defined relations. Clusters are annotated according to the corresponding function. (G) Number of differentially expressed genes (DEGs) identified across cell clusters. n = 5 mice per group were used to yield a single‐cell population.

To understand the mechanisms driving defect repair in TrkA agonist‐treated mice, we used single‐cell RNA sequencing (scRNA‐seq) of cells derived from the defect site of control and GA treated mice at the early timepoint of 7 days after injury (Figure 2A). Unsupervised clustering identified sixteen cell clusters (Figure 2B), identified using expression patterns of known marker genes, including three clusters of mesenchymal lineage cells [expressing *Prrx1*, *Pdgfra*, *Msx1*, *Twist1*, and *Runx2*], nine clusters of hematopoietic cells [expressing *Ptprc*], two clusters of endothelial cells/pericytes [expressing Rgs5 and Emcn], and one cluster of hematopoietic stem cells (HSC) [expressing *Kit* and *Cd34*] (Figure 2C). While each cluster was represented by cells from both control and GA treated mice, some shifts in cluster proportions were observed, including enrichment of immune cells, and osteoblasts cluster cells derived from GA treated mice (Figure 2D; Figure S1A). The volcano plot illustrates the distribution of DEGs across the entire dataset (Figure 2E). Of a total of 24 495 genes, 4477 genes (18.3%) of genes significantly were overrepresented under the GA group, while 5246 genes (21.4%) were overrepresented under Control conditions (Figure 2F; Table S3). Gene ontology (GO) analyses of upregulated genes in the GA treated mice group demonstrated enrichment in GO terms associated with cellular immunity, such as leukocyte chemotaxis, leukocyte degranulation, and regulation of T cell proliferation (Figure 2F; Table S4). Analysis of differentially expressed genes (DEGs) across distinct cell clusters revealed that mesenchymal 1 exhibited the highest number of DEGs (3535 genes). This was followed by the HSC (3527 genes) and mesenchymal 2 (2113 genes). Among immune cell populations, the macrophage/monocyte and neutrophil clusters displayed substantial numbers of DEGs. Given the pronounced transcriptional alterations observed in mesenchymal cells, macrophages, and neutrophils, these three cell populations were selected for subsequent in‐depth analysis (Figure 2G).

**FIGURE 3:**
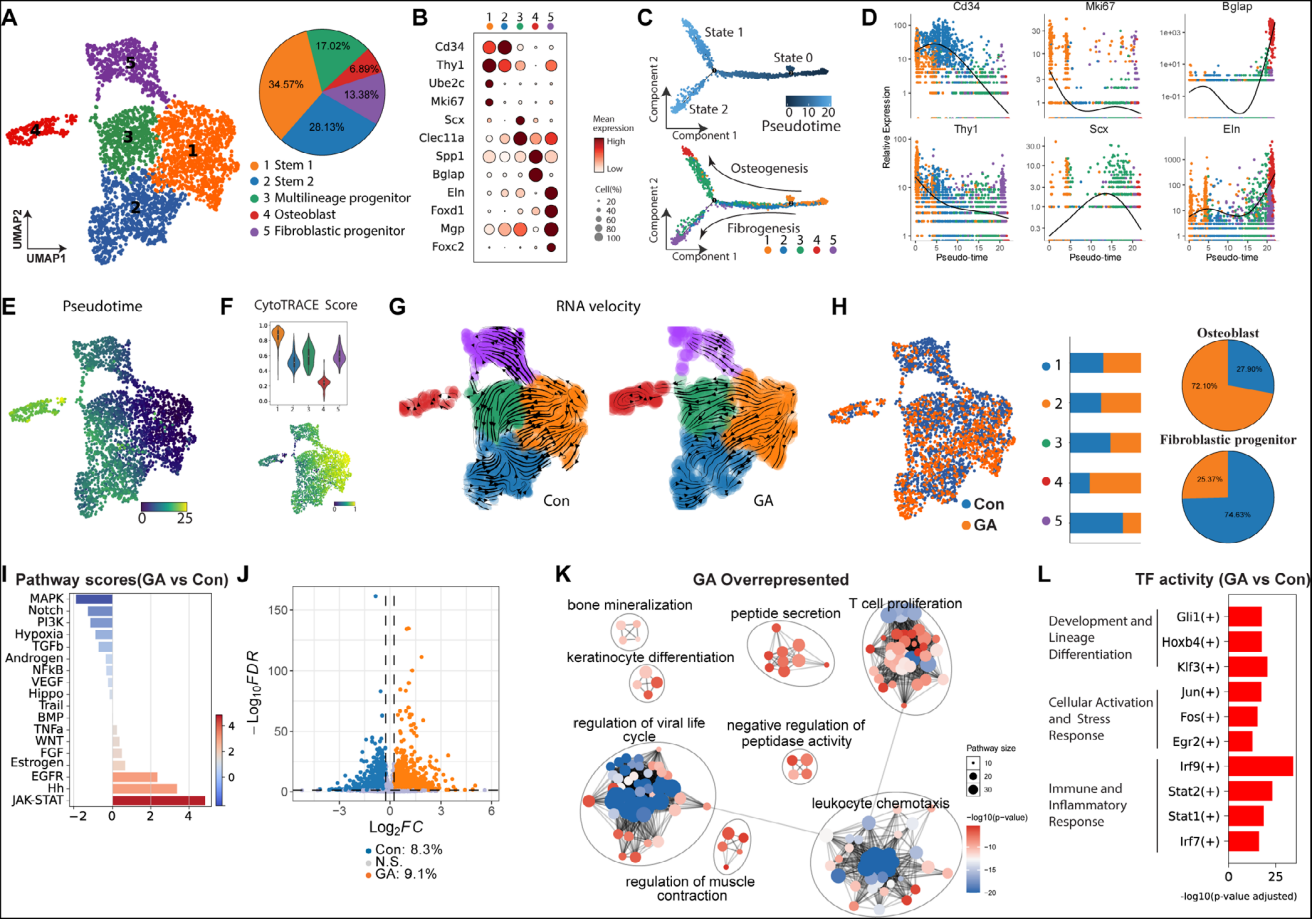
TrkA agonism induces a shift toward osteoblastic and away from fibroblastic cell fates within the bone injury site. (A) UMAP plots and the percent of each cluster. (B) Dot plot of key marker gene expression across clusters. Stem cell[*C* *d* *34* and Thy‐1 cell surface antigen(*Thy1*)], Stem cell 1[ubiquitin‐conjugating enzyme E2C (*Ube2c*) and antigen identified by monoclonal antibody Ki‐67 (*Mki67*)], Multilineage progenitor[scleraxis (*Scx*) and C‐type lectin domain family 11 member A (*Clec11a*)], Osteoblast cells[bone gamma‐carboxyglutamate protein (*Bglap*) and secreted phosphoprotein 1 (*Spp1*)], Fibroblastic progenitor[Elastin (*Eln*), Forkhead box D1(*Foxd1*), Matrix Gla Protein (*Mgp*) and Forkhead Box Protein C2 (*Foxc2*)]. (C) Trajectory of mesenchymal cells to osteoblasts inferred by Monocle2 and colored by cell clusters. Each dot indicates a single cell, with color coding showing cell clusters. (D) Plots showing the relative expression of key genes in pseudotime order. (E) UMAP plot of pseudotime value. (F) (Top) UMAP plot of Cytotrace scoring. (Bottom) Violin plots of Cytotrace scoring from mesenchymal subclusters. (G) RNA velocity analysis. UMAP with averaged RNA velocity vectors embedded. (H) Distribution in UMAP plot and percentage of osteoblast and fibroblastic progenitor from control and GA treated mice. (I–K) Comparisons of pathway activity, gene ontology enrichment, and transcription factor activity changes between GA treated and control conditions, specifically in the stem1, stem2, and multilineage progenitor cell clusters. (I) Bar plot of the change of pathway activity. (J) Volcano plot of overrepresented genes in control and GA groups. Significantly differentiated expressed genes are identified using thresholds with FDR < 0.05 and Log2FC > 0.25. (K) GO enrichment map of significantly enriched biological processes (GA Overrepresented GO terms) among all defect‐associated cells from control‐ and GA‐treated bone defects. (L) Bar plot of change of transcription factor (TF) activity.

Focused analysis of mesenchymal lineage cells using unsupervised clustering identified five subclusters (Figure 3A). Early stem cell markers, *Cd34* and *Thy1*, were predominantly expressed in subclusters 1 and 2, designated as stem cell subclusters ‘Stem 1’ and ‘Stem 2’. Cell proliferation markers, such as *Ube2c* and *Mki67*, were predominantly expressed in subcluster ‘Stem 1.’ Multiple different lineage markers, such as *Scx* [14] and *Clec11a* [15], showed enriched expression in subcluster 3, and were designated the ‘multilineage progenitor’ subcluster (Figure 3B). Osteoblasts in subcluster 4 were found to express mature osteoblast markers such as *Bglap* and *Spp1*. Subcluster 5 was classified as a likely fibroblastic progenitor due to the high expression of markers of *Eln* [16] and *Foxd1* [17], also with expression of dural fibroblasts markers such as *Mgp* [18] and *Foxc2* [19] (Figure 3B). Trajectory analysis revealed potential differentiation trajectories toward either osteogenesis (state 1) and fibrogenesis (state 2), with a predicted trend of progressive change from subcluster stem 1 to osteoblasts and fibroblastic progenitors (Figure 3C). Concomitantly, genes highly expressed in stem 1 (such as *Cd34* and *Thy1*) were gradually downregulated, while the proliferation marker *Mki67* exhibited higher expression at initial timepoints. In contrast, characteristic genes in multilineage progenitors, osteoblasts, and fibroblastic progenitor (e.g., *Scx*, *Bglap2*, and *Eln*) were upregulated upon terminal osteoblast differentiation along the trajectory (Figure 3D). Pseudotime visualization on a UMAP plot confirmed these findings, with Stem 1 subclusters showing the lowest pseudotime value and osteoblasts showing the highest (Figure 3E). To validate the differentiation states of the individual clusters, CytoTRACE algorithm was also employed, which identified the Stem 1 cluster as the population in the most undifferentiated state (Figure 3F). To resolve the differentiation dynamics and infer the directionality of individual cells during differentiation between the control and GA groups, RNA velocity analysis was performed, which similarly identified Stem 1 as the root cell population. RNA velocity analysis predicted two main developmental trajectories. The first originated from stem 1 and proceeded toward multilineage progenitors, terminating at osteoblasts. The second main differentiation path, which also originated from stem 1, was directed toward and ended at fibroblastic progenitors. These findings were consistent with the results of trajectory analysis. The control group exhibited a weaker velocity toward osteoblast differentiation but a stronger velocity toward fibroblastic progenitor differentiation, whereas the GA treated group displayed the opposite pattern. (Figure 3G). Comparison between GA treated and control groups showed 158% more osteoblasts, but 66% fewer fibroblastic progenitors in the GA group compared to controls (Figure 3H). Pathway enrichment analysis using the multivariate linear model (mlm) method [20] with the PROGENy model [21] and published gene sets [14, 22] revealed differential pathway activity. Compared to the control group, the GA treated group exhibited increased pathway activity in several known bone anabolic pathways. This included several pathways whose ligands are known to be expressed in somatosensory nerves, including Hedgehog (Hh) signaling [23], fibroblast growth factor (FGF) signaling [24], WNT and bone morphogenetic protein (BMP) signaling, among others (Figure 3I). A volcano plot illustrates the distribution of DEGs across the entire dataset (Figure 3J). Of a total of 22 166 genes, 2016 genes (9.1%) were significantly overrepresented in cells of the GA treated group, while 1837 genes (8.3%) were overrepresented in cells derived from the control groups (Figure 3J; Table S5). Gene ontology (GO) analyses of upregulated genes in the GA treated mice group demonstrated enrichment in GO terms associated with bone mineralization as well as multiple terms related to inflammation and immunoregulation (Figure 3K; Table S6). Analysis of transcription factor (TF) activity in Stem 1 and Stem 2 subclusters revealed significant upregulation in the GA group compared to the control. The top 10 enhanced TFs included GLI family zinc finger 1 (*Gli1*), a key regulator of development and lineage differentiation. The remaining enriched TFs comprised developmental regulators (*Hoxb4* and *Klf3*), stress response factors (*Jun*, *Fos*, and *Egr2*), and several immune and inflammatory mediators (*Irf9*, *Stat2*, *Stat1*, and *Irf7*). (Figure 3L). Considering the no expression of TrkA in mesenchymal cells (Figure S3A,B), suggesting that the observed, robust transcriptional changes in mesenchymal cells are likely based on indirect effects via the stimulation by other TrkA+ cell types, such as neurons.

### 2.3 Single‐Cell Transcriptomic Profiling of Bone Defect‐Associated Neutrophils and Macrophages

**FIGURE 4:**
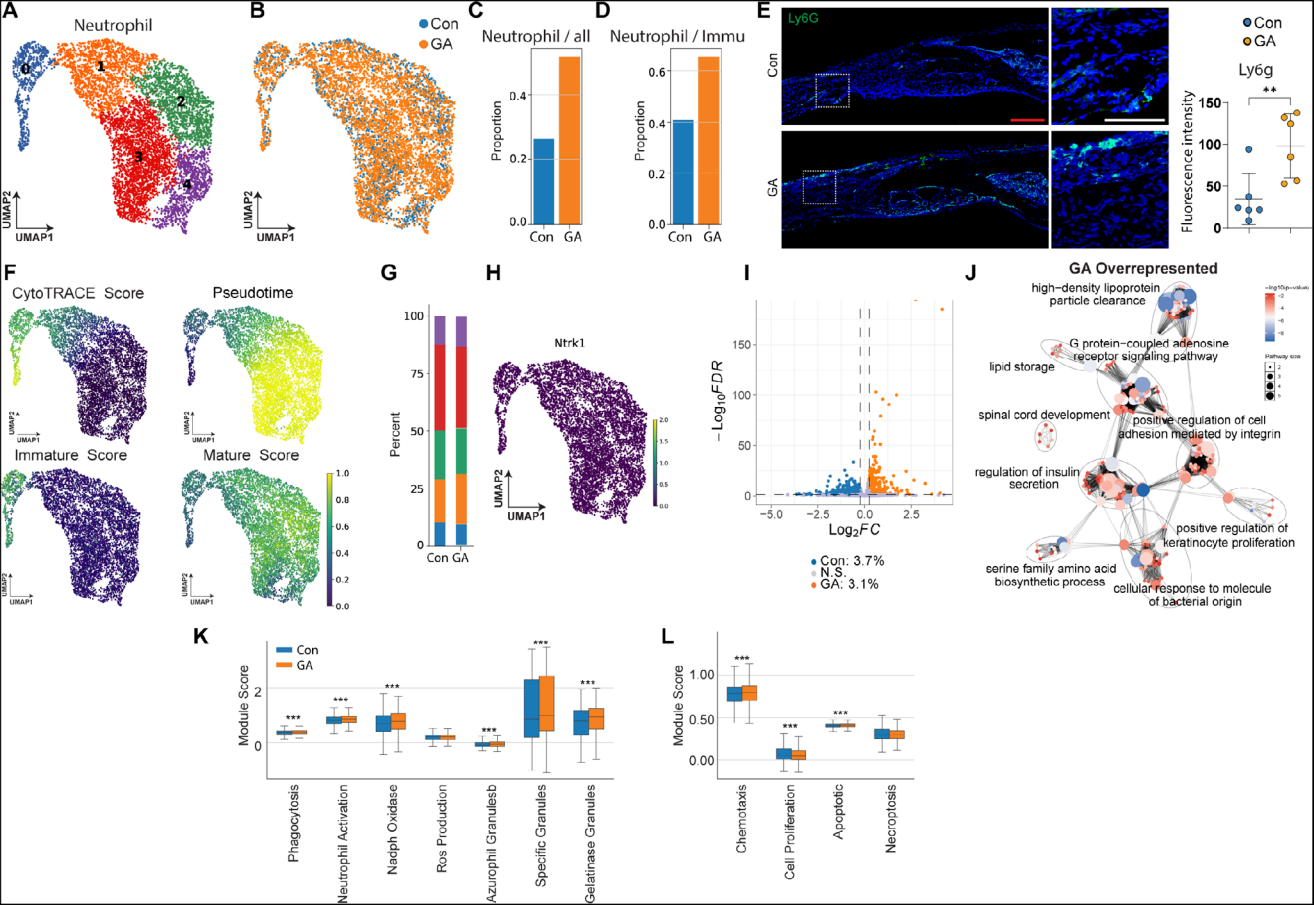
Single‐cell transcriptomic profiling of bone defect‐associated neutrophils. (A)  UMAP plots of each cluster. (B) Distribution in the UMAP plot of control and GA treated mice. (C) Bar plot of the proportion of neutrophils in all cells. (D) Bar plot of the proportion of neutrophils in all immune cells. (E) Immunohistochemical staining and semi‐quantitative analysis of lymphocyte antigen 6 family member G (*Ly6g*) within the calvarial defect, 7 d post‐injury. Dashed white lines indicate the bone edge. (F) UMAP plot of CytoTRACE Score, Pseudotime, Immature Score, and Mature Score. (G) Stacked bar plot of the distribution of the clusters among Control and GA animals. (H) Feature plot illustrating the expression of Ntrk1 projected onto the UMAP. (I) Volcano plot of overrepresented genes in control and GA groups. Significantly differentiated expressed genes are identified using thresholds with FDR < 0.05 and Log2FC > 0.25. (J) GO enrichment map of significantly enriched biological processes (GA Overrepresented GO terms) among all neutrophil cells from control‐ and GA‐treated bone defects. (K) Box plots of neutrophil function module scores. (L) Box plots of neutrophil dynamics module scores. In graphs, each dot represents a single animal. Red scale bar: 100 µm. White scale bar: 50 µm. N = 6 mice per group. Data are represented as mean ±1 SD. ^**^ *P*<0.01 as assessed using a two‐tailed Student's *t*‐test.

Having discovered the relative enrichment of neutrophils and macrophages in calvarial defects of GA treated mice, we further investigated the potential roles of these immune cells in the enhanced bone repair process in GA treated mice. We first conducted unsupervised clustering analysis focused on neutrophils, identifying five subclusters (Figure 4A,B). Comparisons between groups revealed markedly increased proportions of neutrophils in the GA group. The proportion of neutrophils was 1.96‐fold higher among total cells and 1.61‐fold higher among immune cells with GA treatment compared to the control group. (Figure 4C,D). Ly6G immunostaining of neutrophil cells showed a 183.8% increase in GA treated mice, with significantly escalated neutrophil cell migration into the calvarial defect region (Figure 4E). Neutrophil development was characterized using multiple metrics, including CytoTRACE, pseudotime, and module scores of immature and Mature neutrophils [25] (Figure 4F). The confluence of these analyses identified Cluster 0 as immature neutrophils, cluster 1 as cells in intermediate differentiation states, and clusters 2–4 as mature neutrophils. No significant differences were found in the subcluster frequency was identified between treatment groups (Figure 4G). Feature plot show showed primarily no expression of TrkA in neutrophils (Figure 4H; Figure S3B), suggesting that any frequency or phenotypic changes were indirect effects of GA treatment. Next, characterization of differences between GA and control‐treated calvarial defects was performed (Figure 4I–L). The volcano plot illustrates the distribution of DEGs across the entire dataset, of a total of 16 219 genes, with 500 genes (3.1%) of genes significantly overrepresented within the GA group, while 600 genes (3.7%) were overrepresented within the control group (Figure 4I; Table S7). Gene ontology (GO) analyses of upregulated genes among neutrophils in the GA treated mice group revealed significant enrichment in GO terms associated with immune responses, such as cellular response to molecules of bacterial origin, and neutrophil tissue infiltration, such as positive regulation of cell adhesion mediated by integrin [26]. (Figure 4J; Table S8). To assess neutrophil functional status, module scoring was performed using published gene lists [25]. The analysis revealed that most of the seven modules examined—including phagocytosis, neutrophil activation, NADPH oxidase activity, Ros production, specific granules, and gelatinase granules—were significantly elevated in the GA treatment group compared to the control group (Figure 4K). These findings suggest that GA‐mediated enhancement of bone repair was accompanied by augmented neutrophil functionality, potentially beneficial for bone defect healing, although whether these immune shifts are directly driven by neuronal signals or not remains unclear. Notably, only chemotaxis was significantly upregulated in the GA group, whereas proliferation, apoptosis, and necroptosis showed no significant differences between the two groups (Figure 4L). These data indicate that the relative enrichment of neutrophils in calvarial defects of GA‐treated mice was driven by increased chemotactic recruitment rather than alterations in proliferation or survival. Collectively, these findings demonstrate that GA stimulation of bone repair was accompanied by enhanced neutrophil accumulation and activation within bone defects without affecting differentiation.

**FIGURE 5:**
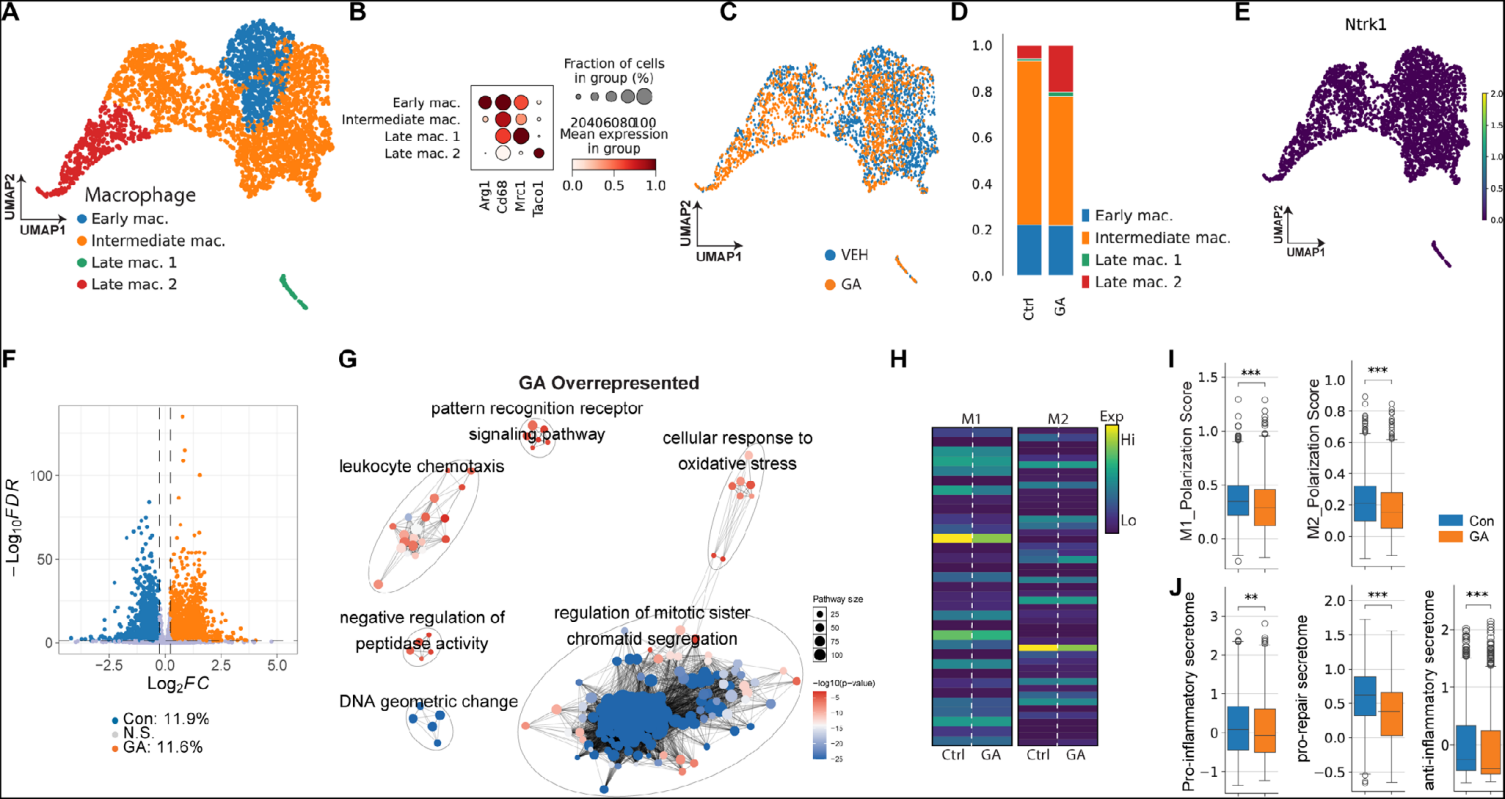
Single‐cell transcriptomic profiling of bone defect‐associated macrophages. C57BL/6J mice were treated with Gambogic Amide (GA) or vehicle control (Con), and frontal bone defects were harvested for scRNA‐Seq at 7 d post‐injury. (A) UMAP plot of Macrophage. (B) Dotplot of marker gene expression. Early macrophages (Early mac.) [arginase 1 (*Arg1*)], Intermediate macrophages (Intermediate mac.) [cluster of differentiation 68 (*Cd68*)], Late macrophage 1 (Late mac. 1) [mannose receptor C‐type 1 (*Mrc1*)], Late macrophage 2 (Late mac. 2) [transient receptor potential cation channel subfamily A member 1 (*Taco1*)]. (C) Distribution of macrophages in the UMAP plot across experimental conditions. (D) Stacked bar plot showing the distribution of cell clusters in Con and GA treated mice. (E) Feature plot illustrating the expression of Ntrk1 projected onto the UMAP. (F) Volcano plot of overrepresented genes in control and GA groups. Significantly differentiated expressed genes are identified using thresholds with FDR < 0.05 and Log2FC > 0.25. (G) GO enrichment map of significantly enriched biological processes (GA Overrepresented GO terms) among all macrophage cells from control‐ and GA‐treated bone defects. (H) Heatmap of marker gene expression of M1 and M2 macrophages among cells derived from control and GA treated mice. (I) Bar plot of M1 and M2 polarization module score. (J) Module score analysis of pro‐inflammatory, pro‐repair, and anti‐inflammatory between GA groups and control macrophage cells. In graphs, each dot represents a single animal. N = 6 mice per group. Data are represented as mean ±1 SD. ^***^ *P*<0.001 as assessed using a two‐tailed Student's *t*‐test.

We next examined changes in macrophages within control and GA treated bone defects. We first performed unsupervised clustering analysis on the macrophage population, identifying four distinct subclusters: early macrophages characterized by high *Arg1* expression, intermediate macrophages characterized by high *Cd68* expression, late macrophages 1 characterized by *Mrc1* (CD206) expression, and late macrophages 2 characterized by *Taco1* expression [27] (Figure 5A,B). Only a minor shift in overall subcluster frequencies was evident (Figure 5C,D). Feature plot showed no expression of TrkA in macrophages, suggesting that any changes in phenotype or polarization are likely indirect effects of GA treatment (Figure 5E; Figure S3B). Next, characterization of differences between GA and control‐treated calvarial defects was performed (Figure 5F–J). Volcano plot illustrates the distribution of DEGs across the entire macrophage dataset, of a total of 18 869 genes, with 2,185 genes (11.6%) of genes significantly were overrepresented under the GA group, while 2254 genes (11.9%) were overrepresented under control groups (Figure 5F; Table S9). GO analyses of upregulated genes in the GA treated mice group were associated with leukocyte chemotaxis and cell response to oxidative stress (Figure 5G; Table S10). The expression of markers linked to both M1 and M2 macrophage activation was significantly reduced in GA treated mice compared to control mice (Figure 5H,I). Early macrophages typically express pro‐inflammatory genes, intermediate ones pro‐repair/pro‐fibrotic genes, and late ones anti‐inflammatory/pro‐osteogenic genes [27]. Module score analysis of published gene lists [27] showed significantly higher scores in control‐treated cells across all modules (Figure 5J). GA treatment led to reduced early pro‐inflammatory and intermediate pro‐fibrotic module scores in post‐fracture macrophages, indicating suppression of inflammation‐to‐fibrosis progression. In summary, GA treatment was associated with a suppression of pro‐fibrotic gene expression profiles in bone defect‐associated macrophages, suggesting that changes in macrophage phenotype may partially contribute to pro‐bone repair outcomes.

### 2.4 Activation of TrkA+ Nerves Promotes Calvarial Defect Healing via Hedgehog Signaling Stimulation

**FIGURE 6:**
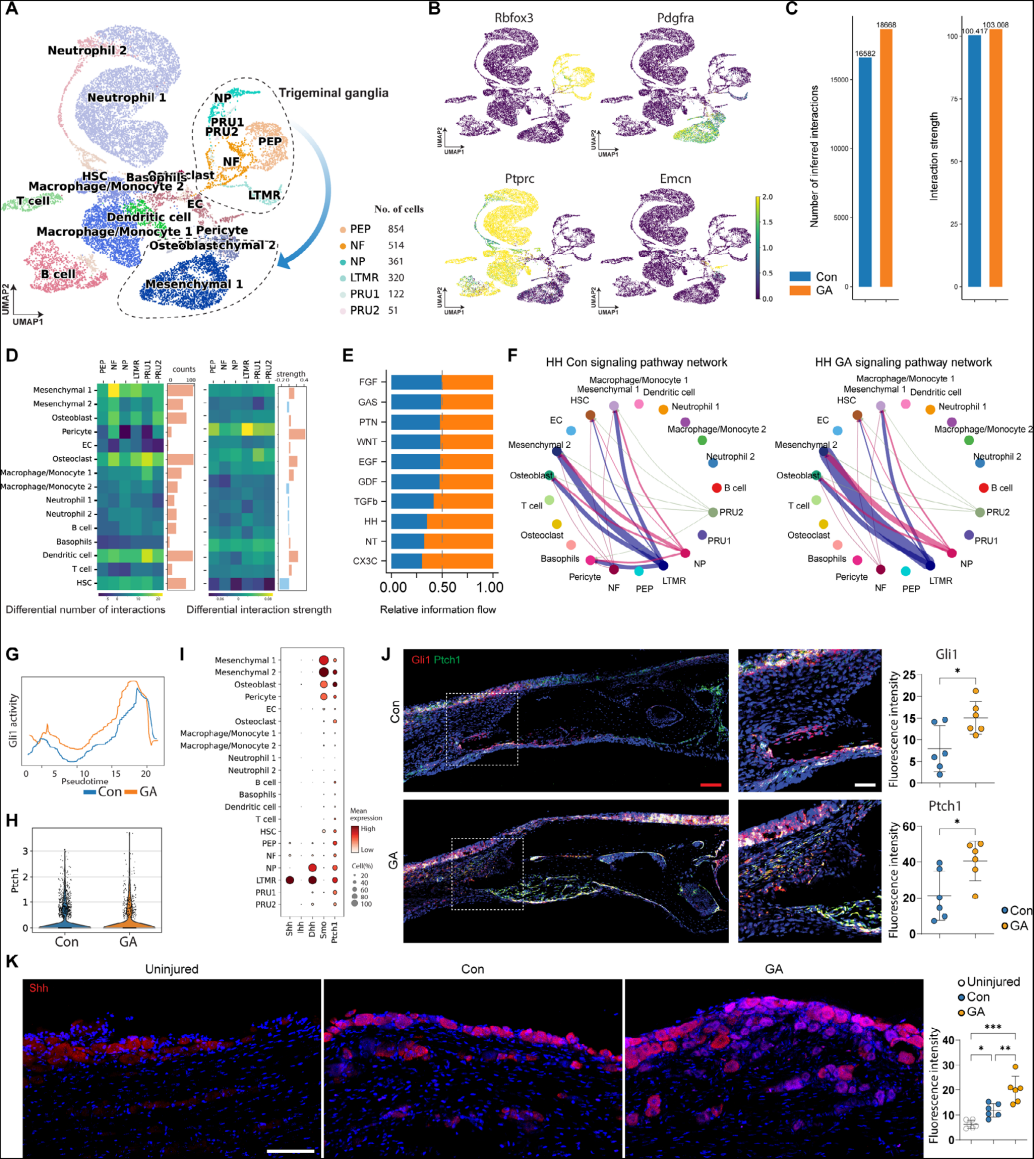
Single‐cell transcriptomic profiling and interactome analysis implicates Hedgehog signaling in neuro‐calvarial signaling interactions. (A) UMAP plots of each cell cluster from the merged database, including all calvarial and neuronal cells. (B) UMAP of key marker gene expression. (C) Bar plot of the number and strength of inferred interactions. (D) Heatmap of interactions of neurons to each calvarial cluster comparing GA with control. (E) Stacked bar plot of the top 10 relative information flow comparing the GA treated with Control. (F) Hierarchy plot compared the HH signaling pathway between Control and GA treatment groups. (G) Line plots comparing Gli1 TF activity between control and GA treatment groups along pseudotime. (H) Violin spot of *Ptch1* expression comparing the GA treatment group with Control. (I) Dot plot of the expression of *Shh, Ihh, Dhh, Smo*, and *Ptch1* in all clusters. (J) Immunohistochemical staining and semi‐quantitative analysis of GLI family zinc finger 1 (GLI1) and Patched 1 (PTCH1) within the calvarial defect, d7 post‐injury. (K) Immunohistochemistry for SHH within trigeminal ganglion sections of uninjured animals as well as those with calvarial injury treated with either vehicle control or GA (7 d post‐injury). Dashed white lines indicate the bone edge. In graphs, each dot represents a single animal. Red scale bar: 100 µm, White scale bar: 50 µm. N = 6 mice per group. Data are represented as mean ±1 SD. ^*^ *P*<0.05, assessed using a two‐tailed Student's *t*‐test.

To explore the mechanisms underlying TrkA agonism stimulation of bone defect repair, particularly the role of neuron cells, we investigated the interactions between neuron cells and local cell populations. Published single‐cell RNA sequencing data (Gene Expression Omnibus (GEO) accession number (GSE213105) of normal mouse trigeminal ganglia was utilized [28]. Single cell RNA‐seq data underwent standard analysis workflows, including filtering, clustering, and retaining only neurons (Figure S2), which were merged with our data for unsupervised clustering analysis. This identified 6 neuronal cell clusters: peptidergic neurons (PEP), non‐peptidergic neurons (NP), large‐diameter myelinated neurons (NF), low threshold mechanoreceptive unmyelinated neurons (LTMR), pruriceptive (itch‐sensing) subpopulations (PRU1), and (PRU2) neuron cell clusters (Figure 6A). Key gene expression, including neuron cell marker Rbfox3, mesenchymal cell marker Pdgfra, immune cell marker Ptprc, and endothelial cell marker Emcn revealed the distributions of neuron cells, mesenchymal cells, immune cells, and endothelial cells in the merged trigeminal ganglia / calvarial defect dataset (Figure 6B). Next, interactome analyses were performed between cells from each tissue. Predictive CellChat cell‐cell communication analysis showed that in comparison to control, GA treatment markedly increased both the number of interactions and interaction strength among different cell populations, indicating enhanced intercellular communication across cell clusters with TrkA agonism (Figure 6C). With neurons designated as the sender cell source, the number of interactions increased for all clusters. Moreover, interaction strength was markedly enhanced for mesenchymal, osteoblast, pericyte, endothelial cells (EC), and other clusters (Figure 6D). Comparing the information flow of each signaling pathway from neurons to mesenchymal cells, the relative information flow revealed enhanced signaling pathways with GA including Fibroblast growth factor signaling pathway (FGF), Growth Arrest‐Specific signaling pathway (GAS), Pleiotrophin signaling pathway (PTN), Wnt signaling pathway (WNT), Epidermal growth factor signaling pathway (EGF), Growth Differentiation Factor signaling pathway (GDF), Transforming growth factor‐beta signaling pathway (TGFb), Hedgehog signaling pathway (HH), Neurotrophin signaling pathway (NT), and CX3C chemokine signaling pathway (CX3C) (Figure 6E). Examining each enhanced pathway, only the Hh signaling pathway was primarily predicted to be secreted by trigeminal neuron cells, with a noticeable increase in GA vs. control, mainly from LTMR and NP neuron subclusters (Figure S4; Figure 6F). In order to further investigate this, pseudotime analysis showed increased Gli1 activity (the main TF in the Hedgehog signaling pathway) with GA treatment compared to control (Figure 6G). The expression of the gene marker of Hh signaling activity Ptch1 was also markedly higher in the GA treatment group in comparison to the control (Figure 6H). Analysis of the expression levels of Hh signaling pathway ligands and key molecules revealed that while mesenchymal cells and osteoblasts exhibited modest expression, neural cells displayed significantly higher expression levels. Notably, *Smo* showed an inverse pattern, with higher expression in mesenchymal cells and osteoblasts. (Figure 6I). GLI1 and PTCH1 immunostaining within calvarial injury sites showed an 89.9% increase in GLI1 and a 91.8% increase in PTCH1 in GA treated mice compared to the control at 7 days post‐injury (Figure 6J). Next, sections of the trigeminal ganglia were examined 7 days after injury among control‐treated and GA‐treated mice. Immunohistochemical staining for Sonic hedgehog (SHH) revealed that SHH fluorescence intensity was markedly increased following calvarial bone injury, with GA treatment further enhancing SHH expression compared to control‐treated mice. These results suggest that Shh signaling is upregulated in response to calvarial bone injury, and that GA treatment amplifies this response (Figure 6K). Furthermore, clear co‐localization of SHH with TUBB3^+^ peripheral nerve fibers was observed within the calvarial defect site (Figure S5A). To further confirm that SHH upregulation is a conserved response to skeletal injury, we analyzed parallel data from an ulna stress fracture model [24], which also heals predominantly via intramembranous ossification. In this distinct injury context, scRNA‐seq of innervating neurons within the dorsal root ganglia (DRG) revealed that Shh expression markedly peaked at day 14 post‐injury (Figure S5B), and SHH protein levels were likewise significantly elevated within the DRG (Figure S5C). The high degree of consistency across different skeletal injury models strongly substantiates the role of sensory neuron‐derived SHH as a key regulator of bone repair.

### 2.5 Smoothened Knockout in Mesenchymal Cells Abrogates the Effects of TrkA Agonism on Calvarial Bone Defect Repair

**FIGURE 7:**
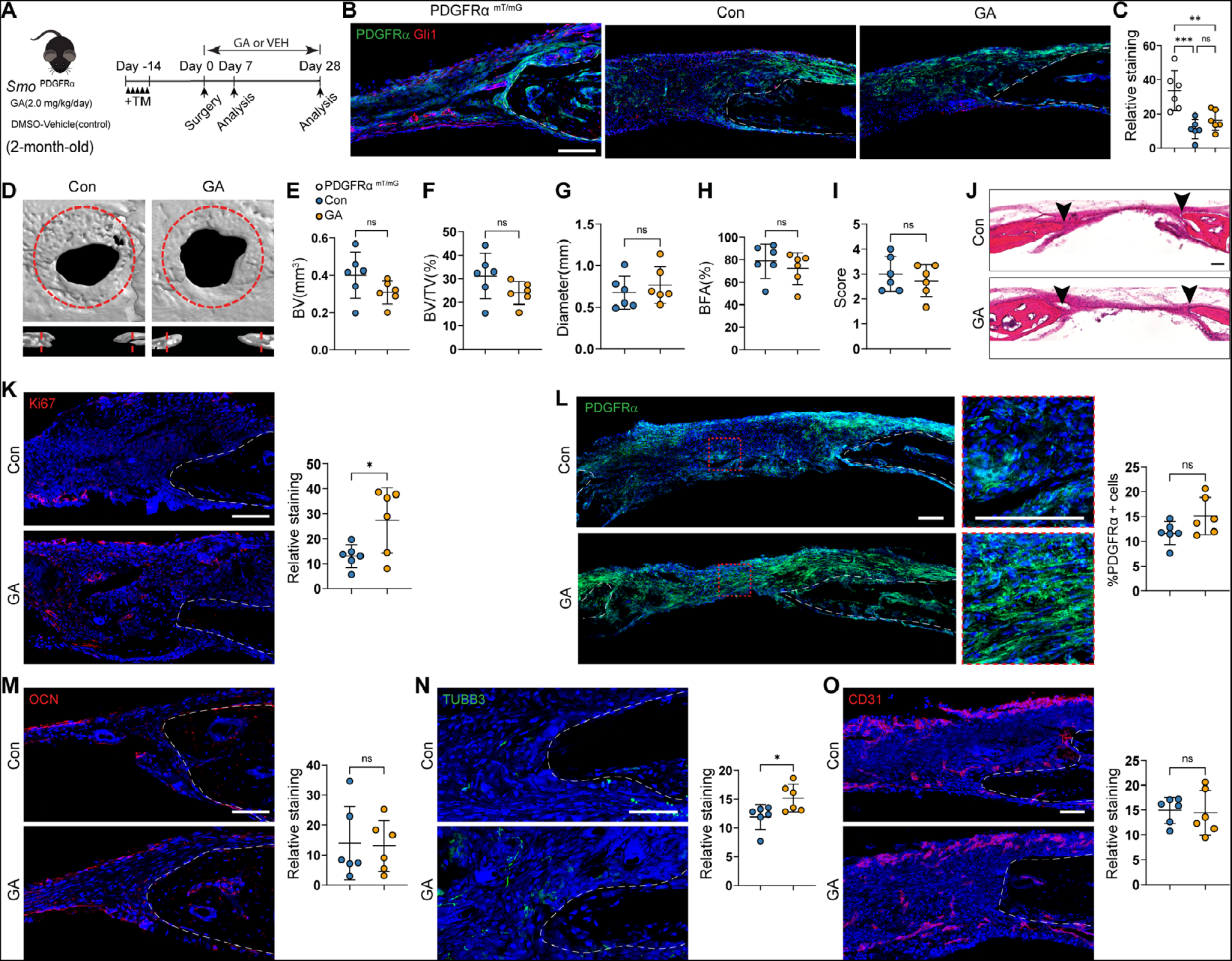
Genetic knockout of Smo abolishes the osteogenic effect of TrkA agonism in calvarial bone defect repair. (A) Schematic of experiment, in which *Smo* ^PDGFRα^ animals (male, 2 mo old) were administered gambogic amide (GA) or DMSO‐vehicle, followed by frontal bone injury (circular calvarial defect, 1.8 mm diameter), with analysis performed 7 or 28 d post‐operatively. (B) Immunohistochemistry for GLI1 (appearing red) within coronal cross‐section of the healing defect site of *Pdgfr* *a* ^mT/mG^ animals as well as those treated with either vehicle control or GA (7 d post‐injury). (C) Semi‐quantitative analysis of GLI1 7 d post‐injury. (D) Micro‐CT reconstructions of the defect site in a top‐down view (above) and coronal cross‐sectional images (below) among Control and GA treated animals, 28 d post‐injury. Margins of the original defect are indicated by dashed red lines. (E–I) Micro‐CT quantification of bone healing among Control‐ and GA‐treated mice (28 d post‐injury), including (E) Bone volume (BV), (F) Bone volume/tissue volume (BV/TV), (G) residual defect diameter, (H) Bone formation area (BFA), and (I) Bone Healing Score. (J) H&E staining of coronal cross‐section of the healing defect site from control‐ and GA‐treated mice at d28 post‐injury. Black arrowheads indicate healing bone edges. (K) Cellular proliferation at the bone defect edge, as assessed by Ki67 immunofluorescent staining at d7 post‐injury. (L) Mesenchymal cell migration, as assessed by Pdgfrα immunofluorescent staining. Tile scan (left) and high‐magnification images of the central defect (right) demonstrate migration of PDGFRα^+^ progenitor cells into the defect site at d7 post‐injury. (M–O) Immunohistochemical staining and semi‐quantitative analysis of Osteocalcin (OCN) 28 d post‐injury (M), Beta III Tubulin (TUBB3) (N), and CD31 (O) within the calvarial defect, d7 post‐injury. Dashed white lines indicate bone edges. In graphs, each dot represents a single animal. Black scale bar: 500 µm. White scale bar: 100 µm. N = 6 mice per group. Data are represented as mean ±1 SD. ^*^ *P*<0.05, ^**^ *P*<0.01, and ^***^ *P*<0.001 as assessed using a two‐tailed Student's *t*‐test.

To determine whether TrkA agonist‐mediated bone repair is a Hedgehog‐dependent process, experiments with gambogic amide treatment were next replicated in mice lacking the Hh receptor Smo in PDGFRα‐expressing mesenchymal cells (*Smo* ^fl/fl^; PDGFRα‐CreERT2, or *Smo* ^*Pdgfr* *a*^). To confirm the efficiency of deletion, we isolated PDGFRα+ cells from the calvaria using magnetic‐activated cell sorting (MACS). Quantitative PCR analysis of the sorted cells confirmed a robust deletion efficiency, with *Smo* mRNA levels being reduced by 87.6% in the *Pdgfra*+ cells of *Pdgfra*‐*Cre* ^ERT2^; *Smo* ^fl/fl^ mice compared to *Smo* ^fl/fl^ controls (Figure S6). With this validated model, we then evaluated the calvarial bone repair. SmoPdgfrα conditional knockout mice were treated with gambogic amide (GA, 2 mg/kg) or vehicle control following calvarial defect creation, with analyses at 7 and 28 days post‐injury (Figure 7A). Immunohistochemical analysis of GLI1 at day 7 confirmed effective suppression of Hh signaling in SmoPdgfrα mice, as evidenced by diminished GLI1 expression in both vehicle‐ and GA‐treated animals compared to control PdgfrαmT/mG mice (Figure 7B,C). Micro‐CT analysis at day 28 revealed that genetic disruption of Hedgehog signaling completely abolished the bone regenerative effects of TrkA agonism (Figure 7D). No significant differences were observed between GA‐ and vehicle‐treated SmoPdgfrα mice across all quantitative micro‐CT metrics (Figure 7E–I). H&E staining confirmed similar healing in both treatment groups (Figure 7J). Despite no change in bone healing, Ki67 immunostaining revealed a 110.5% increase in proliferation in GA‐treated mice, (Figure 7K, similar to effects previously observed in WT mice). Likewise, GA slightly enhanced the migration of PDGFRα‐positive mesenchymal cells into the defect in SmoPdgfrα mice (Figure 7L). OCN immunohistochemical staining demonstrated no differences in osteoblast differentiation at day 28 between treatment groups in SmoPdgfrα mice (Figure 7M). TUBB3 immunostaining revealed a significant 27.5% increase in nerve fiber density in GA‐treated mice at day 7 (Figure 7N, a lesser effect than previously observed between treatment groups in WT mice). In similarity to WT mice, CD31 immunostaining showed comparable vascular density between groups in SmoPdgfrα mice (Figure 7O). In summary, these results demonstrate that intact Hedgehog signaling is essential for certain aspects of TrkA agonist‐mediated bone regeneration, including osteogenesis and ultimately bone repair outcomes, while injury site proliferation appeared to be Hedgehog independent.

## 3 Discussion

In this study, we activated TrkA signaling experimentally in mice using gambogic amide (GA) and demonstrated enhanced bone healing, including greater nerve ingrowth into the injured bone. Our single‐cell RNA sequencing analysis revealed that TrkA agonism indeed enhanced osteogenesis at the expense of fibrogenesis, and this appears to be at least in part due to activation of downstream Hedgehog signaling.

Gli1 is one of the vital transcription factors as well as the direct target gene in the Hh signaling pathway. Gli1+ cells were first identified as the incisor mesenchyme cells [29]. Subsequently, Gli1 positive cells have been identified as stem‐like cells in craniofacial bones [30]. Gli1 positive cells possess the fundamental characteristics of stem‐like cells, being capable of differentiating into osteogenic bone cells and participating in calvarial repair after injury. In vitro assays have further characterized Gli1 positive cells as possessing clone‐forming ability and the potential to undergo osteogenic, chondrogenic, and adipogenic differentiation [30]. The expression of Gli1 is restricted to cells adjacent to peaks in Hedgehog (Hh) signaling levels [31, 32]. This implicates the importance of Hh signaling for Gli1 positive cells, however the source of Hh ligands remains unclear. In recent years, it has been speculated that peripheral nerves may provide a source of Hh ligands. For instance, Shh‐producing nerves have been shown to regulate mesenchymal progenitor cells in the mouse incisor [29] and epithelial stem cells in the upper bulge of the hair follicle [33]. Robust evidence underscores the pivotal role of Hh signaling in determining cell fate during osteogenesis, with the pathway promoting osteoblast differentiation [34, 35, 36, 37]. To our knowledge, sensory nerve‐released Hh ligands have not previously been implicated as a main cell fate determinant during bone repair. Notably, our validation data demonstrate that the pro‐regenerative SHH response is conserved across diverse skeletal sites (Figure S5). This suggests that boosting neurogenic Hedgehog signaling via sensory neurons may represent a broadly applicable therapeutic strategy for various bone injuries.

Gambogic acid is a major active ingredient of gamboge, a resin exuded from the Garcinia hanburyi tree in Southeast Asia. Gambogic amide, a derivative of gambogic acid synthesized by amidation of its carboxyl group, shares a core caged polycyclic structure with gambogic acid and thus retains some of its biological functions, including anti‐apoptotic activity and neuroprotection [13]. Among gambogic acid derivatives, gambogic amide was identified through screening for its unique ability to selectively bind to the cytoplasmic juxtamembrane domain of TrkA receptors, whereas other derivatives lack this potency. As a small‐molecule agonist, gambogic amide facilitates nerve growth factor (NGF) activity through allosteric activation [13]. It selectively binds, activates, and upregulates the expression of TrkA receptors at nanomolar concentrations but not TrkB or TrkC. While NGF causes transient TrkA activation and degradation, gambogic amide induces lower but more sustained TrkA phosphorylation [38]. From a therapeutic perspective, systemic administration of the TrkA agonist gambogic amide (GA) exhibits a favorable safety profile. Notably, compared to systemic NGF administration, its greatest safety advantage is that it does not induce systemic hyperalgesia [13, 39, 40]. Studies have also demonstrated other physiological functions of gambogic amide, such as anti‐angiogenic properties via the suppression of VEGF/VEGFR2 signaling in a TrkA‐independent manner [41]. Indeed, GA treatment in our model showed VEGF signaling suppression, which may explain the lack of any bone defect‐associated improvement in vascularization, which would otherwise be expected in the context of boosted innervation [42]. However, it should be noted that while GA is well‐tolerated at therapeutic doses, excessively high concentrations may induce cytotoxicity, and its off‐target anti‐angiogenic effects warrant further consideration for chronic systemic applications [41, 43].

It is worthwhile to note that not all effects of TrkA agonism on bone defect repair were observed to be Hedgehog dependent. While GA stimulated re‐ossification was clearly Hedgehog signaling dependent, stimulation of proliferation of cells within the defect site was observed to be Hedgehog independent (a similar enhancement of proliferation was observed in WT and Smo conditional knockout animals). One explanation for this is that bone injury repair depends on multiple peripheral nerve‐derived signals. In recent work, we identified that somatosensory nerve‐derived fibroblast growth factor 9 (FGF9) stimulated osteoprogenitor cell proliferation during long bone fracture repair [24]. It is plausible that FGF9 or other mitogenic signals function in combination with nerve‐derived SHH to positively regulate bone tissue repair.

NGF itself has several limitations for clinical translation, including poor pharmacokinetic properties and pain‐evoking effects. Specifically, NGF is susceptible to proteolytic degradation and has a short elimination half‐life following systemic administration [44, 45, 46]. Moreover, NGF evokes pain on application, which severely limits its clinical utility [47], while the specific TrkA agonist Gambogic amide (GA) does not produce thermal or mechanical hyperalgesia [48]. GA was first identified as a TrkA partial agonist with the use of a cell‐based chemical genetic screen [13]. GA is a non‐peptide small molecule that has been shown to selectively bind to the juxtamembrane domain of TrkA with high affinity, inducing TrkA phosphorylation, dimerization, and downstream signaling activation, including Akt and MAPK signaling [38]. GA has been used experimentally in mouse and rat models to prevent neuronal apoptosis, mitigate infarct size in stroke [13], and, most recently, speed fracture healing in bone [12]. In addition, it is well tolerated in vivo across small animal models.

A limitation of this study is the reliance on systemic GA administration, which results in TrkA activation in a non‐localized fashion and potentially generalized sensory nerve activation. Consequently, localized GA administration represents an ideal strategy to bypass these systemic side effects. Since there are currently no published reports on the local application of GA, our future studies will focus on determining the optimal effective dose and biomaterial strategies for targeted local delivery.

In summary, this study expands our understanding of the signaling pathways underlying peripheral afferent nerve regulation of bone repair, suggesting a role for peripheral neuron‐derived Hedgehog signaling in directing cell fate decisions toward osteoblastic differentiation to promote tissue healing.

## 4 Experimental Section/Methods

### 4.1 Mice

All animal experiments were performed according to the approved protocols (MO22M368) of the Animal Care and Use Committee at Johns Hopkins University and conducted in accordance with ARRIVE guidelines. All animals were housed in IACUC‐supervised facilities at 18°C–22°C, 50% (±20%) of relative humidity, and 12‐h light–dark cycle with ad libitum access to food and water. Wild‐type C57BL/6J mice were purchased from the Jackson Laboratory (JAX) (Bar Harbor, ME, USA). *Thy1*‐YFP mice, which harbor a transgene derived from the mouse *Thy1* gene that directs expression of YFP in neurons [49], are commercially available [the Jackson Laboratory (JAX), stock no. 003709 Bar Harbor, ME, USA]. Mice with *Smo* floxed alleles (JAX: 004526) were donated from the Reeves laboratory and crossed with Pdgfra^mT/mG^ mice (JAX: 018280, JAX: 007576) donated from the Bergles laboratory.

Tamoxifen (TM, Sigma–Aldrich, St. Louis, MO, USA) was dissolved in sunflower seed oil (Sigma–Aldrich) and injected intraperitoneally according to the previously validated protocols (TM, 150 mg kg^−1^ day^−1^ for 5 days) [14]. Gambogic amide (GA) (Enzo Life Sciences, Farmingdale, NY), a non‐peptide small molecule that selectively binds to the juxtamembrane domain of TrkA, was used to activate TrkA [13]. Mice received intraperitoneal (IP) injection of either 100 µL vehicle control (10% DMSO) or GA (2 mg/kg in 10% DMSO). Treatment began 24h prior to calvarial injury, and continued daily thereafter for 4 wks.

### 4.2 Validation of Conditional *Smo* Deletion via MACS and qPCR

To quantify the *Smo* deletion efficiency, *Pdgfr‐Cre^ERT2^; Smo^fl/fl^* and control *Smo^fl/fl^* mice (n = 3/group) received intraperitoneal injections of tamoxifen (100 mg/kg/day) for 5 consecutive days. One week after the final injection, calvarial tissues were harvested and enzymatically digested to obtain single‐cell suspensions. PDGFRα+ mesenchymal cells were then enriched using Magnetic‐Activated Cell Sorting (MACS). Briefly, the cell suspension was incubated with a Biotin anti‐mouse CD140a (PDGFRα) antibody (BioLegend, #135909), followed by incubation with MojoSort Streptavidin Nanobeads (BioLegend, #480015). The labeled PDGFRα+ cells were separated utilizing a MojoSort Magnet. Total RNA was extracted from the sorted PDGFRα^+^ cells using TRIzol Reagent (Invitrogen, Carlsbad, CA, USA) according to the manufacturer's instructions. 0.2 µg of total RNA was reverse‐transcribed into cDNA using iScript cDNA synthesis kit (Bio‐Rad, Hercules, CA) following the manufacturer's instructions. Real‐time PCR was performed using SYBR Green PCR Master Mix (ThermoFisher Scientific, Waltham, MA) according to the manufacturer's protocol to evaluate the mRNA expression levels of *Smo*. Relative gene expression was calculated using the 2^−ΔΔCt^ method with normalization to *Gapdh*. The specific primer sequences used for *Smo* amplification were: forward, 5'‐CCCTGCTGTGTGCTGTCTAC‐3'; and reverse, 5'‐GTGTGCAACGCAGAAAGTCAG‐3'.

### 4.3 Calvarial Defect Procedures

Calvarial defects were performed on the basis of our prior methods [14]. Mice were anesthetized with isoflurane (2%–3% inhaled) and treated with buprenorphine (1mg/kg, subcutaneously) for pain management. Briefly, hair overlying the calvaria was clipped, and a 1‐cm skin incision was made over the midline skull to expose the frontal bone. Next, a 1.8‐mm‐diameter, full‐thickness, circular frontal bone defect was created in the non‐suture‐associated frontal bone (right side) using a microsurgical drill and a trephine drill bit. Meticulous care was taken to protect the neighboring sutures and the underlying dura mater. Last, the skin was sutured, and the animals were monitored per established postoperative protocols. Mice were humanely euthanized after 7 or 28 d for analysis.

### 4.4 Radiographic Analyses

Skulls were fixed in 4% paraformaldehyde (PFA) for 24 h and evaluated using a high‐resolution micro‐CT imaging system (SkyScan 1275; Bruker, Kontich, Belgium). Scans were obtained at an image resolution of 12 µm and set as 1 mm of aluminum filter, x‐ray voltage of 65 kVp, anode current of 153 µA, exposure time of 218 ms, frame averaging of 2, and rotation step of 0.3°. Three‐dimensional (3D) images were then reconstructed from the 2D x‐ray projections using the commercial software NRecon software (v1.7.0.4, SkyScan). For 3D morphometric analyses of images, CTAn software (v1.16, SkyScan), CTVol (v2.0, SkyScan), and CTVox (v3.2, SkyScan) were used. For calvarial defect analysis, a cylindrical volume of interest centered around each defect site was defined as the 1.8 mm in diameter and 1.2 mm in height with a threshold value of 70–255. BV and fractional BV (BV/TV) were calculated from binary x‐ray images. BFA and defect diameter were calculated by using CTVox to create a 3D rendering of the calvarial defect and measuring by ImageJ software (version 1.5.2; National Institutes of Health, Bethesda, MD, USA). Lastly, a semi‐quantitative bone healing score from 0 to 4 was assigned by 3 blinded observers according to previous published grading scales for calvarial defect healing [50]. Briefly, the grading system was as follows: 0–no bone formation, 1–few bony spicules dispersed through defect, 2–bony bridging only at defect borders, 3–bony bridging over the partial length of the defect, and 4–bony bridging the entire span of the defect at the longest point.

### 4.5 Histology and Immunohistochemistry

Calvaria were harvested and placed in 4% PFA at 4°C for 24 h. After sequential washes in PBS x 3, samples were decalcified in 14% EDTA (1:20 volume, Sigma–Aldrich) for 14 d at 4°C. Coronal sections of the calvaria were obtained using cryosections at 20 or 50 µm thickness. Trigeminal ganglion were harvested and placed in 4% PFA at 4°C for 24 h. Coronal sections of the trigeminal ganglion were obtained using cryosections at 20 µm thickness. For cryosections, samples were cryoprotected in 30% sucrose overnight at 4°C before embedding in OCT (Tissue‐Tek 4583, Torrance, CA). Coronal sections were mounted on adhesive slides. For immunohistochemistry, sections were washed in PBS x 3 for 10 min. Sections were next permeabilized with 0.5% Triton‐X for 15–30 min. Next, 5% normal goat or donkey serum was applied for 30 min, then incubated in primary antibodies overnight at 4°C in a humidified chamber (see Table S1 for a summary of antibodies used). The following day, slides were washed in PBS, incubated in the appropriate secondary antibody for 1 h at 25°C, then mounted with DAPI mounting solution (VECTASHIELD PLUS Antifade Mounting Medium, Vector Laboratories, Burlingame, CA). Digital images of these sections were captured with 10–20 × objectives using upright fluorescent microscopy (Leica DM6, Leica Microsystems Inc., Buffalo Grove, IL) or confocal microscopy (Zeiss LSM980 FCS, Carl Zeiss Microscopy GmbH, Jena, Germany).

### 4.6 Single‐Cell RNA Sequencing Preparation

For single‐cell RNA sequencing of the calvarial defect model, cells were harvested and pooled from n = 5 biological replicates (mice) per group (Control vs. GA‐treated) to ensure adequate biological representation and to minimize individual variation.

Skulls were microdissected 7 d after defect creation. A circular segment of cranial bone, 3mm in diameter with the defect at the center, was minced and subjected to six sequential enzymatic digestions with a mixture containing collagenase type I (1 mg/mL; Worthington Biochemical Corporation, Lakewood, NJ, USA, LS004197) and collagenase type II (1 mg/mL; Worthington Biochemical Corporation, LS004177). Cell fractions (from sequential digestions 1–6) were collected and resuspended in red blood cell lysis buffer (37°C for 5 min). Digestions were subsequently filtered through 40‐µm sterile strainers. Cells were then washed in PBS and resuspended in HBSS at a concentration of ∼1000 cells/µL. Cell viability was assessed with Trypan blue exclusion on a Countess II (Thermo Fisher Scientific) automated counter and showed >85% viability. Cells were pooled for analysis and were sent to the JHMI Transcriptomics and Deep Sequencing Core. Library construction was performed using the Chromium GEM Single Cell 3′ Reagents kit v3.1 (10x Genomics) in accordance with the manufacturer's protocol. Sequencing was performed on a Novaseq 6000 (Illumina).

### 4.7 Processing and Analysis of scRNA‐seq Data

Single cell transcriptomes were obtained using Chromium GEM‐X Single Cell Gene Expression (3’ v4) assay. Raw data were processed using the Cell Ranger software suite v7.1.0 with reference genome mm10‐2020‐A, and doublets were removed using Scrublet v0.2.1. Downstream analysis steps were performed using Scanpy v1.9.8 [51]. We excluded cells with fewer than 500 unique molecular identifiers (UMIs), or more than 140 000 UMIs. Genes expressed in less than 3 cells were filtered out. Lastly, we removed cells expressing less than 200 unique genes or more than 9500 genes and cells with a mitochondrial gene content higher than 15%, which resulted in 105 and 539 cells. Then, we used Pearson residuals normalization, as implemented in Scanpy v1.9.8, and PCA transformation of normalized counts. Batch effects were corrected via Harmony [52]. Dendrograms for cell types were constructed using PCA embeddings with the complete linkage method. For the cell type annotation, neighborhood graphs were constructed using Euclidean distances on 50 PCs with 15 neighbors. We then applied Leiden clustering, which we visualized using a UMAP embedding. DEGs between clusters were identified by aggregating single‐cell counts into pseudobulk profiles per cluster and treatment group, followed by differential expression analysis using PyDESeq2 with default parameters [53]. Module scores were calculated using scanpy.tl.score_genes (Scanpy v1.9.8) with default parameters, representing enrichment of gene set expression above background.

Output BAM files were further processed by Velocyto v0.17.15 to generate LOOM files containing UMI of spliced RNA, unspliced RNA, and ambiguous as separate matrices with the default parameters for downstream and RNA velocity analysis. RNA velocity analysis was done by scVelo v0.2.5 with dynamic modeling. The calculated RNA velocity vectors were projected onto the pre‐computed UMAP embedding. Pseudotime trajectories of osteogenesis and fibrogenesis were constructed using the R package Monocle 2 v2.24.0. We collected and sorted differentially expressed genes (DEGs, top 500) in all clusters. Subsequently, the expression profile was reduced to two dimensions using the DDRTree algorithm, and the cells were sorted using these 500 DEGs to obtain a trajectory map. Pseudotime values were added in Anndata object as metadata. To assess the level of differentiation of the cell clusters, we performed additional analysis using CytoTrace with the default parameters [54]. The calculated CytoTRACE score was then embedded with UMAP into a lower‐dimensional graph. GO enrichment and KEGG of DEGs was performed using the R package clusterProfiler, with a Benjamini–Hochberg (BH) multiple testing adjustment and a false‐discovery rate (FDR) cutoff of 0.1. aPEAR was used to objectively summarize the enrichment results by using the similarity between pathway gene sets and representing them as interconnected cluster networks [55]. Single‐cell regulatory network inference and clustering (SCENIC) was used to infer active transcription factor (TF) networks. Analysis was performed using recommended parameters using the packages pySCENIC v0.12.1 and the motif databases RcisTarget and GRNboost. To integrate public trigeminal ganglion (TG) data (GSE213105) with our calvarial dataset, neuronal cells were subsetted from the public data and merged with our annotated experimental dataset using the anndata.concat function (parameters: join = ‘outer’, merge = ‘unique’). The integrated count matrix was then processed for batch correction (Harmony) and downstream visualization (UMAP and Leiden clustering) following the same computational pipeline as described above. This integrated dataset served as the foundation for subsequent neuron–bone interactome analysis. Cell communication analysis was performed using the R package CellChat v2.1.0 with default parameters on the complete combined dataset.

### 4.8 Statistical Information

Data are presented as mean ± 1 SD. Statistical analyses were performed using GraphPad Prism (version 10.6.0) or python package statannotations or R package ggpubr. The number of animals used in in vivo experiments is shown in the figure legends. For these scenarios, with at least six mice per group, a two‐sample *t* test would provide 80% power to detect effect sizes of at least 1.75, assuming a two‐sided 0.05 level of significance. The Kolmogorov‐Smirnov test was used to confirm the normal distribution of the data. A two‐tailed Student's *t* test or Wilcoxon test was used for two‐group comparisons. A one‐way analysis of variance (ANOVA) test was used for multiple groups, followed by Tukey's multiple comparisons test. ^*^ *P* < 0.05, ^**^ *P* < 0.01, and ^***^ *P* < 0.001 were defined as statistically significant.

## Funding

NIH/NIAMS R01AR079171 (AWJ), NIH/NIAMS R21AR078919 (AWJ), NIH/NIDCR R01DE031488 (AWJ), NIH/NIDCR R01DE031028 (AWJ), the Alex's Lemonade Stand Foundation 22–26743 (AWJ), the American Cancer Society DBG‐23‐1155131‐01‐IBCD (AWJ), the Maryland Stem Cell Research Foundation 2021‐MSCRFD‐5641 (AWJ), the Department of Defense USAMRAA HT9425‐24‐1‐0051 (AWJ).

## Conflicts of Interest

A.W.J. declared scientific advisory board member for Novadip LLC, consultant for Lifesprout LLC and Novadip LLC, and Editorial Board of Bone Research, Stem Cells, and The American Journal of Pathology. All the other authors declared no potential conflicts of interest.

## Supporting information




**Supporting File**: advs75389‐sup‐0001‐SuppMat.docx.

## Data Availability

The data that support the findings of this study are openly available in NCBI GEO at https://www.ncbi.nlm.nih.gov/geo/, reference number GSE266598.
