# Supplementary material for: Boosting Sensory Nerve‐to‐Bone Interactions Enhances Hedgehog Mediated Calvarial Bone Repair
Source: Adv Sci (Weinh). 2026 Apr 20;13(40):e75389. doi: 10.1002/advs.75389 (PMC13335536; doi:10.1002/advs.75389)
Supplement: Supplementary file 1 — Supporting File: advs75389‐sup‐0001‐SuppMat.docx. [file ADVS-13-e75389-s001.docx]

Supporting Information

Boosting Sensory Nerve-to-Bone Interactions Enhances Hedgehog Mediated Calvarial Bone Repair

*Zhao Li, Xin Xing, Beicheng Du, Myles Zhou, Austin Z. Chen, Mary Archer, Chunbao Rao, Manyu Zhu, Masnsen Cherief, Aaron W. James**

**Supporting Information**

**Figure S1.**


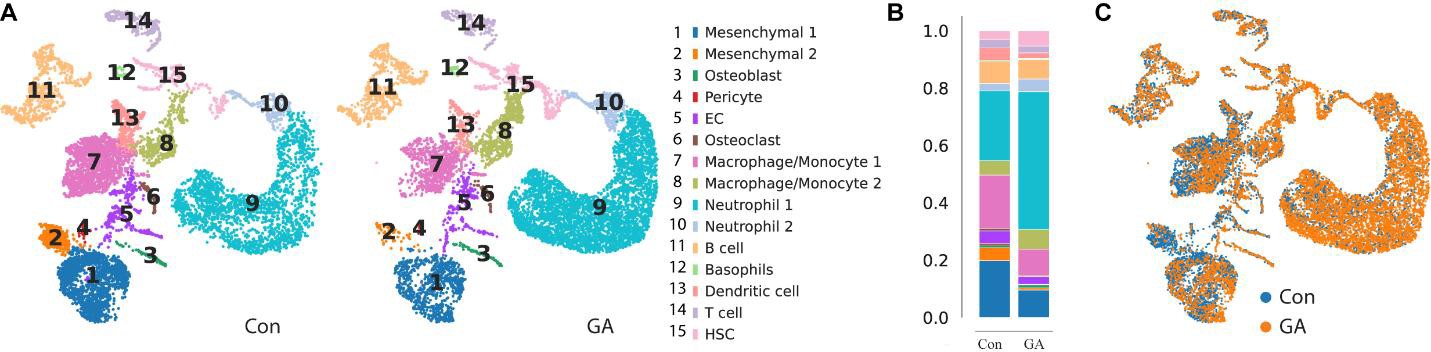


**Figure S1. Distribution of cell clusters among treatment groups mice.** C57BL/6J mice were treated with Gambogic Amide (GA) or vehicle control (Con), and frontal bone defects harvested for scRNA-Seq at 7 d post-injury. (A) Distribution of 16 cell clusters in UMAP plots from Con and GA treated mice. (B) Stacked bar plot showing the distribution of cell clusters in Con and GA treated mice. (C) Merged UMAP of cells derived Con and GA treated mice. N=17,133 total cells derived from C57BL/6J mice.

**Figure S2.**


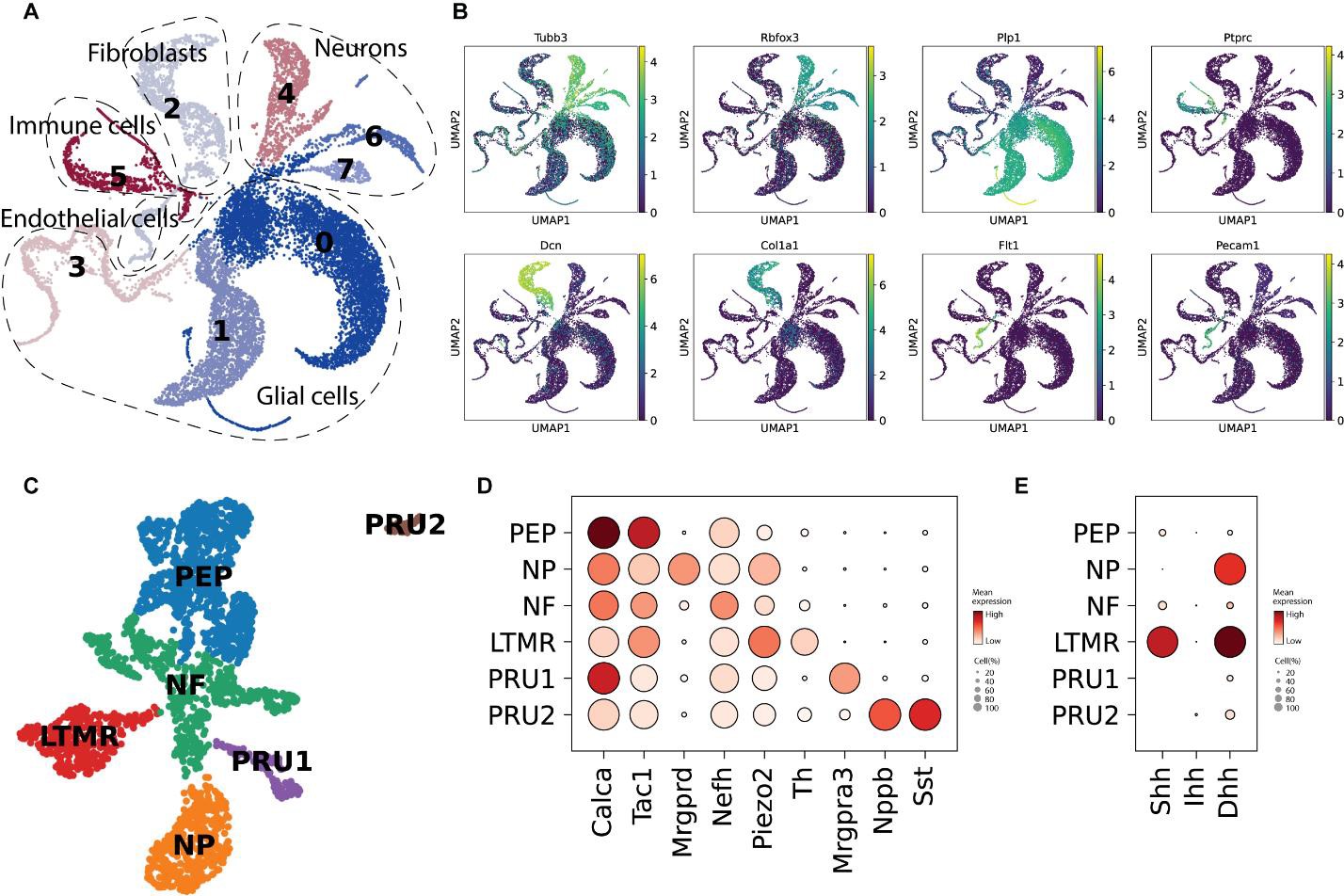


**Figure S2. Identification of cell clusters and neuron cell types in mouse trigeminal ganglion**. **A** UMAP projection of cell clusters and cell types. **B** UMAP of key marker gene expression, including neuronal (*Tubb3, Rbfox3*), glial (*Plp1*), immune (*Ptprc*), fibroblastic (*Dcn, Col1a1*), and endothelial markers (*Flt1, Pecam1*). **C** UMAP projection of neuronal subclusters. Six neuronal subpopulations including peptidergic neurons (PEP), non-peptidergic neurons (NP), large-diameter myelinated neurons (NF), low threshold mechanoreceptive unmyelinated neurons (LTMR), and pruriceptive neurons types 1 and 2 (PRU1, PRU2) were identified. **D** Dot plot of known marker genes for each neuronal subcluster. **E** Dot plot of Hedgehog pathway ligands for each neuronal subcluster. N=14,517 total cells and N=2,222 neurons analyzed.

**Figure S3.**


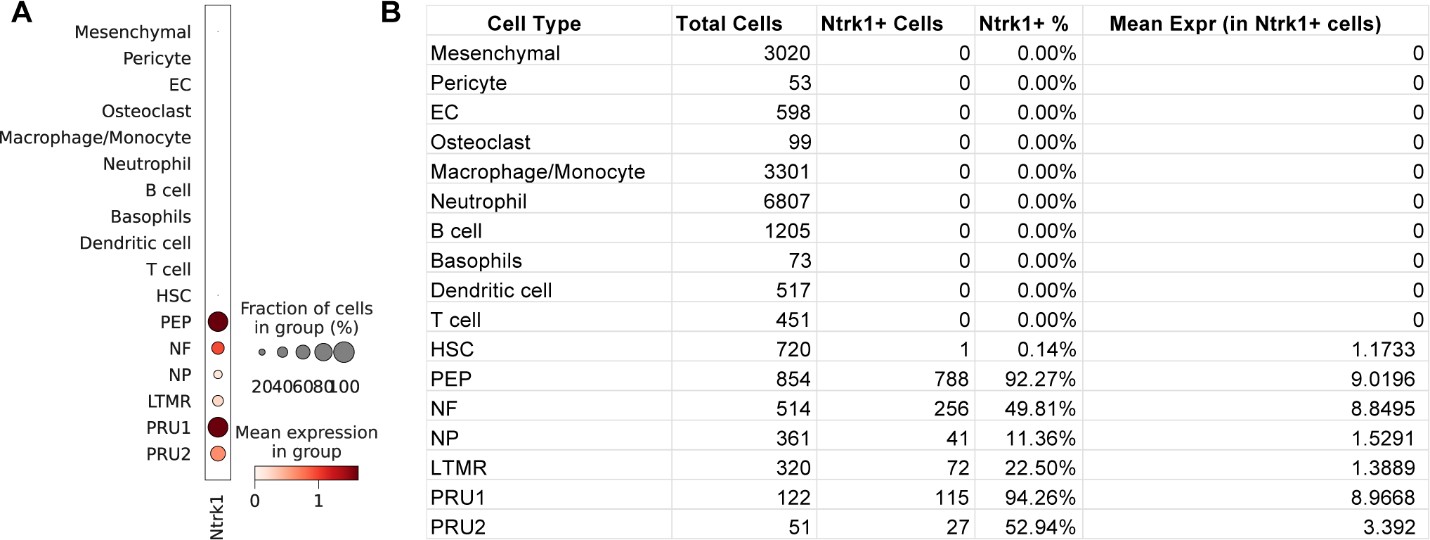


**Figure S3. Expression of *Ntrk1* in all clusters**. **A** Dot plot of the expression of *Ntrk1* in cells of the calvarial bone defect and neurons of the trigeminal ganglia. EC: Endothelial cell; HSC: hematopoietic stem cell; PEP: peptidergic neurons; NF: large-diameter myelinated neurons; NP: non-peptidergic neurons; LTMR: low threshold mechanoreceptive unmyelinated neurons; PRU1: pruriceptive (itch-sensing) subpopulations 1; PRU2: pruriceptive (itch-sensing) subpopulations

**B** Table of *Ntrk1* expression in all clusters the calvarial bone defect and neurons of the trigeminal ganglia.

**Figure S4.**


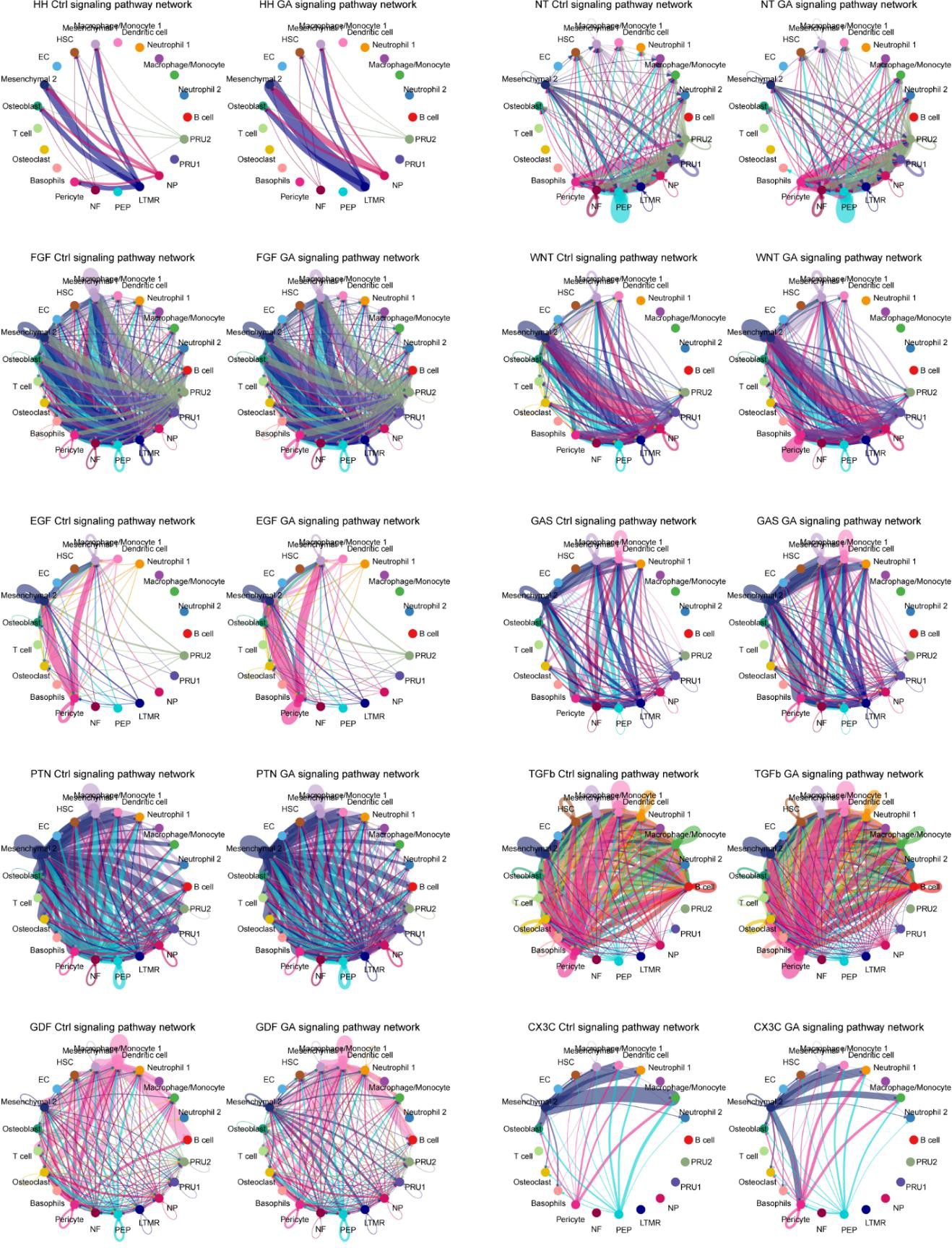


**Figure S4. Hierarchy plot compared different signaling pathway between Control and GA.** Edge colors are consistent with the sources as sender, and edge weights are proportional to the interaction strength. Thicker edge line indicates a stronger signal.

**Figure S5.**


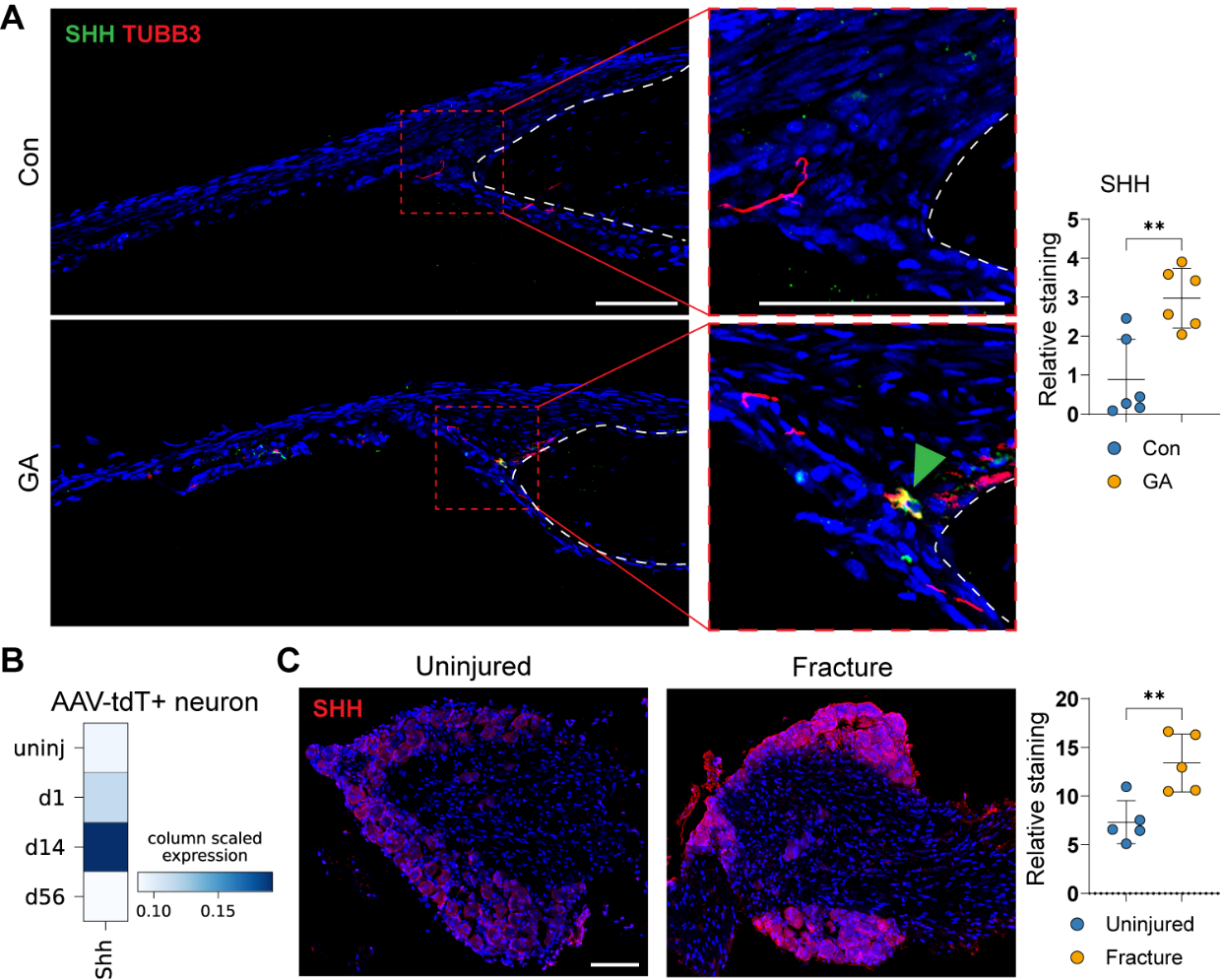


**Figure S5. Skeletal injury upregulates SHH expression in sensory neurons, which is further enhanced by gambogic amide (GA) treatment.** (A) Representative immunofluorescence (IF) images showing co-staining of SHH (green) and the pan-neuronal marker beta-III Tubulin (TUBB3, red) in the calvarial defect of control (Con) and experimental (GA) groups. Quantification of SHH relative staining intensity is shown on the right (n = 6 mice per group). Dashed white lines indicate the bone margins. The green arrowhead highlights the co-localization of SHH and TUBB3. (B) Heatmap (matrix plot) illustrating the temporal expression profile of *Shh* in skeletal-innervating (AAV-tdT+) neurons at multiple time points (uninjured, day 1, day 14, and day 56) post-injury, showing peak expression at day 14. Data adapted from published work^[24]^. (C) Immunohistochemical (IHC) staining for SHH within Dorsal Root Ganglion (DRG) sections from uninjured mice and mice 14 days post-ulna stress fracture. New staining generated from published work^[24]^. Corresponding quantification of SHH relative staining is provided (n = 5 C57BL/6J mice per group). Scale bars: 100 μm. Data are presented as mean ± SD. ***P* < 0.01 using a two-tailed Student’s t-test. Each dot represents an individual biological replicate.

**Figure S6.**


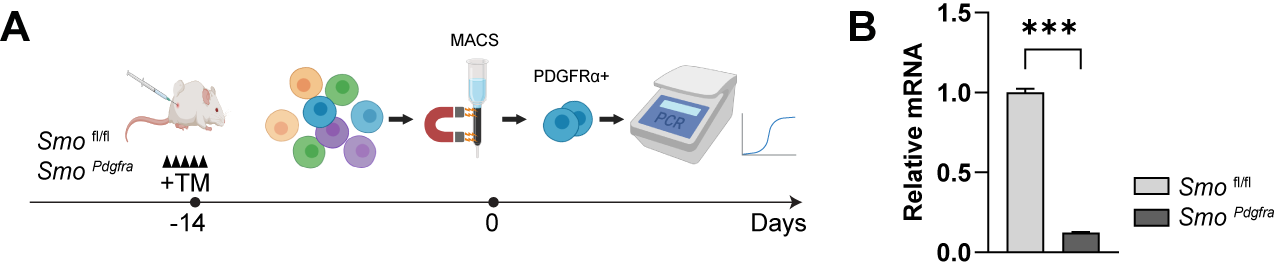


**Figure S6. Validation of *Smo* conditional deletion efficiency in calvarial *Pdgfra*+ mesenchymal cells.**

(A) Experimental schematic for the validation of genetic deletion. *Pdgfra*-Cre^ERT2^; *Smo*^fl/fl^ and *Smo*^fl/fl^ control mice received tamoxifen (TM) injections (100 mg/kg/day) for 5 days. Two weeks post-initial injection (Day 0), calvarial cells were harvested, and the PDGFRα + population was isolated via Magnetic-Activated Cell Sorting (MACS). (B) Quantitative PCR (qPCR) analysis of *Smo* mRNA expression in MACS-isolated *Pdgfra*+ mesenchymal cells, showing a significant reduction of 87.6% in the knockout group (n = 3 biological replicates per group). Data are represented as mean ± SD. ***** *P* < 0.001), assessed using a two-tailed Student’s t-test.

**Table S1.** Antibodies used.

| **Antibody** | **Source** | **Catalog #** | **Use** |
| --- | --- | --- | --- |
| Mouse Alexa Fluor 647 anti-Tubulin β3  (TUBB3) | Biolegend | 801209 | IF |
| Goat Anti-Rat AF568 | Abcam | ab175476 | IF |
| Goat anti-Rabbit AF488 | Abcam | ab150077 | IF |
| Goat anti-Rabbit AF647 | Abcam | ab150115 | IF |
| Goat anti-Mouse AF594 | Abcam | ab150116 | IF |
| Goat anti-Rabbit AF647 | Abcam | ab150079 | IF |
| Rabbit anti-osteocalcin (OCN) | Abcam | ab93876 | IF |
| Rabbit anti-Ki67 | Abcam | ab16667 | IF |
| Goat anti-Patched 1 (PTCH1) | Novus | NB100-2451 | IF |
| Rabbit anti-CD31 | Abcam | ab28364 | IF |
| Chicken anti-Tyrosine Receptor Kinase A (TrkA) | Thermo Fisher | TRKA-101Y | IF |
| Mouse anti-PDGFR alpha (PDGFα) | Abcam | ab96569 | IF |
| Rabbit anti-phospho-TrkA | Cell Signaling Technology | 4619 | IF |
| Goat anti-Gli1 | R&D | AF3455 | IF |
| Rat anti-Ly-6G | Santa Cruz | sc-53515 | IF |
| Rabbit anti- Growth Associated Protein-43 (GAP-43) GAP43 | Sigma-Aldrich | AB5220 | IF |
| IF: Immunofluorescent staining. | | | |

**Table S2.** Top 40 differentially expressed genes of each mesenchymal subcluster.

| **Stem 1** | **Stem 2** | **Fibroblastic progenitor** | **Multilineage progenitor** | **Osteoblast** |
| --- | --- | --- | --- | --- |
| Pfn1 | Col14a1 | Rbp1 | Ptprd | Col11a2 |
| Loxl2 | C1s1 | Col4a1 | Megf6 | Bglap |
| Crip1 | Mmp2 | Col4a2 | Gpx3 | Bglap2 |
| S100a6 | Col3a1 | Mgp | Clec11a | Col22a1 |
| Pkm | Clec3b | Nr2f2 | Camk4 | Cfh |
| Lgals1 | Ace | Mdk | Col8a2 | Cadm1 |
| Sh3bgrl3 | Mgst1 | Foxd1 | Col12a1 | Col13a1 |
| Anxa5 | Serping1 | Apoe | Tenm3 | Mlip |
| Tpm4 | C1ra | Cpe | Fmod | Rerg |
| Pgk1 | Fndc1 | Foxc1 | Epha3 | Col1a1 |
| Cav1 | Ifi205 | Tbx18 | Col16a1 | Cpe |
| Ran | Dcn | Lmo4 | Edil3 | Slc36a2 |
| Gapdh | C4b | Palmd | Mest | Fat3 |
| Anxa2 | Cfb | Prdm6 | Pdgfrl | Ibsp |
| Eno1 | Dpt | Alcam | Tnfrsf19 | Car3 |
| Tagln2 | Anpep | Smoc1 | Itgbl1 | Lipc |
| Ranbp1 | Slfn5 | Eln | Col11a1 | Spp1 |
| Cfl1 | Plac8 | Rgs7bp | Auts2 | Cgref1 |
| Lrrc59 | Ccl8 | Plxna2 | Adcy2 | Frmd4b |
| Col5a3 | Sfrp2 | Abcb1a | Crispld2 | Col1a2 |
| Glipr2 | Cd34 | Ctnnd2 | Prkg1 | Ifitm5 |
| Pdlim4 | Opcml | Slc38a1 | Nrp2 | Bglap3 |
| Txn1 | C3 | Slit2 | Matn4 | Pth1r |
| Actb | Dpep1 | mt-Cytb | Scarf2 | Satb2 |
| Arhgdia | Ly6a | S100a1 | Itpr1 | Slc8a3 |
| Fkbp1a | Has1 | Slc12a2 | Bcl11b | Mef2c |
| Myadm | Slc43a3 | Gnas | Sox6 | Lifr |
| Nme1 | Mt2 | Msx1 | Itga11 | Sp7 |
| Ppp1r14b | Rnase4 | Uaca | Gpc1 | Kazald1 |
| Calm1 | Col6a6 | Ildr2 | Spon1 | Col11a1 |
| Vim | Lrrn4cl | Cst3 | Cdh11 | Ano1 |
| Ybx1 | Gfpt2 | Cdh5 | Olfml3 | Dapk2 |
| Pgam1 | Abcc9 | Pmepa1 | Etv6 | Ramp1 |
| Prelid1 | Ly6c1 | Pawr | Myo1d | Sparc |
| Aldoa | Plpp3 | Foxp2 | Zfhx3 | Ptprs |
| Eif5a | Adgrd1 | Tmem176b | Rasal2 | Mical3 |
| Ostf1 | Lrmda | mt-Atp6 | Thbs2 | Cd200 |
| P4ha2 | Vegfd | Gmds | Kif26b | Insc |
| Arpc1b | Celf2 | Slc4a10 | Capn6 | Sgms2 |
| Crip2 | Gm12840 | Zic1 | Pth1r | Kcnma1 |

**Table S3.** Top 100 significantly differentially expressed genes identified by pseudobulk analysis comparing GA and control groups across the total cells.

| names | baseMean | log2FoldChange | lfcSE | stat | pvalue | padj |
| --- | --- | --- | --- | --- | --- | --- |
| Slurp1 | 1.685044 | 4.344365 | 1.723515 | 2.520642 | 0.011714081 | 0.023462177 |
| Gm32442 | 4.784854 | 4.342739 | 1.126309 | 3.855725 | 0.000115387 | 0.000310173 |
| Ggt1 | 63.00385 | 3.5743 | 0.217592 | 16.42664 | 1.23E-60 | 4.60E-59 |
| Gm15348 | 2.018609 | 3.515324 | 1.063776 | 3.304572 | 0.000951214 | 0.002269752 |
| Oosp2 | 1.274306 | 3.371696 | 1.280443 | 2.633226 | 0.008457799 | 0.017367248 |
| Gm13580 | 3.249904 | 3.301029 | 0.839021 | 3.934381 | 8.34E-05 | 0.000228136 |
| Ighv1-63 | 1.701289 | 3.275766 | 1.095512 | 2.990169 | 0.00278823 | 0.006220744 |
| Gm30191 | 1.184711 | 3.257687 | 1.273974 | 2.557106 | 0.010554699 | 0.021300477 |
| Ttc34 | 1.167292 | 3.252408 | 1.258376 | 2.584608 | 0.009748974 | 0.01979753 |
| Tigd4 | 1.143887 | 3.245745 | 1.274303 | 2.547074 | 0.010863025 | 0.021888687 |
| Gm37468 | 1.64678 | 3.208436 | 1.323059 | 2.425014 | 0.015307808 | 0.029897704 |
| Defb34 | 1.645699 | 3.193222 | 1.075397 | 2.969342 | 0.002984379 | 0.006626216 |
| Slc22a29 | 1.103137 | 3.131984 | 1.267211 | 2.471558 | 0.013452578 | 0.026603826 |
| Rnase2a | 4.320906 | 3.100527 | 0.648444 | 4.781488 | 1.74E-06 | 5.65E-06 |
| Gm32554 | 3.550695 | 3.075307 | 0.952877 | 3.227392 | 0.001249244 | 0.002933331 |
| Gm48277 | 2.062614 | 3.029618 | 1.113881 | 2.719875 | 0.006530663 | 0.013698071 |
| 3632454L22Rik | 2.04388 | 3.029226 | 1.309718 | 2.312883 | 0.020729047 | 0.039286581 |
| 2210010C04Rik | 1.003974 | 2.996087 | 1.314743 | 2.278838 | 0.022676685 | 0.042622822 |
| Gm48350 | 1.000349 | 2.995029 | 1.29633 | 2.310391 | 0.020866503 | 0.039523337 |
| Krt36 | 2.693261 | 2.99404 | 0.800741 | 3.739086 | 0.00018469 | 0.000485354 |
| 4833413E03Rik | 1.417884 | 2.98907 | 1.16121 | 2.574101 | 0.010050107 | 0.020356607 |
| Traj18 | 0.972666 | 2.982845 | 1.329293 | 2.243934 | 0.024836679 | 0.046312004 |
| Myoc | 35.85314 | 2.915445 | 0.553663 | 5.265738 | 1.40E-07 | 5.03E-07 |
| Gm7457 | 10.35384 | 2.836642 | 0.443934 | 6.389786 | 1.66E-10 | 7.42E-10 |
| Gm32913 | 5.389781 | 2.80917 | 0.896361 | 3.133971 | 0.00172458 | 0.003977942 |
| Tctex1d1 | 59.50751 | 2.791023 | 0.307087 | 9.08869 | 1.00E-19 | 7.51E-19 |
| Gm31518 | 4.553284 | 2.738722 | 1.029677 | 2.659787 | 0.007819004 | 0.016157762 |
| Lmntd2 | 1.716426 | 2.716849 | 0.992594 | 2.737119 | 0.006197978 | 0.013047379 |
| Marco | 53.54895 | 2.709456 | 1.184494 | 2.287438 | 0.022170283 | 0.041759899 |
| S100g | 1.691139 | 2.709443 | 0.947835 | 2.858561 | 0.004255674 | 0.009189125 |
| Gm47731 | 2.214101 | 2.682838 | 0.81174 | 3.305045 | 0.00094961 | 0.002266414 |
| Cxcr1 | 21.44996 | 2.656061 | 0.335272 | 7.922099 | 2.34E-15 | 1.39E-14 |
| Lrg1 | 2229.256 | 2.615873 | 0.101139 | 25.86418 | 1.69E-147 | 9.07E-145 |
| Krtdap | 59.84996 | 2.592465 | 0.362277 | 7.156034 | 8.30E-13 | 4.28E-12 |
| Retnla | 39.06559 | 2.572715 | 1.057624 | 2.432542 | 0.014993235 | 0.029337826 |
| Gm29554 | 4.160224 | 2.566414 | 0.858043 | 2.99101 | 0.002780562 | 0.006206144 |
| Cstdc6 | 2.571211 | 2.55773 | 0.73128 | 3.497606 | 0.000469455 | 0.001169108 |
| Gm14204 | 2.569398 | 2.554082 | 0.728518 | 3.505861 | 0.000455132 | 0.001135362 |
| Akain1 | 6.199975 | 2.548783 | 0.518539 | 4.915313 | 8.86E-07 | 2.96E-06 |
| Ugt3a2 | 1.551149 | 2.529603 | 1.084539 | 2.332424 | 0.019678422 | 0.037498242 |
| Gm12709 | 9.128939 | 2.521496 | 0.40595 | 6.211345 | 5.25E-10 | 2.27E-09 |
| Fam90a1b-1 | 1.075092 | 2.512749 | 1.114245 | 2.255113 | 0.024126242 | 0.045120692 |

| G6pd2 | 1.053961 | 2.507422 | 1.112634 | 2.253591 | 0.024221927 | 0.045284293 |
| --- | --- | --- | --- | --- | --- | --- |
| Foxd4 | 9.400737 | 2.480033 | 0.510036 | 4.862468 | 1.16E-06 | 3.83E-06 |
| Scgn | 1.943348 | 2.467333 | 0.901614 | 2.736575 | 0.006208243 | 0.013067741 |
| Fam3b | 1.943654 | 2.462261 | 0.844207 | 2.916655 | 0.003538068 | 0.007749553 |
| Plin5 | 6.31046 | 2.451793 | 0.47034 | 5.212809 | 1.86E-07 | 6.62E-07 |
| Asprv1 | 873.0518 | 2.447211 | 0.109963 | 22.25492 | 1.01E-109 | 1.81E-107 |
| Cstdc5 | 253.5415 | 2.421942 | 0.441242 | 5.488925 | 4.04E-08 | 1.52E-07 |
| Psg16 | 4.646929 | 2.369914 | 0.541136 | 4.379518 | 1.19E-05 | 3.57E-05 |
| Mcf2 | 2.776913 | 2.360011 | 0.790716 | 2.98465 | 0.002839027 | 0.006325125 |
| Gm38171 | 11.01355 | 2.347042 | 0.399356 | 5.877065 | 4.18E-09 | 1.70E-08 |
| Gm36231 | 2.273416 | 2.308735 | 0.960898 | 2.402685 | 0.016275205 | 0.031585889 |
| Chil5 | 145.0895 | 2.302677 | 0.141851 | 16.23309 | 2.94E-59 | 1.05E-57 |
| Prtn3 | 18622.73 | 2.297007 | 0.110347 | 20.81627 | 3.08E-96 | 3.54E-94 |
| Gm5294 | 4.441588 | 2.296491 | 0.547004 | 4.198309 | 2.69E-05 | 7.77E-05 |
| Mrgpra2b | 998.7014 | 2.290345 | 0.098167 | 23.33108 | 2.15E-120 | 5.32E-118 |
| Armc12 | 4.80685 | 2.289942 | 0.511099 | 4.480431 | 7.45E-06 | 2.28E-05 |
| Orm3 | 1.309036 | 2.289308 | 0.969218 | 2.362016 | 0.01817587 | 0.034924506 |
| Ifitm1 | 6682.902 | 2.283532 | 0.124479 | 18.34468 | 3.64E-75 | 2.14E-73 |
| Ifitm6 | 9852.066 | 2.281706 | 0.074025 | 30.82359 | 1.27E-208 | 3.10E-205 |
| Ctsg | 8685.384 | 2.263096 | 0.16447 | 13.7599 | 4.44E-43 | 8.73E-42 |
| Mrgpra2a | 179.3206 | 2.247612 | 0.140045 | 16.04925 | 5.79E-58 | 1.96E-56 |
| Gm34078 | 17.02511 | 2.232272 | 0.308903 | 7.226453 | 4.96E-13 | 2.59E-12 |
| Bpifc | 9.382103 | 2.230338 | 0.577452 | 3.862379 | 0.000112288 | 0.000302395 |
| Gm45332 | 2.96491 | 2.22302 | 0.688781 | 3.227471 | 0.001248898 | 0.00293283 |
| Gm47732 | 2.125401 | 2.218539 | 0.803781 | 2.760129 | 0.005777856 | 0.012222431 |
| Cftr | 19.71451 | 2.218398 | 0.740081 | 2.997507 | 0.002721977 | 0.006084615 |
| Gm48671 | 2.102208 | 2.216129 | 0.752617 | 2.944564 | 0.003234103 | 0.007131182 |
| Ceacam10 | 485.1234 | 2.210343 | 0.111198 | 19.87763 | 6.36E-88 | 5.61E-86 |
| Myo15b | 5.030656 | 2.198427 | 0.523618 | 4.198534 | 2.69E-05 | 7.76E-05 |
| Gm29264 | 68.98288 | 2.184282 | 0.141354 | 15.45262 | 7.24E-54 | 2.14E-52 |
| Lin28a | 103.8305 | 2.183843 | 0.128119 | 17.0454 | 3.78E-65 | 1.61E-63 |
| Wfdc21 | 25440.97 | 2.176009 | 0.076046 | 28.6142 | 4.47E-180 | 7.05E-177 |
| Gm44000 | 6.602355 | 2.175199 | 0.432731 | 5.026678 | 4.99E-07 | 1.70E-06 |
| Tacstd2 | 214.8859 | 2.172468 | 0.078897 | 27.53549 | 6.60E-167 | 7.67E-164 |
| Mpo | 9125.639 | 2.167732 | 0.1051 | 20.62545 | 1.62E-94 | 1.72E-92 |
| Mapk13 | 749.7305 | 2.143182 | 0.079348 | 27.00997 | 1.13E-160 | 9.23E-158 |
| Grxcr1 | 2.004458 | 2.138329 | 0.755722 | 2.829518 | 0.004661819 | 0.010011254 |
| Stfa2 | 111.7653 | 2.131115 | 0.54066 | 3.941691 | 8.09E-05 | 0.000221539 |
| Stfa1 | 715.0793 | 2.12216 | 0.240728 | 8.815599 | 1.19E-18 | 8.42E-18 |
| Slc22a20 | 46.52545 | 2.116121 | 0.212773 | 9.945447 | 2.64E-23 | 2.33E-22 |
| Spag6l | 17.75302 | 2.103833 | 0.31766 | 6.622913 | 3.52E-11 | 1.64E-10 |
| Gm13920 | 1.568241 | 2.09826 | 0.892863 | 2.350036 | 0.018771616 | 0.035925355 |
| Lcn2 | 52033.58 | 2.096779 | 0.066447 | 31.55587 | 1.49E-218 | 4.70E-215 |
| Gm32468 | 3.964934 | 2.09647 | 0.591554 | 3.544006 | 0.000394097 | 0.000992301 |
| 9030204H09Rik | 1.565237 | 2.094262 | 0.899921 | 2.327163 | 0.019956615 | 0.037982443 |
| Gm38575 | 92.33448 | 2.075337 | 0.18002 | 11.52838 | 9.49E-31 | 1.15E-29 |

| Il1f9 | 1111.529 | 2.06807 | 0.123233 | 16.78183 | 3.31E-63 | 1.33E-61 |
| --- | --- | --- | --- | --- | --- | --- |
| Gm20467 | 1.977529 | 2.06512 | 0.857306 | 2.408849 | 0.016002918 | 0.031098469 |
| Retnlb | 14.28557 | 2.056797 | 0.408798 | 5.031332 | 4.87E-07 | 1.66E-06 |
| Gm36860 | 1.92135 | 2.055869 | 0.761422 | 2.700037 | 0.006933176 | 0.014469465 |
| Clec2f | 3.069369 | 2.053912 | 0.64029 | 3.207783 | 0.001337622 | 0.003129532 |
| Cd177 | 3836.137 | 2.052256 | 0.087738 | 23.39063 | 5.32E-121 | 1.35E-118 |
| Prss16 | 50.5207 | 2.051044 | 0.186374 | 11.00499 | 3.62E-28 | 3.95E-27 |
| Olfr658 | 3.098498 | 2.047008 | 0.612191 | 3.34374 | 0.000826573 | 0.001989258 |
| Slfn4 | 2090.835 | 2.037651 | 0.0909 | 22.41651 | 2.72E-111 | 5.31E-109 |
| Colec10 | 7.570286 | 2.036955 | 0.745271 | 2.733173 | 0.006272745 | 0.013198478 |
| Gm14085 | 4.953711 | 2.036746 | 0.624619 | 3.260783 | 0.001111052 | 0.002624199 |
| Abhd12b | 2.651295 | 2.019464 | 0.678466 | 2.976513 | 0.002915465 | 0.006484947 |

**Table S4.** Top 100 significantly enriched Gene Ontology (GO) terms in GA compared to control groups identified by scRNA-seq analysis of the total cells.

| **ID** | **Description** | **RichFactor** | **pvalue** | **p.adjust** | **qvalue** |
| --- | --- | --- | --- | --- | --- |
| GO:0050900 | leukocyte migration | 0.102689487 | 2.12E-15 | 8.48E-12 | 7.52E-12 |
| GO:0030595 | leukocyte chemotaxis | 0.12345679 | 1.94E-13 | 3.87E-10 | 3.43E-10 |
| GO:0018149 | peptide cross-linking | 0.387096774 | 2.78E-12 | 3.71E-09 | 3.29E-09 |
| GO:0002446 | neutrophil mediated immunity | 0.302325581 | 1.32E-11 | 1.32E-08 | 1.17E-08 |
| GO:0002444 | myeloid leukocyte mediated immunity | 0.15503876 | 3.21E-11 | 2.00E-08 | 1.77E-08 |
| GO:0006935 | chemotaxis | 0.081081081 | 3.53E-11 | 2.00E-08 | 1.77E-08 |
| GO:0060326 | cell chemotaxis | 0.09495549 | 3.81E-11 | 2.00E-08 | 1.77E-08 |
| GO:0042330 | taxis | 0.080745342 | 4.00E-11 | 2.00E-08 | 1.77E-08 |
| GO:0042119 | neutrophil activation | 0.272727273 | 3.13E-10 | 1.39E-07 | 1.23E-07 |
| GO:0006909 | phagocytosis | 0.10373444 | 8.48E-10 | 3.09E-07 | 2.74E-07 |
| GO:0002274 | myeloid leukocyte activation | 0.096774194 | 8.49E-10 | 3.09E-07 | 2.74E-07 |
| GO:0007159 | leukocyte cell-cell adhesion | 0.078341014 | 1.58E-09 | 5.26E-07 | 4.67E-07 |
| GO:0036230 | granulocyte activation | 0.222222222 | 4.10E-09 | 1.26E-06 | 1.12E-06 |
| GO:0043299 | leukocyte degranulation | 0.163043478 | 4.56E-09 | 1.30E-06 | 1.16E-06 |
| GO:0042742 | defense response to bacterium | 0.07748184 | 6.25E-09 | 1.67E-06 | 1.48E-06 |
| GO:0097529 | myeloid leukocyte migration | 0.09375 | 1.37E-08 | 3.42E-06 | 3.03E-06 |
| GO:0002523 | leukocyte migration involved in inflammatory response | 0.310344828 | 1.51E-08 | 3.56E-06 | 3.16E-06 |
| GO:0001909 | leukocyte mediated cytotoxicity | 0.106951872 | 2.45E-08 | 5.45E-06 | 4.84E-06 |
| GO:0001906 | cell killing | 0.084745763 | 5.09E-08 | 1.07E-05 | 9.51E-06 |
| GO:0002366 | leukocyte activation involved in immune response | 0.079646018 | 5.44E-08 | 1.09E-05 | 9.64E-06 |
| GO:0045123 | cellular extravasation | 0.158536585 | 6.88E-08 | 1.21E-05 | 1.07E-05 |
| GO:0002263 | cell activation involved in immune response | 0.078717201 | 6.91E-08 | 1.21E-05 | 1.07E-05 |
| GO:1990266 | neutrophil migration | 0.125 | 6.97E-08 | 1.21E-05 | 1.07E-05 |
| GO:0097530 | granulocyte migration | 0.105882353 | 1.42E-07 | 2.37E-05 | 2.10E-05 |
| GO:0002697 | regulation of immune effector process | 0.065708419 | 2.83E-07 | 4.53E-05 | 4.02E-05 |
| GO:0002695 | negative regulation of leukocyte activation | 0.093137255 | 4.83E-07 | 7.44E-05 | 6.60E-05 |
| GO:0030216 | keratinocyte differentiation | 0.100591716 | 6.54E-07 | 9.69E-05 | 8.59E-05 |
| GO:0030593 | neutrophil chemotaxis | 0.13 | 7.42E-07 | 0.000106 | 9.40E-05 |
| GO:0043300 | regulation of leukocyte degranulation | 0.175438596 | 8.55E-07 | 0.000118 | 0.000105 |
| GO:0071621 | granulocyte chemotaxis | 0.108695652 | 1.11E-06 | 0.000145 | 0.000129 |
| GO:0072672 | neutrophil extravasation | 0.375 | 1.16E-06 | 0.000145 | 0.000129 |
| GO:0042554 | superoxide anion generation | 0.195652174 | 1.16E-06 | 0.000145 | 0.000129 |
| GO:0009913 | epidermal cell differentiation | 0.08045977 | 1.37E-06 | 0.000167 | 0.000148 |
| GO:0050764 | regulation of phagocytosis | 0.113821138 | 1.44E-06 | 0.00017 | 0.00015 |
| GO:0050727 | regulation of inflammatory response | 0.066997519 | 1.66E-06 | 0.000189 | 0.000168 |
| GO:0050866 | negative regulation of cell activation | 0.084821429 | 1.99E-06 | 0.000221 | 0.000196 |
| GO:0043320 | natural killer cell degranulation | 0.454545455 | 3.08E-06 | 0.000333 | 0.000295 |
| GO:0002228 | natural killer cell mediated immunity | 0.12244898 | 3.74E-06 | 0.000394 | 0.000349 |
| GO:0051873 | killing by host of symbiont cells | 0.24137931 | 4.13E-06 | 0.000424 | 0.000376 |
| GO:0072593 | reactive oxygen species metabolic process | 0.076045627 | 5.73E-06 | 0.000573 | 0.000508 |
| GO:0051250 | negative regulation of lymphocyte activation | 0.089385475 | 6.49E-06 | 0.000633 | 0.000562 |
| GO:0045730 | respiratory burst | 0.225806452 | 6.68E-06 | 0.000636 | 0.000564 |

| GO:0002687 | positive regulation of leukocyte migration | 0.088888889 | 6.97E-06 | 0.000648 | 0.000575 |
| --- | --- | --- | --- | --- | --- |
| GO:0070944 | neutrophil-mediated killing of bacterium | 0.384615385 | 8.24E-06 | 0.000749 | 0.000664 |
| GO:0006801 | superoxide metabolic process | 0.136986301 | 8.74E-06 | 0.000767 | 0.00068 |
| GO:2000377 | regulation of reactive oxygen species metabolic process | 0.09202454 | 8.82E-06 | 0.000767 | 0.00068 |
| GO:0002703 | regulation of leukocyte mediated immunity | 0.068965517 | 9.52E-06 | 0.00081 | 0.000719 |
| GO:1903037 | regulation of leukocyte cell-cell adhesion | 0.063613232 | 9.80E-06 | 0.000817 | 0.000725 |
| GO:0002532 | production of molecular mediator involved in inflammatory  response | 0.111111111 | 1.04E-05 | 0.000845 | 0.000749 |
| GO:0002886 | regulation of myeloid leukocyte mediated immunity | 0.133333333 | 1.12E-05 | 0.000894 | 0.000793 |
| GO:0042267 | natural killer cell mediated cytotoxicity | 0.11827957 | 1.32E-05 | 0.001038 | 0.00092 |
| GO:0043588 | skin development | 0.065527066 | 1.38E-05 | 0.001058 | 0.000938 |
| GO:0002685 | regulation of leukocyte migration | 0.073929961 | 1.45E-05 | 0.001096 | 0.000972 |
| GO:0051607 | defense response to virus | 0.066869301 | 1.54E-05 | 0.001141 | 0.001012 |
| GO:0002285 | lymphocyte activation involved in immune response | 0.075 | 2.00E-05 | 0.001454 | 0.001289 |
| GO:0010466 | negative regulation of peptidase activity | 0.074380165 | 2.23E-05 | 0.001596 | 0.001415 |
| GO:0006739 | NADP metabolic process | 0.156862745 | 2.54E-05 | 0.001757 | 0.001558 |
| GO:0042129 | regulation of T cell proliferation | 0.08 | 2.61E-05 | 0.001757 | 0.001558 |
| GO:0002430 | complement receptor mediated signaling pathway | 0.3125 | 2.63E-05 | 0.001757 | 0.001558 |
| GO:0070943 | neutrophil-mediated killing of symbiont cell | 0.3125 | 2.63E-05 | 0.001757 | 0.001558 |
| GO:0042098 | T cell proliferation | 0.073170732 | 2.78E-05 | 0.001822 | 0.001616 |
| GO:0035710 | CD4-positive, alpha-beta T cell activation | 0.09352518 | 2.92E-05 | 0.001881 | 0.001668 |
| GO:0050863 | regulation of T cell activation | 0.060453401 | 3.35E-05 | 0.002126 | 0.001885 |
| GO:0071674 | mononuclear cell migration | 0.074561404 | 3.63E-05 | 0.002252 | 0.001997 |
| GO:0043312 | neutrophil degranulation | 0.294117647 | 3.66E-05 | 0.002252 | 0.001997 |
| GO:0052547 | regulation of peptidase activity | 0.06 | 3.77E-05 | 0.002287 | 0.002028 |
| GO:0051346 | negative regulation of hydrolase activity | 0.066006601 | 4.45E-05 | 0.002656 | 0.002355 |
| GO:0070942 | neutrophil mediated cytotoxicity | 0.277777778 | 4.97E-05 | 0.002923 | 0.002592 |
| GO:0061756 | leukocyte adhesion to vascular endothelial cell | 0.142857143 | 5.10E-05 | 0.002957 | 0.002622 |
| GO:0006691 | leukotriene metabolic process | 0.206896552 | 5.27E-05 | 0.003013 | 0.002672 |
| GO:0002323 | natural killer cell activation involved in immune response | 0.166666667 | 5.46E-05 | 0.003072 | 0.002725 |
| GO:0045637 | regulation of myeloid cell differentiation | 0.072033898 | 5.60E-05 | 0.003072 | 0.002725 |
| GO:0045576 | mast cell activation | 0.111111111 | 5.61E-05 | 0.003072 | 0.002725 |
| GO:0052803 | imidazole-containing compound metabolic process | 0.4 | 5.94E-05 | 0.003212 | 0.002848 |
| GO:0050766 | positive regulation of phagocytosis | 0.10989011 | 6.17E-05 | 0.003289 | 0.002917 |
| GO:2000696 | regulation of epithelial cell differentiation involved in  kidney development | 0.263157895 | 6.61E-05 | 0.00348 | 0.003086 |
| GO:0045088 | regulation of innate immune response | 0.054435484 | 6.78E-05 | 0.00352 | 0.003122 |
| GO:0051156 | glucose 6-phosphate metabolic process | 0.193548387 | 7.85E-05 | 0.004011 | 0.003557 |
| GO:0006639 | acylglycerol metabolic process | 0.08496732 | 7.92E-05 | 0.004011 | 0.003557 |
| GO:0002275 | myeloid cell activation involved in immune response | 0.096491228 | 8.91E-05 | 0.004412 | 0.003913 |
| GO:0006638 | neutral lipid metabolic process | 0.083870968 | 9.04E-05 | 0.004412 | 0.003913 |
| GO:0050729 | positive regulation of inflammatory response | 0.083870968 | 9.04E-05 | 0.004412 | 0.003913 |
| GO:0031640 | killing of cells of another organism | 0.095652174 | 9.64E-05 | 0.004593 | 0.004073 |
| GO:0141061 | disruption of cell in another organism | 0.095652174 | 9.64E-05 | 0.004593 | 0.004073 |
| GO:0006098 | pentose-phosphate shunt | 0.238095238 | 0.000111 | 0.005234 | 0.004642 |
| GO:0006641 | triglyceride metabolic process | 0.094017094 | 0.000113 | 0.005242 | 0.004648 |
| GO:1903706 | regulation of hemopoiesis | 0.054585153 | 0.000119 | 0.005485 | 0.004864 |

| GO:0045861 | negative regulation of proteolysis | 0.059490085 | 0.000127 | 0.005791 | 0.005135 |
| --- | --- | --- | --- | --- | --- |
| GO:0046631 | alpha-beta T cell activation | 0.072815534 | 0.000134 | 0.005993 | 0.005315 |
| GO:0043313 | regulation of neutrophil degranulation | 0.333333333 | 0.000135 | 0.005993 | 0.005315 |
| GO:0008544 | epidermis development | 0.056234719 | 0.000143 | 0.006288 | 0.005576 |
| GO:0045071 | negative regulation of viral genome replication | 0.123076923 | 0.00015 | 0.006532 | 0.005793 |
| GO:0002237 | response to molecule of bacterial origin | 0.052631579 | 0.000157 | 0.0067 | 0.005942 |
| GO:2000379 | positive regulation of reactive oxygen species metabolic  process | 0.108433735 | 0.000158 | 0.0067 | 0.005942 |
| GO:0009615 | response to virus | 0.055825243 | 0.000159 | 0.0067 | 0.005942 |
| GO:0002283 | neutrophil activation involved in immune response | 0.217391304 | 0.000177 | 0.007291 | 0.006466 |
| GO:0009164 | nucleoside catabolic process | 0.217391304 | 0.000177 | 0.007291 | 0.006466 |
| GO:0046651 | lymphocyte proliferation | 0.05952381 | 0.000181 | 0.007407 | 0.006569 |
| GO:0019370 | leukotriene biosynthetic process | 0.307692308 | 0.000191 | 0.007644 | 0.006779 |
| GO:0072182 | regulation of nephron tubule epithelial cell differentiation | 0.307692308 | 0.000191 | 0.007644 | 0.006779 |

**Table S5.** Top 100 significantly differentially expressed genes identified by pseudobulk analysis comparing GA and control groups across the mesenchymal lineage subpopulation.

| names | baseMean | log2FoldChange | lfcSE | stat | pvalue | padj |
| --- | --- | --- | --- | --- | --- | --- |
| Gm32442 | 5.837758 | 5.027616 | 1.013795 | 4.959206 | 7.08E-07 | 1.01E-05 |
| Cstdc5 | 2.688869 | 4.879778 | 1.657986 | 2.943196 | 0.00324843 | 0.016080153 |
| Krt6a | 10.13955 | 4.419321 | 1.699807 | 2.599895 | 0.009325224 | 0.038335277 |
| Bpifc | 4.436978 | 3.856189 | 1.39492 | 2.764451 | 0.005701866 | 0.025610869 |
| Krt36 | 3.330891 | 3.742828 | 0.843592 | 4.436773 | 9.13E-06 | 0.000100066 |
| Myoc | 40.79456 | 3.667813 | 0.512529 | 7.156307 | 8.29E-13 | 3.33E-11 |
| Gm32913 | 5.550718 | 3.447981 | 1.286267 | 2.680611 | 0.007348795 | 0.031555389 |
| Krtdap | 72.2006 | 3.422831 | 0.284927 | 12.01302 | 3.04E-33 | 8.59E-31 |
| Gm12524 | 2.532748 | 3.321944 | 1.264136 | 2.627838 | 0.008592935 | 0.035845781 |
| Xlr4a | 9.222117 | 3.259322 | 0.487464 | 6.686286 | 2.29E-11 | 7.52E-10 |
| Lrg1 | 30.28311 | 3.14056 | 0.379254 | 8.280886 | 1.22E-16 | 7.66E-15 |
| Ifit1bl1 | 5.393838 | 2.905506 | 0.744476 | 3.902752 | 9.51E-05 | 0.000792885 |
| Lcn2 | 533.5976 | 2.872822 | 0.306619 | 9.369365 | 7.30E-21 | 6.77E-19 |
| Ceacam10 | 8.150496 | 2.85527 | 0.550896 | 5.182951 | 2.18E-07 | 3.49E-06 |
| Colec10 | 8.935251 | 2.766425 | 0.889957 | 3.108493 | 0.001880443 | 0.010243467 |
| Gm12153 | 4.090562 | 2.764217 | 0.977294 | 2.828439 | 0.004677555 | 0.021854341 |
| Wfdc21 | 210.1805 | 2.726003 | 0.245066 | 11.12355 | 9.64E-29 | 2.07E-26 |
| Itgb2l | 17.19242 | 2.718477 | 0.324414 | 8.379646 | 5.31E-17 | 3.47E-15 |
| Mgam | 11.31541 | 2.712534 | 0.38889 | 6.975072 | 3.06E-12 | 1.14E-10 |
| Stfa2l1 | 29.94383 | 2.680793 | 0.733817 | 3.653219 | 0.000258974 | 0.00187615 |
| Ifitm6 | 147.0978 | 2.654597 | 0.276673 | 9.594701 | 8.42E-22 | 8.81E-20 |
| Adarb2 | 83.44792 | 2.6531 | 0.791626 | 3.351458 | 0.000803873 | 0.004938449 |
| Cd177 | 37.53919 | 2.604759 | 0.343316 | 7.587063 | 3.27E-14 | 1.53E-12 |
| Asprv1 | 15.17186 | 2.518559 | 0.481092 | 5.235087 | 1.65E-07 | 2.72E-06 |
| Mrgpra2a | 3.044928 | 2.491631 | 0.63504 | 3.923581 | 8.72E-05 | 0.000736378 |
| Ltf | 327.9655 | 2.473166 | 0.400243 | 6.179159 | 6.44E-10 | 1.69E-08 |
| Mrgpra2b | 7.946417 | 2.472618 | 0.426366 | 5.79929 | 6.66E-09 | 1.44E-07 |
| S100a8 | 6053.673 | 2.46159 | 0.248144 | 9.920012 | 3.41E-23 | 3.91E-21 |
| Gm32647 | 26.12332 | 2.453691 | 0.631199 | 3.887349 | 0.000101345 | 0.000839135 |
| Cyp3a13 | 3.367114 | 2.450696 | 0.594688 | 4.120978 | 3.77E-05 | 0.000352628 |
| Slco4c1 | 4.069525 | 2.412734 | 0.666281 | 3.621195 | 0.000293246 | 0.002084403 |
| Dmkn | 1681.485 | 2.381379 | 0.201523 | 11.81693 | 3.19E-32 | 8.46E-30 |
| 1700010H22Rik | 4.3133 | 2.379109 | 0.823438 | 2.889238 | 0.003861768 | 0.018606204 |
| Cstdc4 | 29.72572 | 2.367298 | 0.679381 | 3.484494 | 0.000493069 | 0.003231409 |
| Fer1l6 | 2.834424 | 2.357752 | 0.638365 | 3.693423 | 0.000221255 | 0.001645024 |
| S100a9 | 6944.209 | 2.350631 | 0.229997 | 10.22026 | 1.61E-24 | 2.15E-22 |
| Ly6g | 27.15963 | 2.342763 | 0.403032 | 5.812849 | 6.14E-09 | 1.34E-07 |
| Rsad2 | 299.0714 | 2.331802 | 0.456792 | 5.10473 | 3.31E-07 | 5.11E-06 |
| Pglyrp1 | 102.1861 | 2.326867 | 0.212593 | 10.94517 | 7.01E-28 | 1.33E-25 |
| 2610316D01Rik | 5.240532 | 2.326056 | 0.748161 | 3.109031 | 0.00187702 | 0.010231388 |
| Tmem182 | 5.209057 | 2.325354 | 0.755767 | 3.076815 | 0.00209225 | 0.011174906 |
| Ankrd22 | 5.172345 | 2.300486 | 0.462569 | 4.973276 | 6.58E-07 | 9.49E-06 |

| Ngp | 898.8624 | 2.284835 | 0.372991 | 6.125708 | 9.03E-10 | 2.31E-08 |
| --- | --- | --- | --- | --- | --- | --- |
| Vgf | 3.73803 | 2.279245 | 0.786573 | 2.89769 | 0.003759215 | 0.018173999 |
| Kcnc2 | 59.91289 | 2.272466 | 0.37511 | 6.058136 | 1.38E-09 | 3.39E-08 |
| C130071C03Rik | 3.058703 | 2.270255 | 0.899975 | 2.522576 | 0.011649872 | 0.045573847 |
| Prg2 | 4.014685 | 2.258621 | 0.532893 | 4.23841 | 2.25E-05 | 0.000223319 |
| Cntn5 | 5.317446 | 2.251181 | 0.610602 | 3.686819 | 0.000227075 | 0.001680193 |
| Galnt15 | 16.7608 | 2.21756 | 0.407541 | 5.441318 | 5.29E-08 | 9.61E-07 |
| Cebpe | 10.35618 | 2.192662 | 0.611359 | 3.586536 | 0.0003351 | 0.002330188 |
| Chil1 | 21.57808 | 2.184036 | 0.375086 | 5.822768 | 5.79E-09 | 1.27E-07 |
| Nmu | 3.509678 | 2.177463 | 0.777978 | 2.798875 | 0.005128103 | 0.023531878 |
| Plac9a | 3.500759 | 2.159175 | 0.598471 | 3.607819 | 0.000308782 | 0.002181131 |
| Arhgef38 | 12.99161 | 2.145129 | 0.744233 | 2.882334 | 0.003947411 | 0.018922155 |
| Camp | 699.1627 | 2.131301 | 0.386394 | 5.515868 | 3.47E-08 | 6.56E-07 |
| Cd55b | 54.9435 | 2.115063 | 0.245715 | 8.607775 | 7.45E-18 | 5.16E-16 |
| Elmod1 | 7.33726 | 2.111233 | 0.72188 | 2.92463 | 0.003448656 | 0.01688419 |
| Abca13 | 13.40009 | 2.085669 | 0.470396 | 4.433862 | 9.26E-06 | 0.000101231 |
| Ifit1 | 1026.077 | 2.084228 | 0.289256 | 7.205478 | 5.78E-13 | 2.37E-11 |
| 9830107B12Rik | 11.63744 | 2.083897 | 0.465716 | 4.474611 | 7.66E-06 | 8.57E-05 |
| Scrg1 | 9.149209 | 2.081418 | 0.426834 | 4.876413 | 1.08E-06 | 1.46E-05 |
| Slfn4 | 60.26876 | 2.05201 | 0.207155 | 9.905677 | 3.93E-23 | 4.45E-21 |
| Padi4 | 22.5534 | 2.046454 | 0.335958 | 6.091393 | 1.12E-09 | 2.81E-08 |
| Tgfa | 8.309559 | 2.039458 | 0.681453 | 2.992808 | 0.002764234 | 0.014043734 |
| Gm7361 | 3.819046 | 2.034716 | 0.543973 | 3.740473 | 0.000183674 | 0.001404362 |
| Fpr1 | 12.18305 | 2.030541 | 0.445294 | 4.56 | 5.12E-06 | 5.98E-05 |
| Oas3 | 30.36613 | 2.027166 | 0.368935 | 5.494649 | 3.91E-08 | 7.33E-07 |
| Retnlg | 830.7683 | 2.025627 | 0.349739 | 5.791821 | 6.96E-09 | 1.50E-07 |
| Upp1 | 15.00502 | 2.013608 | 0.648231 | 3.106314 | 0.001894356 | 0.010309327 |
| Dmp1 | 420.4762 | 2.012317 | 0.581999 | 3.457599 | 0.000545013 | 0.003532282 |
| Nfe2 | 16.93776 | 2.01208 | 0.272672 | 7.379107 | 1.59E-13 | 6.97E-12 |
| BC055402 | 5.279318 | 2.009069 | 0.661753 | 3.035981 | 0.002397543 | 0.012506421 |
| Ldhc | 11.22087 | 1.989964 | 0.608764 | 3.268857 | 0.001079827 | 0.006357608 |
| Mmp8 | 90.13962 | 1.98546 | 0.346644 | 5.727663 | 1.02E-08 | 2.13E-07 |
| Slc2a5 | 8.012355 | 1.980092 | 0.423562 | 4.674854 | 2.94E-06 | 3.62E-05 |
| Ackr1 | 51.16217 | 1.976305 | 0.245779 | 8.040991 | 8.91E-16 | 4.88E-14 |
| Mcemp1 | 47.82967 | 1.97119 | 0.244799 | 8.052269 | 8.13E-16 | 4.48E-14 |
| Ccl12 | 19.09139 | 1.970867 | 0.749692 | 2.628901 | 0.008566138 | 0.03576036 |
| Fpr2 | 19.86055 | 1.95043 | 0.301085 | 6.47801 | 9.29E-11 | 2.84E-09 |
| Oas2 | 82.76376 | 1.940629 | 0.116025 | 16.72591 | 8.49E-63 | 7.58E-60 |
| Cadm3 | 111.1538 | 1.931242 | 0.206735 | 9.34165 | 9.48E-21 | 8.70E-19 |
| Krt83 | 12.75761 | 1.924848 | 0.340774 | 5.648459 | 1.62E-08 | 3.31E-07 |
| Sgca | 12.40334 | 1.903657 | 0.317596 | 5.993965 | 2.05E-09 | 4.88E-08 |
| Cd300lf | 31.43358 | 1.902412 | 0.287061 | 6.627212 | 3.42E-11 | 1.09E-09 |
| Efhd1 | 258.9736 | 1.889571 | 0.20084 | 9.408347 | 5.04E-21 | 4.81E-19 |
| Ifi213 | 2.74442 | 1.888286 | 0.615995 | 3.065426 | 0.002173603 | 0.011540425 |
| 1700047M11Rik | 11.49841 | 1.884992 | 0.438854 | 4.295261 | 1.74E-05 | 0.000176822 |
| F730016J06Rik | 6.239194 | 1.884525 | 0.512111 | 3.679913 | 0.000233314 | 0.001716632 |

| Ifi44 | 132.321 | 1.850005 | 0.236343 | 7.827642 | 4.97E-15 | 2.56E-13 |
| --- | --- | --- | --- | --- | --- | --- |
| Irf7 | 668.9465 | 1.846102 | 0.080658 | 22.88806 | 6.11E-116 | 2.59E-112 |
| Ifit3b | 121.2151 | 1.84402 | 0.438815 | 4.202276 | 2.64E-05 | 0.000256302 |
| Gpr1 | 5.538662 | 1.8432 | 0.51946 | 3.548304 | 0.000387721 | 0.002636688 |
| Klra17 | 2.903683 | 1.834165 | 0.699293 | 2.622883 | 0.008718914 | 0.036282137 |
| Grid2 | 104.8122 | 1.827252 | 0.290736 | 6.284906 | 3.28E-10 | 9.08E-09 |
| 4930438A08Rik | 2.640291 | 1.811584 | 0.727317 | 2.490775 | 0.012746487 | 0.048824858 |
| Slc13a5 | 81.3186 | 1.804398 | 0.263486 | 6.848176 | 7.48E-12 | 2.63E-10 |
| Oasl1 | 88.30965 | 1.800485 | 0.272227 | 6.613911 | 3.74E-11 | 1.19E-09 |
| Cd33 | 27.47817 | 1.793581 | 0.234501 | 7.648508 | 2.03E-14 | 9.80E-13 |
| Cmpk2 | 56.46459 | 1.790985 | 0.38132 | 4.696804 | 2.64E-06 | 3.30E-05 |
| Tarm1 | 5.658464 | 1.785535 | 0.409387 | 4.36148 | 1.29E-05 | 0.00013603 |

**Table S6.** Top 100 significantly enriched Gene Ontology (GO) terms in GA compared to control groups identified by scRNA-seq analysis of the mesenchymal lineage subpopulation.

| **ID** | **Description** | **RichFactor** | **pvalue** | **p.adjust** | **qvalue** |
| --- | --- | --- | --- | --- | --- |
| GO:0051607 | defense response to virus | 0.097264 | 4.66E-20 | 1.58E-16 | 1.23E-16 |
| GO:0009615 | response to virus | 0.082524 | 5.43E-19 | 9.22E-16 | 7.17E-16 |
| GO:0045088 | regulation of innate immune response | 0.068548 | 1.59E-16 | 1.44E-13 | 1.12E-13 |
| GO:0030595 | leukocyte chemotaxis | 0.102881 | 1.70E-16 | 1.44E-13 | 1.12E-13 |
| GO:0050900 | leukocyte migration | 0.070905 | 1.24E-14 | 8.42E-12 | 6.54E-12 |
| GO:0048525 | negative regulation of viral process | 0.147826 | 3.07E-14 | 1.73E-11 | 1.35E-11 |
| GO:0060326 | cell chemotaxis | 0.077151 | 4.28E-14 | 2.08E-11 | 1.61E-11 |
| GO:0030593 | neutrophil chemotaxis | 0.16 | 4.91E-14 | 2.08E-11 | 1.62E-11 |
| GO:0045089 | positive regulation of innate immune response | 0.069307 | 6.36E-14 | 2.40E-11 | 1.86E-11 |
| GO:0042742 | defense response to bacterium | 0.067797 | 1.09E-13 | 3.72E-11 | 2.89E-11 |
| GO:1990266 | neutrophil migration | 0.132813 | 1.88E-13 | 5.81E-11 | 4.52E-11 |
| GO:0002833 | positive regulation of response to biotic stimulus | 0.064516 | 3.69E-13 | 1.04E-10 | 8.10E-11 |
| GO:0097529 | myeloid leukocyte migration | 0.085938 | 4.68E-13 | 1.22E-10 | 9.50E-11 |
| GO:0071621 | granulocyte chemotaxis | 0.123188 | 6.62E-13 | 1.61E-10 | 1.25E-10 |
| GO:0006935 | chemotaxis | 0.060291 | 7.49E-13 | 1.69E-10 | 1.32E-10 |
| GO:0042330 | taxis | 0.060041 | 8.30E-13 | 1.76E-10 | 1.37E-10 |
| GO:0140374 | antiviral innate immune response | 0.19403 | 8.98E-13 | 1.79E-10 | 1.39E-10 |
| GO:0032102 | negative regulation of response to external  stimulus | 0.06338 | 1.50E-12 | 2.82E-10 | 2.19E-10 |
| GO:0097530 | granulocyte migration | 0.105882 | 1.91E-12 | 3.41E-10 | 2.65E-10 |
| GO:0035456 | response to interferon-beta | 0.168831 | 5.85E-12 | 9.94E-10 | 7.72E-10 |
| GO:0002218 | activation of innate immune response | 0.070988 | 7.24E-12 | 1.17E-09 | 9.10E-10 |
| GO:0035458 | cellular response to interferon-beta | 0.176471 | 2.24E-11 | 3.45E-09 | 2.68E-09 |
| GO:0002832 | negative regulation of response to biotic stimulus | 0.107383 | 2.66E-11 | 3.93E-09 | 3.05E-09 |
| GO:0031348 | negative regulation of defense response | 0.071918 | 4.74E-11 | 6.71E-09 | 5.21E-09 |
| GO:0140888 | interferon-mediated signaling pathway | 0.126126 | 5.32E-11 | 7.23E-09 | 5.62E-09 |
| GO:0007159 | leukocyte cell-cell adhesion | 0.057604 | 7.98E-11 | 1.04E-08 | 8.09E-09 |
| GO:0050792 | regulation of viral process | 0.087629 | 1.63E-10 | 2.05E-08 | 1.59E-08 |
| GO:0050777 | negative regulation of immune response | 0.080717 | 1.81E-10 | 2.20E-08 | 1.71E-08 |
| GO:0002221 | pattern recognition receptor signaling pathway | 0.070671 | 1.89E-10 | 2.21E-08 | 1.72E-08 |
| GO:0045071 | negative regulation of viral genome replication | 0.169231 | 2.48E-10 | 2.81E-08 | 2.18E-08 |
| GO:0002758 | innate immune response-activating signaling  pathway | 0.068027 | 3.70E-10 | 4.05E-08 | 3.14E-08 |
| GO:1903900 | regulation of viral life cycle | 0.092025 | 9.95E-10 | 1.06E-07 | 8.20E-08 |
| GO:0002237 | response to molecule of bacterial origin | 0.050607 | 1.18E-09 | 1.21E-07 | 9.40E-08 |
| GO:0034340 | response to type I interferon | 0.121212 | 2.05E-09 | 2.05E-07 | 1.59E-07 |
| GO:0032496 | response to lipopolysaccharide | 0.05042 | 2.74E-09 | 2.66E-07 | 2.07E-07 |
| GO:0042554 | superoxide anion generation | 0.195652 | 2.82E-09 | 2.66E-07 | 2.07E-07 |
| GO:0002697 | regulation of immune effector process | 0.049281 | 4.28E-09 | 3.93E-07 | 3.05E-07 |
| GO:0071222 | cellular response to lipopolysaccharide | 0.058824 | 4.56E-09 | 4.07E-07 | 3.16E-07 |
| GO:0002274 | myeloid leukocyte activation | 0.064516 | 6.53E-09 | 5.55E-07 | 4.31E-07 |
| GO:0019058 | viral life cycle | 0.064516 | 6.53E-09 | 5.55E-07 | 4.31E-07 |
| GO:0071219 | cellular response to molecule of bacterial origin | 0.057307 | 7.09E-09 | 5.87E-07 | 4.56E-07 |

| GO:0001959 | regulation of cytokine-mediated signaling pathway | 0.086957 | 7.55E-09 | 6.10E-07 | 4.74E-07 |
| --- | --- | --- | --- | --- | --- |
| GO:0060337 | type I interferon-mediated signaling pathway | 0.119565 | 1.12E-08 | 8.41E-07 | 6.54E-07 |
| GO:0071357 | cellular response to type I interferon | 0.119565 | 1.12E-08 | 8.41E-07 | 6.54E-07 |
| GO:0070106 | interleukin-27-mediated signaling pathway | 0.4 | 1.13E-08 | 8.41E-07 | 6.54E-07 |
| GO:0016032 | viral process | 0.05571 | 1.14E-08 | 8.41E-07 | 6.54E-07 |
| GO:0002685 | regulation of leukocyte migration | 0.066148 | 1.19E-08 | 8.63E-07 | 6.70E-07 |
| GO:0002688 | regulation of leukocyte chemotaxis | 0.090909 | 1.52E-08 | 1.08E-06 | 8.38E-07 |
| GO:0060759 | regulation of response to cytokine stimulus | 0.081871 | 1.64E-08 | 1.13E-06 | 8.80E-07 |
| GO:0071216 | cellular response to biotic stimulus | 0.05291 | 2.69E-08 | 1.81E-06 | 1.41E-06 |
| GO:0045069 | regulation of viral genome replication | 0.11 | 2.72E-08 | 1.81E-06 | 1.41E-06 |
| GO:0062207 | regulation of pattern recognition receptor signaling  pathway | 0.076503 | 3.86E-08 | 2.52E-06 | 1.96E-06 |
| GO:0031664 | regulation of lipopolysaccharide-mediated signaling  pathway | 0.233333 | 4.61E-08 | 2.95E-06 | 2.29E-06 |
| GO:0090022 | regulation of neutrophil chemotaxis | 0.173913 | 5.80E-08 | 3.65E-06 | 2.83E-06 |
| GO:0019079 | viral genome replication | 0.089552 | 6.48E-08 | 4.00E-06 | 3.11E-06 |
| GO:0006959 | humoral immune response | 0.055046 | 7.45E-08 | 4.52E-06 | 3.51E-06 |
| GO:0050727 | regulation of inflammatory response | 0.049628 | 7.67E-08 | 4.57E-06 | 3.55E-06 |
| GO:0072593 | reactive oxygen species metabolic process | 0.060837 | 1.03E-07 | 6.01E-06 | 4.67E-06 |
| GO:0071622 | regulation of granulocyte chemotaxis | 0.130435 | 1.15E-07 | 6.59E-06 | 5.12E-06 |
| GO:0045824 | negative regulation of innate immune response | 0.094017 | 1.40E-07 | 7.91E-06 | 6.15E-06 |
| GO:0006801 | superoxide metabolic process | 0.123288 | 1.88E-07 | 1.05E-05 | 8.14E-06 |
| GO:0002444 | myeloid leukocyte mediated immunity | 0.085271 | 3.79E-07 | 2.07E-05 | 1.61E-05 |
| GO:0001906 | cell killing | 0.054237 | 4.84E-07 | 2.61E-05 | 2.03E-05 |
| GO:0002753 | cytoplasmic pattern recognition receptor signaling  pathway | 0.067358 | 5.17E-07 | 2.74E-05 | 2.13E-05 |
| GO:2000377 | regulation of reactive oxygen species metabolic  process | 0.07362 | 5.51E-07 | 2.88E-05 | 2.24E-05 |
| GO:0001961 | positive regulation of cytokine-mediated signaling  pathway | 0.131148 | 5.62E-07 | 2.89E-05 | 2.25E-05 |
| GO:0035739 | CD4-positive, alpha-beta T cell proliferation | 0.222222 | 5.96E-07 | 3.02E-05 | 2.35E-05 |
| GO:0002446 | neutrophil mediated immunity | 0.162791 | 6.40E-07 | 3.19E-05 | 2.48E-05 |
| GO:0070167 | regulation of biomineral tissue development | 0.091743 | 6.54E-07 | 3.22E-05 | 2.50E-05 |
| GO:1902622 | regulation of neutrophil migration | 0.126984 | 7.24E-07 | 3.51E-05 | 2.73E-05 |
| GO:0007204 | positive regulation of cytosolic calcium ion  concentration | 0.065 | 7.74E-07 | 3.70E-05 | 2.88E-05 |
| GO:0032479 | regulation of type I interferon production | 0.078014 | 9.24E-07 | 4.36E-05 | 3.38E-05 |
| GO:0030282 | bone mineralization | 0.076923 | 1.06E-06 | 4.86E-05 | 3.78E-05 |
| GO:0031640 | killing of cells of another organism | 0.086957 | 1.07E-06 | 4.86E-05 | 3.78E-05 |
| GO:0141061 | disruption of cell in another organism | 0.086957 | 1.07E-06 | 4.86E-05 | 3.78E-05 |
| GO:0006909 | phagocytosis | 0.058091 | 1.12E-06 | 5.02E-05 | 3.90E-05 |
| GO:0030500 | regulation of bone mineralization | 0.1 | 1.15E-06 | 5.07E-05 | 3.94E-05 |
| GO:0060760 | positive regulation of response to cytokine stimulus | 0.119403 | 1.17E-06 | 5.10E-05 | 3.96E-05 |
| GO:0032606 | type I interferon production | 0.075342 | 1.30E-06 | 5.61E-05 | 4.36E-05 |
| GO:0045730 | respiratory burst | 0.193548 | 1.42E-06 | 6.05E-05 | 4.70E-05 |
| GO:0030278 | regulation of ossification | 0.074324 | 1.49E-06 | 6.25E-05 | 4.86E-05 |
| GO:0002687 | positive regulation of leukocyte migration | 0.066667 | 1.58E-06 | 6.55E-05 | 5.09E-05 |
| GO:0060338 | regulation of type I interferon-mediated signaling  pathway | 0.142857 | 1.61E-06 | 6.57E-05 | 5.10E-05 |
| GO:0002699 | positive regulation of immune effector process | 0.049383 | 1.66E-06 | 6.70E-05 | 5.20E-05 |

| GO:0070663 | regulation of leukocyte proliferation | 0.052448 | 1.68E-06 | 6.70E-05 | 5.20E-05 |
| --- | --- | --- | --- | --- | --- |
| GO:0035455 | response to interferon-alpha | 0.181818 | 2.10E-06 | 8.30E-05 | 6.45E-05 |
| GO:0070486 | leukocyte aggregation | 0.263158 | 2.18E-06 | 8.51E-05 | 6.61E-05 |
| GO:0032728 | positive regulation of interferon-beta production | 0.134615 | 2.43E-06 | 9.36E-05 | 7.28E-05 |
| GO:0050670 | regulation of lymphocyte proliferation | 0.054054 | 2.62E-06 | 0.0001 | 7.77E-05 |
| GO:0031589 | cell-substrate adhesion | 0.045093 | 2.70E-06 | 0.000102 | 7.92E-05 |
| GO:0032602 | chemokine production | 0.078125 | 2.86E-06 | 0.000107 | 8.29E-05 |
| GO:0031214 | biomineral tissue development | 0.062827 | 2.94E-06 | 0.000108 | 8.42E-05 |
| GO:0032944 | regulation of mononuclear cell proliferation | 0.05303 | 3.27E-06 | 0.00012 | 9.29E-05 |
| GO:0071674 | mononuclear cell migration | 0.057018 | 3.34E-06 | 0.00012 | 9.36E-05 |
| GO:0035457 | cellular response to interferon-alpha | 0.238095 | 3.74E-06 | 0.000134 | 0.000104 |
| GO:0050920 | regulation of chemotaxis | 0.055319 | 4.64E-06 | 0.000164 | 0.000128 |
| GO:0042129 | regulation of T cell proliferation | 0.06 | 4.72E-06 | 0.000165 | 0.000128 |
| GO:0090023 | positive regulation of neutrophil chemotaxis | 0.157895 | 4.99E-06 | 0.000173 | 0.000134 |
| GO:1903555 | regulation of tumor necrosis factor superfamily  cytokine production | 0.059406 | 5.23E-06 | 0.000178 | 0.000138 |
| GO:0044403 | biological process involved in symbiotic interaction | 0.050909 | 5.24E-06 | 0.000178 | 0.000138 |

**Table S7.** Top 100 significantly differentially expressed genes identified by pseudobulk analysis comparing GA and control groups across the bone defect-associated neutrophils.

| names | baseMean | log2FoldChange | lfcSE | stat | pvalue | padj |
| --- | --- | --- | --- | --- | --- | --- |
| Wfdc17 | 2501.646 | 4.144656 | 0.141074 | 29.37936 | 1.01E-189 | 6.03E-186 |
| Wfdc18 | 4.94076 | 4.031833 | 1.022587 | 3.942779 | 8.05E-05 | 0.001359122 |
| Ccl5 | 18.75811 | 3.587134 | 1.087673 | 3.297989 | 0.000973798 | 0.010846877 |
| Lipg | 19.51851 | 3.247289 | 0.416117 | 7.803788 | 6.01E-15 | 6.47E-13 |
| Ifitm1 | 2912.223 | 2.727823 | 0.070463 | 38.71295 | 0 | 0 |
| Ggt1 | 35.51268 | 2.639573 | 0.303098 | 8.708647 | 3.08E-18 | 4.84E-16 |
| Ly9 | 3.343112 | 2.399644 | 0.794721 | 3.01948 | 0.002532088 | 0.023140848 |
| E230025N22Rik | 3.250032 | 2.373165 | 0.79696 | 2.977771 | 0.002903526 | 0.025653529 |
| Nos2 | 4.891571 | 2.361884 | 0.718516 | 3.287169 | 0.001011999 | 0.011178712 |
| Lrrc75b | 5.447741 | 2.29335 | 0.59901 | 3.828569 | 0.00012889 | 0.002058632 |
| Gnb5 | 12.2529 | 2.256581 | 0.417271 | 5.407957 | 6.37E-08 | 2.38E-06 |
| Steap4 | 93.34635 | 2.212985 | 0.202866 | 10.90862 | 1.05E-27 | 3.48E-25 |
| Tlr1 | 11.10748 | 1.977415 | 0.382961 | 5.163489 | 2.42E-07 | 7.94E-06 |
| Soat2 | 8.9728 | 1.808619 | 0.409016 | 4.421881 | 9.78E-06 | 0.000218381 |
| BC035044 | 8.552538 | 1.757222 | 0.580761 | 3.02572 | 0.002480416 | 0.02275553 |
| Tgm1 | 19.08963 | 1.744834 | 0.277215 | 6.294146 | 3.09E-10 | 1.71E-08 |
| Cd38 | 19.57301 | 1.732351 | 0.326606 | 5.304095 | 1.13E-07 | 4.03E-06 |
| Cmah | 386.8345 | 1.700354 | 0.078412 | 21.68487 | 2.85E-104 | 8.53E-101 |
| A530046M15Rik | 15.88809 | 1.644315 | 0.305259 | 5.386625 | 7.18E-08 | 2.67E-06 |
| Gm7457 | 6.493458 | 1.609331 | 0.494853 | 3.25214 | 0.001145394 | 0.012322258 |
| Pkib | 6.272709 | 1.566212 | 0.556018 | 2.816839 | 0.004849885 | 0.038576579 |
| Prok2 | 104.4834 | 1.542582 | 0.15603 | 9.88642 | 4.77E-23 | 1.24E-20 |
| P2ry12 | 31.31242 | 1.521894 | 0.24093 | 6.316748 | 2.67E-10 | 1.49E-08 |
| Gm11342 | 39.05276 | 1.443155 | 0.191757 | 7.525973 | 5.23E-14 | 4.85E-12 |
| Igfbp6 | 51.13819 | 1.416096 | 0.17406 | 8.135689 | 4.10E-16 | 4.76E-14 |
| F13a1 | 27.60476 | 1.39802 | 0.484003 | 2.888455 | 0.003871396 | 0.032341835 |
| Phgdh | 20.82889 | 1.373861 | 0.351022 | 3.913891 | 9.08E-05 | 0.001504827 |
| Lrg1 | 1394.17 | 1.303876 | 0.063193 | 20.6331 | 1.38E-94 | 2.76E-91 |
| Sqle | 10.47012 | 1.274716 | 0.419838 | 3.036208 | 0.002395738 | 0.022148538 |
| Upp1 | 190.7985 | 1.234973 | 0.192067 | 6.429917 | 1.28E-10 | 7.56E-09 |
| P2ry1 | 8.652107 | 1.175545 | 0.40185 | 2.925336 | 0.003440842 | 0.029444054 |
| Ccnjl | 14.15684 | 1.164593 | 0.316742 | 3.676787 | 0.00023619 | 0.003424903 |
| Cd244a | 53.82923 | 1.16202 | 0.199239 | 5.832302 | 5.47E-09 | 2.50E-07 |
| Taco1os | 12.42498 | 1.158304 | 0.31478 | 3.679728 | 0.000233482 | 0.003397993 |
| Cdc25b | 76.95968 | 1.152299 | 0.137375 | 8.387972 | 4.95E-17 | 6.50E-15 |
| Fancg | 9.753372 | 1.149569 | 0.353784 | 3.249356 | 0.001156666 | 0.012404881 |
| 1700010I14Rik | 11.21877 | 1.105035 | 0.321669 | 3.435319 | 0.000591857 | 0.00727686 |
| Bend4 | 102.1621 | 1.089361 | 0.163022 | 6.682279 | 2.35E-11 | 1.55E-09 |
| Gbp2 | 38.11641 | 1.086653 | 0.232345 | 4.676896 | 2.91E-06 | 7.54E-05 |
| 4933412O06Rik | 31.04855 | 1.078831 | 0.209653 | 5.145794 | 2.66E-07 | 8.59E-06 |
| Sdf2l1 | 85.12113 | 1.073411 | 0.254195 | 4.222782 | 2.41E-05 | 0.000493458 |
| Rab23 | 75.96218 | 1.073258 | 0.152492 | 7.038137 | 1.95E-12 | 1.48E-10 |

| Apoc2 | 51.1622 | 1.059185 | 0.197348 | 5.367087 | 8.00E-08 | 2.94E-06 |
| --- | --- | --- | --- | --- | --- | --- |
| Mcam | 24.43064 | 1.050952 | 0.220506 | 4.766099 | 1.88E-06 | 5.10E-05 |
| Klra17 | 110.272 | 1.03982 | 0.127695 | 8.143024 | 3.86E-16 | 4.52E-14 |
| Cxcr1 | 14.88504 | 1.032461 | 0.308298 | 3.348901 | 0.000811327 | 0.009404948 |
| Srgap1 | 35.70076 | 1.021141 | 0.233131 | 4.380125 | 1.19E-05 | 0.000259406 |
| Fabp5 | 179.3884 | 1.015307 | 0.107947 | 9.405573 | 5.17E-21 | 1.09E-18 |
| Gm29264 | 41.83157 | 0.998908 | 0.186036 | 5.369421 | 7.90E-08 | 2.92E-06 |
| Serpini1 | 12.19863 | 0.991196 | 0.35643 | 2.780902 | 0.005420804 | 0.042269915 |
| Igha | 13.4468 | 0.987812 | 0.284782 | 3.468657 | 0.000523067 | 0.006579862 |
| Ccl8 | 9.104246 | 0.98634 | 0.351098 | 2.809304 | 0.00496488 | 0.039256354 |
| Galnt6 | 12.99027 | 0.980055 | 0.291225 | 3.365285 | 0.000764647 | 0.008941807 |
| Ctnnbip1 | 124.9252 | 0.966122 | 0.107097 | 9.020971 | 1.86E-19 | 3.28E-17 |
| Vsig10l | 18.7371 | 0.965656 | 0.273183 | 3.534826 | 0.000408044 | 0.005358317 |
| Idi1 | 71.06825 | 0.964141 | 0.162922 | 5.917821 | 3.26E-09 | 1.59E-07 |
| Prr5l | 75.98802 | 0.960956 | 0.169271 | 5.67702 | 1.37E-08 | 5.71E-07 |
| Tspo | 1824.457 | 0.956731 | 0.045116 | 21.20624 | 8.36E-100 | 2.00E-96 |
| Ctsc | 98.39321 | 0.954621 | 0.180503 | 5.28866 | 1.23E-07 | 4.32E-06 |
| Fdps | 134.5283 | 0.920381 | 0.179663 | 5.122828 | 3.01E-07 | 9.65E-06 |
| Pdia4 | 29.54059 | 0.901886 | 0.31499 | 2.863219 | 0.004193605 | 0.034267827 |
| Csf2rb2 | 49.10852 | 0.899073 | 0.170364 | 5.277356 | 1.31E-07 | 4.54E-06 |
| Tarm1 | 342.233 | 0.887354 | 0.095762 | 9.266239 | 1.93E-20 | 3.78E-18 |
| Tmem268 | 12.42914 | 0.883379 | 0.325455 | 2.714291 | 0.006641783 | 0.048895788 |
| Cdk5rap1 | 14.28231 | 0.876394 | 0.273804 | 3.200808 | 0.001370428 | 0.014184699 |
| Jak3 | 19.09997 | 0.862693 | 0.244029 | 3.535213 | 0.000407446 | 0.005356346 |
| Ifitm2 | 3254.623 | 0.849406 | 0.043946 | 19.32846 | 3.09E-83 | 5.29E-80 |
| S100a6 | 21598.59 | 0.836571 | 0.053682 | 15.58385 | 9.37E-55 | 9.34E-52 |
| Ppp1r16b | 85.33343 | 0.833052 | 0.141163 | 5.901335 | 3.61E-09 | 1.73E-07 |
| Gm26756 | 53.58148 | 0.814552 | 0.218391 | 3.729789 | 0.00019164 | 0.002880143 |
| Asprv1 | 624.483 | 0.812528 | 0.098422 | 8.255525 | 1.51E-16 | 1.85E-14 |
| Ufsp2 | 108.9875 | 0.810466 | 0.123348 | 6.570551 | 5.01E-11 | 3.19E-09 |
| Mov10 | 159.2577 | 0.804847 | 0.10659 | 7.550896 | 4.32E-14 | 4.04E-12 |
| Il13ra1 | 211.0437 | 0.803331 | 0.096278 | 8.343859 | 7.19E-17 | 9.05E-15 |
| Eid1 | 256.0177 | 0.7941 | 0.068905 | 11.52451 | 9.93E-31 | 5.40E-28 |
| Ifitm3 | 3590.414 | 0.787776 | 0.066981 | 11.76119 | 6.19E-32 | 3.52E-29 |
| Rufy4 | 40.87759 | 0.78608 | 0.179179 | 4.387126 | 1.15E-05 | 0.000252119 |
| Scamp1 | 131.1727 | 0.767267 | 0.120149 | 6.385978 | 1.70E-10 | 9.85E-09 |
| Sema6b | 18.61415 | 0.765925 | 0.241598 | 3.170248 | 0.001523088 | 0.015506982 |
| Fdft1 | 30.78306 | 0.765331 | 0.189706 | 4.034308 | 5.48E-05 | 0.000988136 |
| Slc28a2 | 108.0181 | 0.762931 | 0.125386 | 6.08465 | 1.17E-09 | 5.99E-08 |
| Ifitm6 | 6763.408 | 0.755925 | 0.069581 | 10.86399 | 1.71E-27 | 5.53E-25 |
| Chil5 | 104.4783 | 0.754024 | 0.119604 | 6.30433 | 2.89E-10 | 1.61E-08 |
| Ldlr | 128.4584 | 0.748823 | 0.147299 | 5.083683 | 3.70E-07 | 1.16E-05 |
| Acvrl1 | 366.8684 | 0.745258 | 0.095295 | 7.820533 | 5.26E-15 | 5.72E-13 |
| Dhx57 | 19.52151 | 0.744881 | 0.25297 | 2.944543 | 0.003234322 | 0.028056012 |
| Rab24 | 257.7018 | 0.739517 | 0.07861 | 9.407474 | 5.08E-21 | 1.09E-18 |
| Gk | 510.9671 | 0.736332 | 0.099545 | 7.396955 | 1.39E-13 | 1.22E-11 |

| Cstdc5 | 184.7556 | 0.735701 | 0.223374 | 3.293586 | 0.00098918 | 0.010997728 |
| --- | --- | --- | --- | --- | --- | --- |
| Creld2 | 50.98087 | 0.731722 | 0.160888 | 4.548028 | 5.42E-06 | 0.000129045 |
| Mif | 146.5286 | 0.717785 | 0.125748 | 5.708139 | 1.14E-08 | 4.86E-07 |
| Bst1 | 492.2696 | 0.705735 | 0.063754 | 11.06972 | 1.76E-28 | 6.79E-26 |
| Ptges2 | 25.10125 | 0.704287 | 0.201438 | 3.496303 | 0.000471753 | 0.006035913 |
| Slc22a20 | 32.45879 | 0.679376 | 0.196023 | 3.465795 | 0.000528667 | 0.00662245 |
| Cenpb | 58.14968 | 0.676116 | 0.228549 | 2.958299 | 0.003093415 | 0.027031796 |
| Prss16 | 21.00382 | 0.674175 | 0.245139 | 2.750169 | 0.005956452 | 0.045242559 |
| Gm11290 | 59.05025 | 0.670744 | 0.231743 | 2.894347 | 0.003799483 | 0.031852289 |
| Pdia6 | 239.1766 | 0.670382 | 0.184547 | 3.632575 | 0.000280607 | 0.003926204 |
| AI987944 | 18.51765 | 0.667924 | 0.242149 | 2.758315 | 0.005810011 | 0.044412244 |
| Rhoc | 73.88121 | 0.664116 | 0.120614 | 5.506142 | 3.67E-08 | 1.43E-06 |

**Table S8.** Top 100 significantly enriched Gene Ontology (GO) terms in GA compared to control groups identified by scRNA-seq analysis of the bone defect-associated neutrophils.

| **ID** | **Description** | **RichFactor** | **pvalue** | **p.adjust** | **qvalue** |
| --- | --- | --- | --- | --- | --- |
| GO:0034341 | response to type II interferon | 0.027972 | 8.66E-05 | 0.023773 | 0.015718 |
| GO:0032757 | positive regulation of interleukin-8 production | 0.04918 | 0.000135 | 0.023773 | 0.015718 |
| GO:0034384 | high-density lipoprotein particle clearance | 0.181818 | 0.000141 | 0.023773 | 0.015718 |
| GO:0015850 | organic hydroxy compound transport | 0.015873 | 0.000156 | 0.023773 | 0.015718 |
| GO:0046394 | carboxylic acid biosynthetic process | 0.015244 | 0.000189 | 0.023773 | 0.015718 |
| GO:0001973 | G protein-coupled adenosine receptor signaling pathway | 0.153846 | 0.000199 | 0.023773 | 0.015718 |
| GO:0035588 | G protein-coupled purinergic receptor signaling pathway | 0.153846 | 0.000199 | 0.023773 | 0.015718 |
| GO:0016053 | organic acid biosynthetic process | 0.01506 | 0.0002 | 0.023773 | 0.015718 |
| GO:0009064 | glutamine family amino acid metabolic process | 0.042857 | 0.000204 | 0.023773 | 0.015718 |
| GO:0097006 | regulation of plasma lipoprotein particle levels | 0.042857 | 0.000204 | 0.023773 | 0.015718 |
| GO:0009070 | serine family amino acid biosynthetic process | 0.133333 | 0.000268 | 0.023773 | 0.015718 |
| GO:0032677 | regulation of interleukin-8 production | 0.038462 | 0.000281 | 0.023773 | 0.015718 |
| GO:0032637 | interleukin-8 production | 0.037975 | 0.000291 | 0.023773 | 0.015718 |
| GO:0010838 | positive regulation of keratinocyte proliferation | 0.125 | 0.000306 | 0.023773 | 0.015718 |
| GO:0060732 | positive regulation of inositol phosphate biosynthetic process | 0.125 | 0.000306 | 0.023773 | 0.015718 |
| GO:0010919 | regulation of inositol phosphate biosynthetic process | 0.111111 | 0.000389 | 0.026903 | 0.017787 |
| GO:0010518 | positive regulation of phospholipase activity | 0.105263 | 0.000434 | 0.026903 | 0.017787 |
| GO:0033604 | negative regulation of catecholamine secretion | 0.105263 | 0.000434 | 0.026903 | 0.017787 |
| GO:0042060 | wound healing | 0.01269 | 0.000438 | 0.026903 | 0.017787 |
| GO:0014061 | regulation of norepinephrine secretion | 0.1 | 0.000482 | 0.028114 | 0.018588 |
| GO:0048243 | norepinephrine secretion | 0.090909 | 0.000585 | 0.032486 | 0.021478 |
| GO:0010984 | regulation of lipoprotein particle clearance | 0.086957 | 0.00064 | 0.033927 | 0.022431 |
| GO:0010517 | regulation of phospholipase activity | 0.08 | 0.000757 | 0.038401 | 0.02539 |
| GO:0033630 | positive regulation of cell adhesion mediated by integrin | 0.076923 | 0.000819 | 0.038718 | 0.025599 |
| GO:0006869 | lipid transport | 0.010965 | 0.000847 | 0.038718 | 0.025599 |
| GO:0035590 | purinergic nucleotide receptor signaling pathway | 0.074074 | 0.000884 | 0.038718 | 0.025599 |
| GO:0030301 | cholesterol transport | 0.025862 | 0.000896 | 0.038718 | 0.025599 |
| GO:0071346 | cellular response to type II interferon | 0.025424 | 0.000941 | 0.039228 | 0.025936 |
| GO:0015918 | sterol transport | 0.025 | 0.000988 | 0.039761 | 0.026288 |
| GO:0019433 | triglyceride catabolic process | 0.066667 | 0.001092 | 0.040016 | 0.026457 |
| GO:0060193 | positive regulation of lipase activity | 0.066667 | 0.001092 | 0.040016 | 0.026457 |
| GO:0018149 | peptide cross-linking | 0.064516 | 0.001166 | 0.040016 | 0.026457 |
| GO:0032958 | inositol phosphate biosynthetic process | 0.064516 | 0.001166 | 0.040016 | 0.026457 |
| GO:1905523 | positive regulation of macrophage migration | 0.064516 | 0.001166 | 0.040016 | 0.026457 |
| GO:0015874 | norepinephrine transport | 0.0625 | 0.001242 | 0.041421 | 0.027386 |
| GO:0009069 | serine family amino acid metabolic process | 0.058824 | 0.001402 | 0.045454 | 0.030052 |
| GO:1902932 | positive regulation of alcohol biosynthetic process | 0.057143 | 0.001486 | 0.046857 | 0.03098 |
| GO:0034381 | plasma lipoprotein particle clearance | 0.051282 | 0.001843 | 0.055133 | 0.036452 |
| GO:0051953 | negative regulation of amine transport | 0.051282 | 0.001843 | 0.055133 | 0.036452 |
| GO:0050671 | positive regulation of lymphocyte proliferation | 0.019231 | 0.002096 | 0.057923 | 0.038297 |
| GO:0046461 | neutral lipid catabolic process | 0.047619 | 0.002134 | 0.057923 | 0.038297 |
| GO:0046464 | acylglycerol catabolic process | 0.047619 | 0.002134 | 0.057923 | 0.038297 |

| GO:0071827 | plasma lipoprotein particle organization | 0.047619 | 0.002134 | 0.057923 | 0.038297 |
| --- | --- | --- | --- | --- | --- |
| GO:0032946 | positive regulation of mononuclear cell proliferation | 0.018868 | 0.002213 | 0.057988 | 0.03834 |
| GO:0043647 | inositol phosphate metabolic process | 0.046512 | 0.002236 | 0.057988 | 0.03834 |
| GO:0006633 | fatty acid biosynthetic process | 0.018293 | 0.002416 | 0.060648 | 0.040098 |
| GO:0046883 | regulation of hormone secretion | 0.011494 | 0.002443 | 0.060648 | 0.040098 |
| GO:0071825 | protein-lipid complex organization | 0.043478 | 0.002555 | 0.062118 | 0.04107 |
| GO:0170039 | proteinogenic amino acid metabolic process | 0.017751 | 0.00263 | 0.062638 | 0.041414 |
| GO:1905521 | regulation of macrophage migration | 0.040816 | 0.002894 | 0.06641 | 0.043908 |
| GO:0070665 | positive regulation of leukocyte proliferation | 0.017143 | 0.002902 | 0.06641 | 0.043908 |
| GO:0006066 | alcohol metabolic process | 0.010753 | 0.003104 | 0.068947 | 0.045585 |
| GO:0010837 | regulation of keratinocyte proliferation | 0.039216 | 0.003131 | 0.068947 | 0.045585 |
| GO:0033628 | regulation of cell adhesion mediated by integrin | 0.038462 | 0.003253 | 0.069026 | 0.045637 |
| GO:0042304 | regulation of fatty acid biosynthetic process | 0.038462 | 0.003253 | 0.069026 | 0.045637 |
| GO:0170033 | L-amino acid metabolic process | 0.016043 | 0.003497 | 0.072498 | 0.047933 |
| GO:0007596 | blood coagulation | 0.015957 | 0.00355 | 0.072498 | 0.047933 |
| GO:0055088 | lipid homeostasis | 0.015873 | 0.003603 | 0.072498 | 0.047933 |
| GO:0007599 | hemostasis | 0.015625 | 0.003766 | 0.074314 | 0.049134 |
| GO:0050817 | coagulation | 0.015544 | 0.003821 | 0.074314 | 0.049134 |
| GO:0050433 | regulation of catecholamine secretion | 0.035088 | 0.003895 | 0.074523 | 0.049272 |
| GO:0170038 | proteinogenic amino acid biosynthetic process | 0.034483 | 0.00403 | 0.075582 | 0.049972 |
| GO:0170034 | L-amino acid biosynthetic process | 0.033898 | 0.004167 | 0.075582 | 0.049972 |
| GO:0062012 | regulation of small molecule metabolic process | 0.009877 | 0.0042 | 0.075582 | 0.049972 |
| GO:0007204 | positive regulation of cytosolic calcium ion concentration | 0.015 | 0.004221 | 0.075582 | 0.049972 |
| GO:1902930 | regulation of alcohol biosynthetic process | 0.033333 | 0.004307 | 0.075582 | 0.049972 |
| GO:0062013 | positive regulation of small molecule metabolic process | 0.014851 | 0.004339 | 0.075582 | 0.049972 |
| GO:0042742 | defense response to bacterium | 0.009685 | 0.0045 | 0.07723 | 0.051062 |
| GO:0046879 | hormone secretion | 0.009639 | 0.004577 | 0.077419 | 0.051187 |
| GO:0019730 | antimicrobial humoral response | 0.014286 | 0.004834 | 0.078209 | 0.051709 |
| GO:1901607 | alpha-amino acid biosynthetic process | 0.03125 | 0.004885 | 0.078209 | 0.051709 |
| GO:0009914 | hormone transport | 0.009412 | 0.004978 | 0.078209 | 0.051709 |
| GO:0043616 | keratinocyte proliferation | 0.030769 | 0.005035 | 0.078209 | 0.051709 |
| GO:0045071 | negative regulation of viral genome replication | 0.030769 | 0.005035 | 0.078209 | 0.051709 |
| GO:0046173 | polyol biosynthetic process | 0.030769 | 0.005035 | 0.078209 | 0.051709 |
| GO:0032371 | regulation of sterol transport | 0.030303 | 0.005187 | 0.078209 | 0.051709 |
| GO:0032374 | regulation of cholesterol transport | 0.030303 | 0.005187 | 0.078209 | 0.051709 |
| GO:1901605 | alpha-amino acid metabolic process | 0.013889 | 0.005227 | 0.078209 | 0.051709 |
| GO:0033344 | cholesterol efflux | 0.029412 | 0.005497 | 0.080191 | 0.05302 |
| GO:0046503 | glycerolipid catabolic process | 0.029412 | 0.005497 | 0.080191 | 0.05302 |
| GO:0050432 | catecholamine secretion | 0.028986 | 0.005656 | 0.080488 | 0.053216 |
| GO:1905517 | macrophage migration | 0.028986 | 0.005656 | 0.080488 | 0.053216 |
| GO:0072330 | monocarboxylic acid biosynthetic process | 0.013274 | 0.005924 | 0.083292 | 0.05507 |
| GO:0034612 | response to tumor necrosis factor | 0.012987 | 0.006292 | 0.086404 | 0.057127 |
| GO:0008652 | amino acid biosynthetic process | 0.027397 | 0.006309 | 0.086404 | 0.057127 |
| GO:0050796 | regulation of insulin secretion | 0.012931 | 0.006367 | 0.086404 | 0.057127 |
| GO:0008347 | glial cell migration | 0.027027 | 0.006478 | 0.086893 | 0.05745 |
| GO:0035456 | response to interferon-beta | 0.025974 | 0.006996 | 0.09166 | 0.060602 |

| GO:0062208 | positive regulation of pattern recognition receptor signaling pathway | 0.025974 | 0.006996 | 0.09166 | 0.060602 |
| --- | --- | --- | --- | --- | --- |
| GO:0050679 | positive regulation of epithelial cell proliferation | 0.012448 | 0.007069 | 0.09166 | 0.060602 |
| GO:0006935 | chemotaxis | 0.008316 | 0.007659 | 0.098215 | 0.064936 |
| GO:0042330 | taxis | 0.008282 | 0.007769 | 0.098547 | 0.065156 |
| GO:0043270 | positive regulation of monoatomic ion transport | 0.011858 | 0.008073 | 0.101306 | 0.06698 |
| GO:0003018 | vascular process in circulatory system | 0.011719 | 0.008337 | 0.101849 | 0.067339 |
| GO:0097529 | myeloid leukocyte migration | 0.011719 | 0.008337 | 0.101849 | 0.067339 |
| GO:0002237 | response to molecule of bacterial origin | 0.008097 | 0.008394 | 0.101849 | 0.067339 |
| GO:0070098 | chemokine-mediated signaling pathway | 0.023529 | 0.008466 | 0.101849 | 0.067339 |
| GO:0050670 | regulation of lymphocyte proliferation | 0.011583 | 0.008605 | 0.102474 | 0.067752 |
| GO:0007193 | adenylate cyclase-inhibiting G protein-coupled receptor signaling  pathway | 0.022989 | 0.008853 | 0.103313 | 0.068307 |
| GO:0051937 | catecholamine transport | 0.022989 | 0.008853 | 0.103313 | 0.068307 |

**Table S9.** Top 100 significantly differentially expressed genes identified by pseudobulk analysis comparing GA and control groups across the bone defect-associated macrophages.

| names | baseMean | log2FoldChange | lfcSE | stat | pvalue | padj |
| --- | --- | --- | --- | --- | --- | --- |
| Ccm2l | 1.411648 | 4.035733 | 1.604913 | 2.514612 | 0.011916338 | 0.034554877 |
| Cd200r3 | 3.055188 | 3.607255 | 1.402939 | 2.571214 | 0.010134283 | 0.030052708 |
| Nxf2 | 1.472066 | 3.545919 | 1.300452 | 2.726682 | 0.006397457 | 0.02021456 |
| Hoxa7 | 2.666744 | 3.385785 | 0.966974 | 3.501424 | 0.000462779 | 0.001936788 |
| Akr1c18 | 7.94363 | 3.376268 | 0.533525 | 6.328232 | 2.48E-10 | 2.69E-09 |
| BB557941 | 3.187759 | 3.231696 | 1.244992 | 2.595756 | 0.009438316 | 0.028333967 |
| Chac1 | 9.337326 | 3.032265 | 0.602016 | 5.036848 | 4.73E-07 | 3.34E-06 |
| Gpld1 | 7.103236 | 3.017421 | 0.564073 | 5.349345 | 8.83E-08 | 6.85E-07 |
| Olfr655 | 3.48902 | 3.005488 | 0.976212 | 3.078725 | 0.002078888 | 0.007498212 |
| Gm34680 | 11.66497 | 2.952567 | 0.769237 | 3.838308 | 0.000123885 | 0.000579989 |
| Gm12709 | 2.674678 | 2.925527 | 0.965567 | 3.029855 | 0.002446715 | 0.008672536 |
| Rnase2a | 3.273929 | 2.898537 | 0.728612 | 3.978161 | 6.95E-05 | 0.000343119 |
| 1700048O20Rik | 1.40006 | 2.881602 | 1.13868 | 2.530652 | 0.011385075 | 0.033250236 |
| Hoxa9 | 1.362961 | 2.865806 | 1.196406 | 2.395345 | 0.016604733 | 0.045847414 |
| Muc13 | 10.08454 | 2.822396 | 0.775336 | 3.640222 | 0.000272403 | 0.001196745 |
| Serpinb9b | 3.66384 | 2.776733 | 1.044947 | 2.657295 | 0.007877054 | 0.02429318 |
| Pla2g4c | 3.596949 | 2.752604 | 0.678193 | 4.05873 | 4.93E-05 | 0.00025036 |
| Lcn2 | 566.5773 | 2.697967 | 0.342168 | 7.884926 | 3.15E-15 | 5.83E-14 |
| Wfdc21 | 264.4307 | 2.546543 | 0.282107 | 9.026882 | 1.77E-19 | 5.03E-18 |
| Abca13 | 36.64441 | 2.539753 | 0.466212 | 5.447632 | 5.10E-08 | 4.12E-07 |
| Edn1 | 39.65513 | 2.527359 | 0.976781 | 2.587436 | 0.009669325 | 0.028905232 |
| Sycp2 | 2.569963 | 2.501659 | 0.977467 | 2.559329 | 0.010487453 | 0.030955001 |
| S100a8 | 6505.454 | 2.497129 | 0.330573 | 7.553946 | 4.22E-14 | 6.94E-13 |
| Mrgpra2b | 4.579493 | 2.46652 | 0.565597 | 4.360912 | 1.30E-05 | 7.26E-05 |
| Prss57 | 11.73679 | 2.462823 | 0.456412 | 5.396048 | 6.81E-08 | 5.37E-07 |
| Gpr141b | 3.556493 | 2.456171 | 0.648895 | 3.785159 | 0.000153611 | 0.000708886 |
| A830012C17Rik | 3.039387 | 2.451079 | 0.67807 | 3.614787 | 0.000300594 | 0.001304006 |
| Bicdl1 | 3.492691 | 2.448805 | 0.674181 | 3.632265 | 0.000280944 | 0.001228938 |
| Gm39121 | 5.029861 | 2.440374 | 0.538597 | 4.530981 | 5.87E-06 | 3.49E-05 |
| Hist1h2bp | 7.431199 | 2.420969 | 0.476076 | 5.085252 | 3.67E-07 | 2.64E-06 |
| Lin28a | 1.467244 | 2.398979 | 1.005466 | 2.385937 | 0.017035658 | 0.046842664 |
| Cdhr4 | 1.464646 | 2.396473 | 0.953677 | 2.512877 | 0.011975103 | 0.034690106 |
| Gm16618 | 1.453883 | 2.391976 | 0.987549 | 2.422135 | 0.015429638 | 0.043113308 |
| Tmem108 | 29.08471 | 2.373624 | 0.507781 | 4.674501 | 2.95E-06 | 1.84E-05 |
| Chil1 | 10.63655 | 2.371122 | 0.500907 | 4.733653 | 2.21E-06 | 1.40E-05 |
| Tctex1d1 | 20.98307 | 2.370338 | 0.585553 | 4.048035 | 5.16E-05 | 0.000261379 |
| Asprv1 | 10.53509 | 2.364821 | 0.639631 | 3.697167 | 0.000218019 | 0.000978476 |
| Cd7 | 21.00003 | 2.360296 | 0.51369 | 4.594783 | 4.33E-06 | 2.62E-05 |
| S100a9 | 4985.792 | 2.326074 | 0.39346 | 5.911843 | 3.38E-09 | 3.19E-08 |
| Entpd3 | 3.747664 | 2.317857 | 0.935518 | 2.477619 | 0.013226247 | 0.037876182 |
| Hist1h3h | 2.740324 | 2.291739 | 0.7163 | 3.199414 | 0.001377075 | 0.005179628 |
| Gm39321 | 1.383789 | 2.289853 | 0.956256 | 2.394602 | 0.016638425 | 0.045916612 |

| Gm16046 | 1.363889 | 2.288483 | 0.954434 | 2.397739 | 0.016496598 | 0.04564129 |
| --- | --- | --- | --- | --- | --- | --- |
| Khdc3 | 3.179961 | 2.275589 | 0.732389 | 3.107078 | 0.001889464 | 0.006873739 |
| Hist1h3g | 9.062328 | 2.275038 | 0.405178 | 5.614912 | 1.97E-08 | 1.68E-07 |
| Gm17201 | 1.780035 | 2.258656 | 0.862701 | 2.618122 | 0.008841531 | 0.026842634 |
| Hist1h2bk | 9.832894 | 2.24577 | 0.523086 | 4.293311 | 1.76E-05 | 9.65E-05 |
| Cldn15 | 19.06921 | 2.238819 | 0.436098 | 5.133749 | 2.84E-07 | 2.08E-06 |
| 4930401C15Rik | 2.670785 | 2.233242 | 0.689788 | 3.237576 | 0.001205497 | 0.004588804 |
| Tacstd2 | 7.510174 | 2.222215 | 0.454722 | 4.886977 | 1.02E-06 | 6.88E-06 |
| Teddm2 | 1.721697 | 2.174845 | 0.862691 | 2.521003 | 0.011702102 | 0.034057151 |
| Hist1h2bh | 17.64338 | 2.174371 | 0.280592 | 7.749237 | 9.24E-15 | 1.62E-13 |
| 2310047D07Rik | 2.126377 | 2.167064 | 0.791357 | 2.738414 | 0.006173626 | 0.019596684 |
| Gpr55 | 3.413917 | 2.163342 | 0.758709 | 2.851347 | 0.004353444 | 0.014405907 |
| Lrg1 | 60.45093 | 2.15851 | 0.261037 | 8.268984 | 1.35E-16 | 2.93E-15 |
| Ankrd22 | 3.866609 | 2.155456 | 0.896842 | 2.403383 | 0.016244146 | 0.045065937 |
| Hist1h2bn | 9.533881 | 2.110696 | 0.373982 | 5.643844 | 1.66E-08 | 1.44E-07 |
| Phgdh | 113.6796 | 2.096179 | 0.301155 | 6.960462 | 3.39E-12 | 4.49E-11 |
| Ly6g | 15.18319 | 2.09421 | 0.489753 | 4.276054 | 1.90E-05 | 0.000103735 |
| Trib3 | 12.6387 | 2.082693 | 0.371062 | 5.612793 | 1.99E-08 | 1.70E-07 |
| 1700026N04Rik | 1.623554 | 2.079482 | 0.87954 | 2.364283 | 0.018065012 | 0.049296088 |
| Cxcr2 | 28.61809 | 2.069936 | 0.709302 | 2.918273 | 0.003519763 | 0.012013535 |
| Asns | 22.60982 | 2.069422 | 0.37569 | 5.508331 | 3.62E-08 | 2.98E-07 |
| Ifitm1 | 259.0624 | 2.055345 | 0.219641 | 9.357753 | 8.15E-21 | 2.67E-19 |
| Ovol2 | 2.398757 | 2.047103 | 0.756319 | 2.706665 | 0.006796272 | 0.02135891 |
| Gm38575 | 7.398459 | 1.999678 | 0.466203 | 4.289282 | 1.79E-05 | 9.82E-05 |
| Cntn5 | 2.334714 | 1.987098 | 0.790009 | 2.515285 | 0.011893632 | 0.034509406 |
| G0s2 | 112.4904 | 1.970246 | 0.352433 | 5.59041 | 2.27E-08 | 1.91E-07 |
| 4930586N03Rik | 2.662646 | 1.954274 | 0.784527 | 2.491023 | 0.012737583 | 0.0366495 |
| B4galnt4 | 1.885866 | 1.943651 | 0.774816 | 2.508534 | 0.012123331 | 0.03506142 |
| Ffar2 | 3.009333 | 1.936852 | 0.673228 | 2.876964 | 0.004015211 | 0.013453702 |
| Prtn3 | 1693.541 | 1.925952 | 0.253544 | 7.596122 | 3.05E-14 | 5.09E-13 |
| Kbtbd6 | 3.315202 | 1.918599 | 0.623025 | 3.079488 | 0.002073566 | 0.007484348 |
| Il1r2 | 227.5938 | 1.909477 | 0.22538 | 8.472266 | 2.41E-17 | 5.67E-16 |
| Hist2h2bb | 21.96862 | 1.893295 | 0.268997 | 7.038363 | 1.95E-12 | 2.66E-11 |
| Retnlg | 427.9608 | 1.886917 | 0.513044 | 3.677888 | 0.000235173 | 0.001047729 |
| Gm17767 | 7.594808 | 1.866401 | 0.411615 | 4.534334 | 5.78E-06 | 3.45E-05 |
| Ticrr | 43.80681 | 1.856423 | 0.220606 | 8.415117 | 3.93E-17 | 9.03E-16 |
| B230208H11Rik | 19.25851 | 1.845669 | 0.375568 | 4.914345 | 8.91E-07 | 6.04E-06 |
| Zik1 | 2.157515 | 1.838318 | 0.739841 | 2.484746 | 0.01296439 | 0.03723861 |
| Platr21 | 2.154126 | 1.837021 | 0.760135 | 2.416704 | 0.015661753 | 0.043663082 |
| Tgm3 | 2.118213 | 1.825704 | 0.769295 | 2.373216 | 0.017633954 | 0.048286775 |
| E2f7 | 47.32887 | 1.822577 | 0.190498 | 9.567429 | 1.10E-21 | 3.84E-20 |
| Hist1h4k | 3.875023 | 1.803925 | 0.548149 | 3.290939 | 0.000998535 | 0.003871809 |
| Dmkn | 173.9935 | 1.787258 | 0.153336 | 11.65579 | 2.14E-31 | 1.85E-29 |
| Mapk13 | 15.58117 | 1.777449 | 0.353578 | 5.027032 | 4.98E-07 | 3.51E-06 |
| Hist1h1a | 63.76856 | 1.777175 | 0.161238 | 11.02206 | 2.99E-28 | 1.98E-26 |
| 1700020L24Rik | 51.69304 | 1.776333 | 0.259787 | 6.837659 | 8.05E-12 | 1.03E-10 |

| Fcnb | 92.86 | 1.775174 | 0.277148 | 6.405156 | 1.50E-10 | 1.67E-09 |
| --- | --- | --- | --- | --- | --- | --- |
| Mapk4 | 8.252346 | 1.772499 | 0.514248 | 3.446775 | 0.00056732 | 0.002321232 |
| Syn3 | 3.085676 | 1.764479 | 0.679763 | 2.595729 | 0.00943906 | 0.028333967 |
| Gm42997 | 3.708209 | 1.759349 | 0.590507 | 2.979389 | 0.002888242 | 0.01007767 |
| Hist2h3b | 8.864884 | 1.757613 | 0.361807 | 4.857873 | 1.19E-06 | 7.89E-06 |
| Fam83e | 3.058969 | 1.757559 | 0.59571 | 2.950357 | 0.003174066 | 0.010956693 |
| Chil3 | 3099.899 | 1.756216 | 0.136563 | 12.86016 | 7.54E-38 | 1.10E-35 |
| Hist1h1b | 924.6092 | 1.749996 | 0.109986 | 15.91109 | 5.31E-57 | 2.69E-54 |
| Rdh12 | 31.91069 | 1.749121 | 0.230962 | 7.573211 | 3.64E-14 | 6.04E-13 |
| Lypd6b | 23.37177 | 1.748662 | 0.306708 | 5.701393 | 1.19E-08 | 1.05E-07 |
| Il18rap | 31.15402 | 1.743306 | 0.211152 | 8.256171 | 1.50E-16 | 3.24E-15 |
| Pclaf | 959.876 | 1.739434 | 0.123429 | 14.09261 | 4.22E-45 | 1.02E-42 |

**Table S10.** Top 100 significantly enriched Gene Ontology (GO) terms in GA compared to control groups identified by scRNA-seq analysis of the bone defect-associated macrophages.

| **ID** | **Description** | **RichFactor** | **pvalue** | **p.adjust** | **qvalue** |
| --- | --- | --- | --- | --- | --- |
| GO:0007059 | chromosome segregation | 0.233256 | 1.45E-88 | 4.89E-85 | 4.10E-85 |
| GO:0000280 | nuclear division | 0.202991 | 5.26E-77 | 8.86E-74 | 7.43E-74 |
| GO:0098813 | nuclear chromosome segregation | 0.253049 | 1.43E-75 | 1.61E-72 | 1.35E-72 |
| GO:0000070 | mitotic sister chromatid segregation | 0.321053 | 1.83E-62 | 1.23E-59 | 1.04E-59 |
| GO:0000819 | sister chromatid segregation | 0.282609 | 1.83E-62 | 1.23E-59 | 1.04E-59 |
| GO:0140014 | mitotic nuclear division | 0.241135 | 3.47E-60 | 1.95E-57 | 1.63E-57 |
| GO:0010948 | negative regulation of cell cycle process | 0.187879 | 9.59E-48 | 4.62E-45 | 3.87E-45 |
| GO:0090068 | positive regulation of cell cycle process | 0.210145 | 1.29E-47 | 5.45E-45 | 4.57E-45 |
| GO:0045787 | positive regulation of cell cycle | 0.175 | 1.60E-46 | 6.00E-44 | 5.03E-44 |
| GO:1901987 | regulation of cell cycle phase transition | 0.147368 | 1.81E-46 | 6.11E-44 | 5.12E-44 |
| GO:1903046 | meiotic cell cycle process | 0.220884 | 2.00E-46 | 6.12E-44 | 5.13E-44 |
| GO:0044772 | mitotic cell cycle phase transition | 0.149451 | 1.53E-45 | 4.30E-43 | 3.61E-43 |
| GO:0000075 | cell cycle checkpoint signaling | 0.229358 | 4.05E-43 | 1.05E-40 | 8.81E-41 |
| GO:0006260 | DNA replication | 0.197802 | 7.29E-43 | 1.76E-40 | 1.47E-40 |
| GO:0045786 | negative regulation of cell cycle | 0.152542 | 9.69E-43 | 2.18E-40 | 1.83E-40 |
| GO:0051321 | meiotic cell cycle | 0.163435 | 8.44E-42 | 1.78E-39 | 1.49E-39 |
| GO:1901988 | negative regulation of cell cycle phase transition | 0.188811 | 9.86E-42 | 1.96E-39 | 1.64E-39 |
| GO:0051983 | regulation of chromosome segregation | 0.30597 | 3.71E-41 | 6.95E-39 | 5.83E-39 |
| GO:0006261 | DNA-templated DNA replication | 0.275641 | 6.31E-41 | 1.12E-38 | 9.38E-39 |
| GO:0140013 | meiotic nuclear division | 0.218182 | 2.62E-40 | 4.42E-38 | 3.71E-38 |
| GO:1901990 | regulation of mitotic cell cycle phase transition | 0.161017 | 5.07E-40 | 8.13E-38 | 6.82E-38 |
| GO:0007093 | mitotic cell cycle checkpoint signaling | 0.2625 | 5.24E-39 | 8.03E-37 | 6.74E-37 |
| GO:0051310 | metaphase chromosome alignment | 0.343137 | 2.73E-37 | 4.01E-35 | 3.36E-35 |
| GO:1905818 | regulation of chromosome separation | 0.410256 | 3.53E-37 | 4.96E-35 | 4.16E-35 |
| GO:0051304 | chromosome separation | 0.375 | 9.86E-37 | 1.33E-34 | 1.11E-34 |
| GO:0031577 | spindle checkpoint signaling | 0.491525 | 1.03E-36 | 1.33E-34 | 1.12E-34 |
| GO:0007051 | spindle organization | 0.210784 | 1.66E-35 | 2.07E-33 | 1.74E-33 |
| GO:0051303 | establishment of chromosome localization | 0.299145 | 6.98E-35 | 8.40E-33 | 7.05E-33 |
| GO:0071174 | mitotic spindle checkpoint signaling | 0.490909 | 3.06E-34 | 3.56E-32 | 2.98E-32 |
| GO:0045930 | negative regulation of mitotic cell cycle | 0.180723 | 5.31E-34 | 5.97E-32 | 5.00E-32 |
| GO:0050000 | chromosome localization | 0.282258 | 6.90E-34 | 7.50E-32 | 6.29E-32 |
| GO:0045839 | negative regulation of mitotic nuclear division | 0.430769 | 2.06E-33 | 2.17E-31 | 1.82E-31 |
| GO:0033044 | regulation of chromosome organization | 0.175097 | 2.26E-33 | 2.31E-31 | 1.94E-31 |
| GO:0051985 | negative regulation of chromosome segregation | 0.457627 | 3.66E-33 | 3.62E-31 | 3.04E-31 |
| GO:1901991 | negative regulation of mitotic cell cycle phase transition | 0.209424 | 5.68E-33 | 5.47E-31 | 4.59E-31 |
| GO:0051784 | negative regulation of nuclear division | 0.376623 | 1.77E-32 | 1.58E-30 | 1.32E-30 |
| GO:0033046 | negative regulation of sister chromatid segregation | 0.472727 | 1.87E-32 | 1.58E-30 | 1.32E-30 |
| GO:0033048 | negative regulation of mitotic sister chromatid segregation | 0.472727 | 1.87E-32 | 1.58E-30 | 1.32E-30 |
| GO:0045841 | negative regulation of mitotic metaphase/anaphase  transition | 0.472727 | 1.87E-32 | 1.58E-30 | 1.32E-30 |
| GO:2000816 | negative regulation of mitotic sister chromatid separation | 0.472727 | 1.87E-32 | 1.58E-30 | 1.32E-30 |
| GO:0033047 | regulation of mitotic sister chromatid segregation | 0.435484 | 2.02E-32 | 1.66E-30 | 1.39E-30 |

| GO:0007088 | regulation of mitotic nuclear division | 0.272 | 2.43E-32 | 1.95E-30 | 1.63E-30 |
| --- | --- | --- | --- | --- | --- |
| GO:0051783 | regulation of nuclear division | 0.229814 | 3.73E-32 | 2.92E-30 | 2.45E-30 |
| GO:0010965 | regulation of mitotic sister chromatid separation | 0.421875 | 5.95E-32 | 4.55E-30 | 3.82E-30 |
| GO:1902100 | negative regulation of metaphase/anaphase transition of  cell cycle | 0.45614 | 6.25E-32 | 4.58E-30 | 3.84E-30 |
| GO:1905819 | negative regulation of chromosome separation | 0.45614 | 6.25E-32 | 4.58E-30 | 3.84E-30 |
| GO:1902850 | microtubule cytoskeleton organization involved in mitosis | 0.22561 | 7.74E-32 | 5.55E-30 | 4.65E-30 |
| GO:0033045 | regulation of sister chromatid segregation | 0.293578 | 1.09E-31 | 7.65E-30 | 6.42E-30 |
| GO:0051306 | mitotic sister chromatid separation | 0.402985 | 2.76E-31 | 1.90E-29 | 1.59E-29 |
| GO:0007094 | mitotic spindle assembly checkpoint signaling | 0.471698 | 3.21E-31 | 2.12E-29 | 1.78E-29 |
| GO:0071173 | spindle assembly checkpoint signaling | 0.471698 | 3.21E-31 | 2.12E-29 | 1.78E-29 |
| GO:0007091 | metaphase/anaphase transition of mitotic cell cycle | 0.306122 | 2.13E-30 | 1.38E-28 | 1.16E-28 |
| GO:0007052 | mitotic spindle organization | 0.251908 | 3.26E-30 | 2.07E-28 | 1.74E-28 |
| GO:0008608 | attachment of spindle microtubules to kinetochore | 0.470588 | 5.50E-30 | 3.43E-28 | 2.88E-28 |
| GO:0044784 | metaphase/anaphase transition of cell cycle | 0.29703 | 5.96E-30 | 3.65E-28 | 3.06E-28 |
| GO:0045132 | meiotic chromosome segregation | 0.275229 | 7.73E-29 | 4.65E-27 | 3.90E-27 |
| GO:2001251 | negative regulation of chromosome organization | 0.284314 | 2.26E-28 | 1.34E-26 | 1.12E-26 |
| GO:0030071 | regulation of mitotic metaphase/anaphase transition | 0.301075 | 3.22E-28 | 1.87E-26 | 1.57E-26 |
| GO:0006310 | DNA recombination | 0.131965 | 6.65E-28 | 3.80E-26 | 3.18E-26 |
| GO:1902099 | regulation of metaphase/anaphase transition of cell cycle | 0.291667 | 8.78E-28 | 4.93E-26 | 4.14E-26 |
| GO:0061982 | meiosis I cell cycle process | 0.219178 | 2.96E-27 | 1.64E-25 | 1.37E-25 |
| GO:0051225 | spindle assembly | 0.22963 | 4.18E-27 | 2.27E-25 | 1.90E-25 |
| GO:0044839 | cell cycle G2/M phase transition | 0.198758 | 7.88E-26 | 4.22E-24 | 3.54E-24 |
| GO:0007127 | meiosis I | 0.208633 | 3.72E-24 | 1.96E-22 | 1.64E-22 |
| GO:1901989 | positive regulation of cell cycle phase transition | 0.219512 | 3.35E-23 | 1.74E-21 | 1.46E-21 |
| GO:0000086 | G2/M transition of mitotic cell cycle | 0.195804 | 1.46E-22 | 7.47E-21 | 6.26E-21 |
| GO:0010639 | negative regulation of organelle organization | 0.10687 | 1.77E-22 | 8.92E-21 | 7.48E-21 |
| GO:0140694 | membraneless organelle assembly | 0.098851 | 1.17E-21 | 5.81E-20 | 4.87E-20 |
| GO:0006302 | double-strand break repair | 0.11859 | 1.75E-21 | 8.57E-20 | 7.18E-20 |
| GO:0007080 | mitotic metaphase chromosome alignment | 0.31746 | 4.88E-21 | 2.35E-19 | 1.97E-19 |
| GO:0000725 | recombinational repair | 0.15847 | 1.27E-20 | 6.04E-19 | 5.06E-19 |
| GO:0051656 | establishment of organelle localization | 0.088 | 3.77E-20 | 1.76E-18 | 1.48E-18 |
| GO:0000724 | double-strand break repair via homologous recombination | 0.157303 | 7.28E-20 | 3.36E-18 | 2.82E-18 |
| GO:1901976 | regulation of cell cycle checkpoint | 0.339623 | 1.13E-19 | 5.15E-18 | 4.32E-18 |
| GO:0051984 | positive regulation of chromosome segregation | 0.483871 | 1.93E-19 | 8.68E-18 | 7.28E-18 |
| GO:0045931 | positive regulation of mitotic cell cycle | 0.181159 | 2.06E-19 | 9.15E-18 | 7.67E-18 |
| GO:1902749 | regulation of cell cycle G2/M phase transition | 0.192 | 2.63E-19 | 1.15E-17 | 9.64E-18 |
| GO:0045005 | DNA-templated DNA replication maintenance of fidelity | 0.321429 | 3.56E-19 | 1.54E-17 | 1.29E-17 |
| GO:0090307 | mitotic spindle assembly | 0.25974 | 4.34E-19 | 1.85E-17 | 1.55E-17 |
| GO:1901992 | positive regulation of mitotic cell cycle phase transition | 0.215686 | 5.80E-19 | 2.44E-17 | 2.05E-17 |
| GO:0010389 | regulation of G2/M transition of mitotic cell cycle | 0.198198 | 4.05E-18 | 1.68E-16 | 1.41E-16 |
| GO:1905820 | positive regulation of chromosome separation | 0.466667 | 6.07E-18 | 2.50E-16 | 2.09E-16 |
| GO:0007143 | female meiotic nuclear division | 0.333333 | 1.76E-17 | 7.16E-16 | 6.00E-16 |
| GO:0044786 | cell cycle DNA replication | 0.348837 | 8.18E-17 | 3.28E-15 | 2.75E-15 |
| GO:0051988 | regulation of attachment of spindle microtubules to  kinetochore | 0.545455 | 1.24E-16 | 4.90E-15 | 4.11E-15 |
| GO:0035825 | homologous recombination | 0.265625 | 1.33E-16 | 5.20E-15 | 4.36E-15 |

| GO:0000910 | cytokinesis | 0.134409 | 3.34E-16 | 1.29E-14 | 1.09E-14 |
| --- | --- | --- | --- | --- | --- |
| GO:0051315 | attachment of mitotic spindle microtubules to kinetochore | 0.48 | 9.52E-16 | 3.65E-14 | 3.06E-14 |
| GO:0071103 | DNA conformation change | 0.214286 | 1.06E-15 | 4.02E-14 | 3.37E-14 |
| GO:0031297 | replication fork processing | 0.294118 | 1.53E-15 | 5.74E-14 | 4.81E-14 |
| GO:0032392 | DNA geometric change | 0.226667 | 2.43E-15 | 9.01E-14 | 7.56E-14 |
| GO:0032465 | regulation of cytokinesis | 0.2 | 3.87E-15 | 1.42E-13 | 1.19E-13 |
| GO:0007098 | centrosome cycle | 0.151079 | 7.40E-15 | 2.68E-13 | 2.25E-13 |
| GO:0031570 | DNA integrity checkpoint signaling | 0.15 | 8.58E-15 | 3.08E-13 | 2.58E-13 |
| GO:0090231 | regulation of spindle checkpoint | 0.413793 | 8.97E-15 | 3.18E-13 | 2.67E-13 |
| GO:0007131 | reciprocal meiotic recombination | 0.263158 | 9.72E-15 | 3.38E-13 | 2.83E-13 |
| GO:0140527 | reciprocal homologous recombination | 0.263158 | 9.72E-15 | 3.38E-13 | 2.83E-13 |
| GO:0051231 | spindle elongation | 0.75 | 1.24E-14 | 4.28E-13 | 3.59E-13 |
| GO:0000281 | mitotic cytokinesis | 0.191011 | 5.07E-14 | 1.73E-12 | 1.45E-12 |
| GO:0007292 | female gamete generation | 0.121053 | 5.15E-14 | 1.73E-12 | 1.45E-12 |
